# The selective NLRP3 inhibitor MCC950 hinders atherosclerosis development by attenuating inflammation and pyroptosis in macrophages

**DOI:** 10.1038/s41598-021-98437-3

**Published:** 2021-09-29

**Authors:** Wenyun Zeng, Danbin Wu, Yingxin Sun, Yanrong Suo, Qun Yu, Miao Zeng, Qing Gao, Bin Yu, Xijuan Jiang, Yijing Wang

**Affiliations:** 1 School of Integrative Medicine, Tianjin University of Traditional Chinese Medicine, Tianjin, 301617 China; 2 Department of Rheumatology, The First Affiliated Hospital of Guangzhou University of Chinese Medicine, Guangzhou, 510000 China; 3 Lingnan Medical Research Center of Guangzhou University of Chinese Medicine, First Clinical Medical School, Guangzhou University of Chinese Medicine, Guangzhou, 510000 China; 4 Traditional Chinese Medicine Department, Ganzhou People’s Hospital, Ganzhou, 341000 China; 5 School of Nursing, Tianjin University of Traditional Chinese Medicine, Tianjin, 301617 China

**Keywords:** Cardiovascular biology, Atherosclerosis, Pharmacology, Pharmacology, Small molecules

## Abstract

NLRP3 inflammasome is a vital player in macrophages pyroptosis, which is a type of proinflammatory cell-death and takes part in the pathogenesis of atherosclerosis. In this study, we used apoE^−/−^ mice and ox-LDL induced THP-1 derived macrophages to explore the mechanisms of MCC950, a selective NLRP3 inhibitor in treating atherosclerosis. For the in vivo study, MCC950 was intraperitoneal injected to 8-week-old apoE^−/−^ mice fed with high-fat diet for 12 weeks. For the in vitro study, THP-1 derived macrophages were treated with ox-LDL and MCC950 for 48 h. MCC950 administration reduced plaque areas and macrophages contents, but did not improve the serum lipid profiles in aortic root of apoE^−/−^ mice. MCC950 inhibited the activation of NLRP3/ASC/Caspase-1/GSDMD-N axis, and alleviated macrophages pyroptosis and the production of IL-1β and IL-18 both in aorta and in cell lysates. However, MCC950 did not affect the expression of TLR4 or the mRNA levels of NLRP3 inflammasome and its downstream proteins, suggesting that MCC950 had no effects on the priming of NLRP3 inflammasome activation in macrophages. The anti-atherosclerotic mechanisms of MCC950 on attenuating macrophages inflammation and pyroptosis involved in inhibiting the assembly and activation of NLRP3 inflammasome, rather than interrupting its priming.

## Introduction

Atherosclerosis is a major cause of death and disability worldwide. Sterile inflammation plays important roles in the development of early and advanced atherosclerosis^1^. NOD-like receptor family, pyrin domain-containing protein 3 (NLRP3) inflammasome is a key mediator of sterile inflammation^2^. The NLRP3 inflammasome consisted of NLRP3, apoptosis-associated speck-like protein containing a CARD (ASC) and Caspase‐1, which can be activated by either pattern-associated molecular patterns (PAMPs), such as lipopolysaccharide or damage-associated molecular patterns (DAMPs), such as ox-LDL, and cholesterol crystals etc^3^. NLRP3 inflammasome activation requires two steps: priming and assembly of the multimeric complex. The priming step leads to up-regulated transcription of NLRP3, IL-1β and IL-18, and enhances NLRP3 post-translational silencing^4^. The subsequent activation step leads to NLRP3 inflammasome assembly that facilitates self-cleavage and activation of Caspase-1, which further accelerates the maturation of IL-1β and IL-18 precursors and cleaves Gasdermin D (GSDMD) into active form amino terminal GSDMD (GSDMD-N)^5^. As the central effector of GSDMD-N, the formation of membrane pores by GSDMD-N causes IL-1β and IL-18 releasing and pyroptosis, which is characterized by pore formation on plasma membrane, DNA fragmentation and cells swelling^6^.

Mice that are lack of either NLRP3 or IL-1β display reduced atherosclerosis lesion, and blockade of the IL-1 receptor results in decreased lesion size^7^. Interestingly, the Canakinumab Anti-inflammatory Thrombosis Outcome Study (CANTOS) reported that targeting IL-1β, by its monoclonal antibody in clinic effectively reduced incidences of cardiovascular accidents, post-myocardial infarction mortality and residual inflammation^8^. In addition, administration of the neutralizing antibody of IL-1β, gevokizumab, relieved overall plaque burden in apoE^−/−^ mice with high fat diet^9^. Moreover, gene deletion of IL-18 slowed atherosclerotic advancement in apoE^−/−^ mice^10^. However, Canakinumab was associated with a higher incidence of fatal infection than was placebo, while gevokizumab failed to reduce intraocular inflammation in patients with Behçet's disease uveitis (BDU)^11^, suggesting that the effects of inhibiting pro-inflammatory cytokines alone are not ideal.

Macrophages largely accumulate in atherosclerotic lesions during inflammation, which is a major contributor to atherosclerosis development^12^. In early atherosclerosis, accumulation of ox-LDL induces macrophages dysfunction which forms foam cells and production of pro-inflammatory cytokines^13^. Ox-LDL can promote vascular inflammation via targeting TLR4 and thus induce the priming and activation of NLRP3 inflammasome in human macrophages^14^. On molecular level, the aberrant activation of NLRP3 inflammasome and its consequent high circulating levels of IL-1β and IL-18 are associated with macrophages recruitment to aortic wall lesions, which in turn induce foam cells formation and plaque development^15^. What’s more, pyroptosis is involved in ox-LDL induced macrophage death through activating NLRP3 inflammasome which is important for the formation of necrotic core and plaque instability in advanced atherosclerotic lesions^16^. The transcription of NLRP3-inflammasome components are upregulated and pyroptosis related proteins co-localizes with macrophages in human atherosclerotic plaques^17^. Therefore, inhibition of NLRP3 inflammasome in macrophages could exert potential therapeutic effect against atherosclerosis^18^.

Recently, a diarylsulfonylurea-containing compound MCC950, is known as one of the most potent and selective inhibitor of NLRP3 inflammasome^19^. MCC950 specifically inhibited activation of NLRP3 but not the AIM2, NLRC4 or NLRP1 inflammasomes^20^. In mouse and human macrophages, MCC950 blocked both canonical and non-canonical NLRP3 inflammasome activation and IL-1β production by abrogating ASC oligomerization^20^. Presently studies have confirmed that MCC950 is a promising anti-atherosclerotic agent. MCC950 reduced carotid artery plaque size, promoted plaque stability and improved vascular function in apoE^−/−^ mice^21^. MCC950 treatment reduced intraplaque macrophage contents and plaque lesions in a short-term model of atherosclerosis^22^. However, the effect of MCC950 on ox-LDL induced NLRP3 inflammasome activization and pyroptosis is not yet clear. Therefore, we treat apoE^−/−^ mice and THP-1 derived macrophages with MCC950 to explore its effect on atherosclerosis in this study.

## Results

### MCC950 attenuated atherosclerotic plaque formation

**Figure 1:**
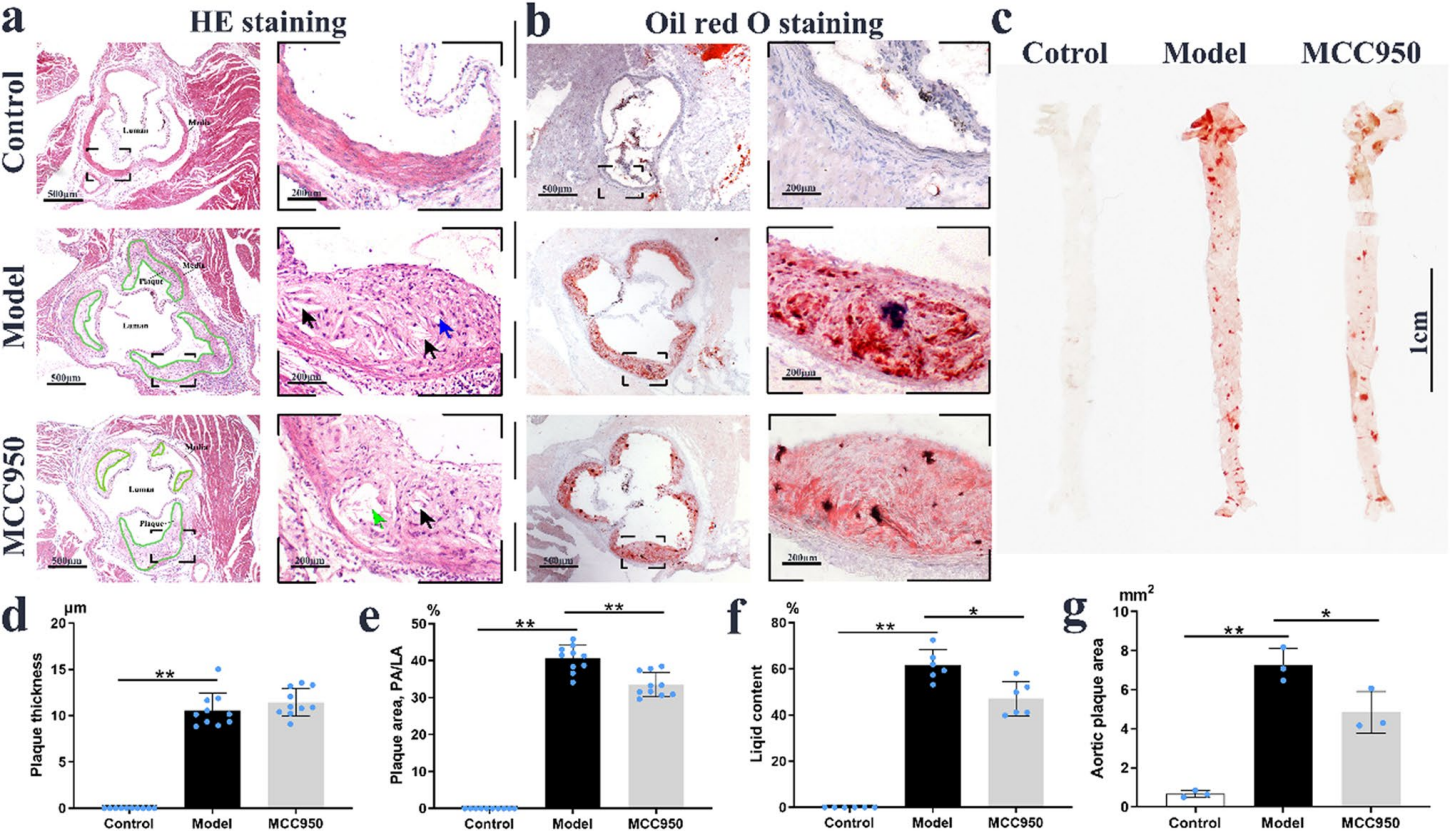
MCC950 reduced atherosclerotic plaque formation. Representative images of aortic roots with HE staining (**a**), Oil Red O staining (**b**) and the whole aorta with Oil Red O staining (**c**) were shown. The arrows indicated foam cell (blue), cholesterol crystal (black) and lipid core (green). The plaque thickness (**d**, n = 10/group), plaque area (PA/LA; **e**, n = 10/group), lipid content (**f**, n = 6/group) and aortic plaque area (**g**, n = 3/group) were quantitatively analysed. The original magnification was ×40 (scale bar = 500 μm), while the high magnification was ×100 (scale bar = 200 μm). Data were expressed as mean ± SD. *P < 0.05 was considered significant, **P < 0.01.

The body weight of apoE^−/−^ mice fed with western diets for 12 weeks was significantly heavier than that in C57BL/6 mice with chow diets (*P* < 0.01), however MCC950 treatment did not affect the body weight (Supplementary Fig. IIb). Compared to Control group, the intima layer of aortic sinus was significantly thicker but media layer was significantly thinner in Model group. What’s more, plaque deposition including foam cells, cholesterol crystals, and necrotic core were observed in the intima layer in Model group (Fig. 1a,b). After MCC950 treatment for 12 weeks, the plaque areas of whole aortas were significantly reduced in MCC950 group compared to that in Model group (4.84 ± 1.07 mm^2^ *vs* 7.25 ± 0.86 mm^2^; *P* < 0.01; Fig. 1c,g). Similarly, the plaque areas (PA/LA) and lipid contents of aortic root were dramatically lower by 17.53% and 23.55% in MCC950 group (*P* < 0.05, *P* < 0.01; Fig. 1e,f) than those in Model group. However, there was no significant difference in the plaque thickness between MCC950 group and Model group (*P* = 0.27; Fig. 1d). On the other hand, MCC950 didn’t provoke toxicities or other phenotypic changes of liver, kidney and bone marrow to the apoE^−/−^ mice (Supplementary Fig. IIa,c,d).

### MCC950 reduced serum pro-inflammatory cytokines rather than lipid profiles

**Figure 2:**
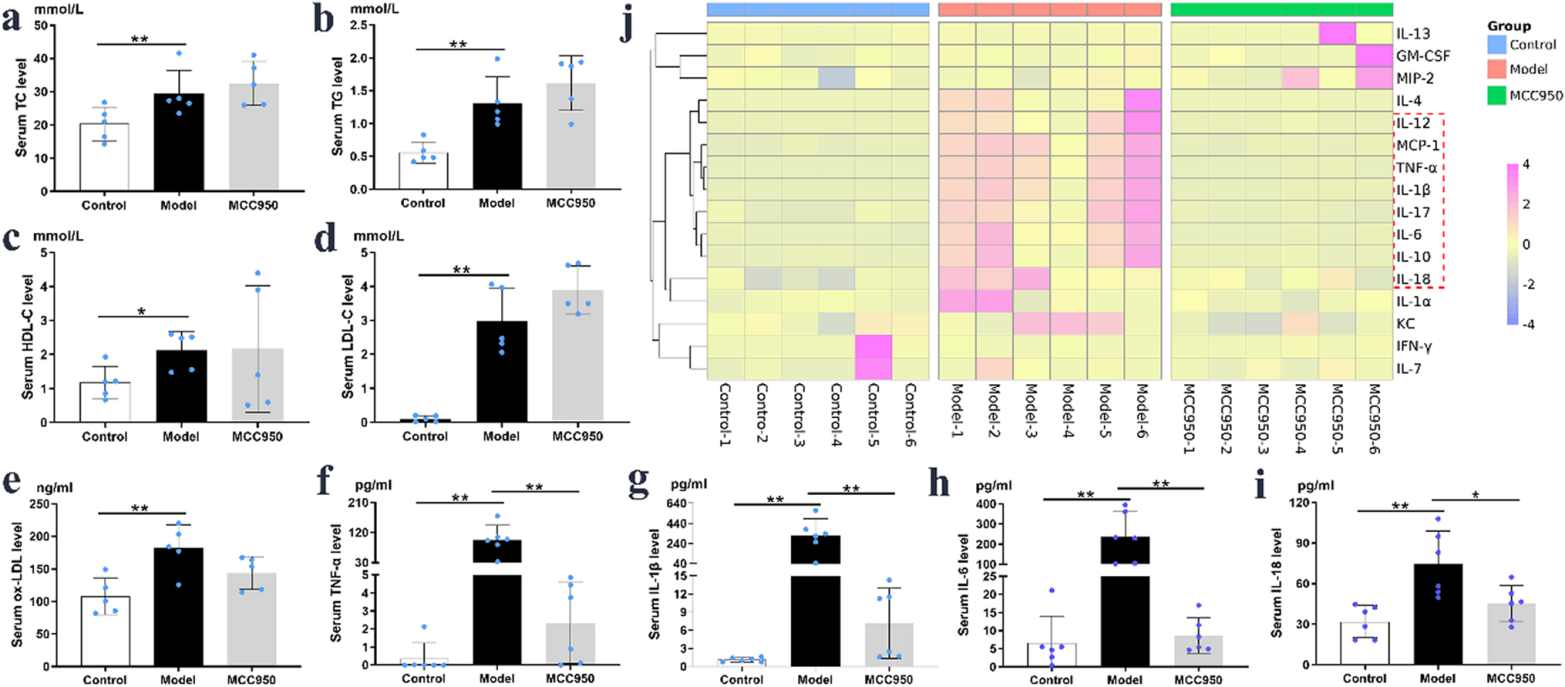
MCC950 reduced serum cytokines with no effect on lipid profile. The serum levels of TG, TC, LDL-C, HDL-C and ox-LDL (n = 5/group) were shown on (**a**–**e**). Serum concentrations of cytokines which were measured by Luminex and presented as heat map (n = 6/group; **j**). Serum levels of TNF-α, IL-1β, IL-6 and IL-18 were selected from the liquid chip results and presented (**f**–**i**). Data were presented as mean ± SD. *P < 0.05 was considered significant, **P < 0.01.

As shown in Fig. 2, serum levels of TG, TC, LDL-C, HDL-C and ox-LDL were significantly higher in the model group compared to those in control group (*P* < 0.01; Fig. 2a–e). Nevertheless, addition of MCC950 did not lead to significant changes in serum lipid levels. After 12 weeks of high-fat diet, the heat map (Fig. 2j) revealed that the serum levels of pro-inflammatory factors, in particular TNF-α, IL-1β, IL-6 and IL-18, were increased in model group compared with those in control group (*P* < 0.01; Fig. 2f–i), which was significantly attenuated after treatment with MCC950 (*P* < 0.05).

### MCC950 inhibited NLRP3 inflammasome activation in the aorta

**Figure 3:**
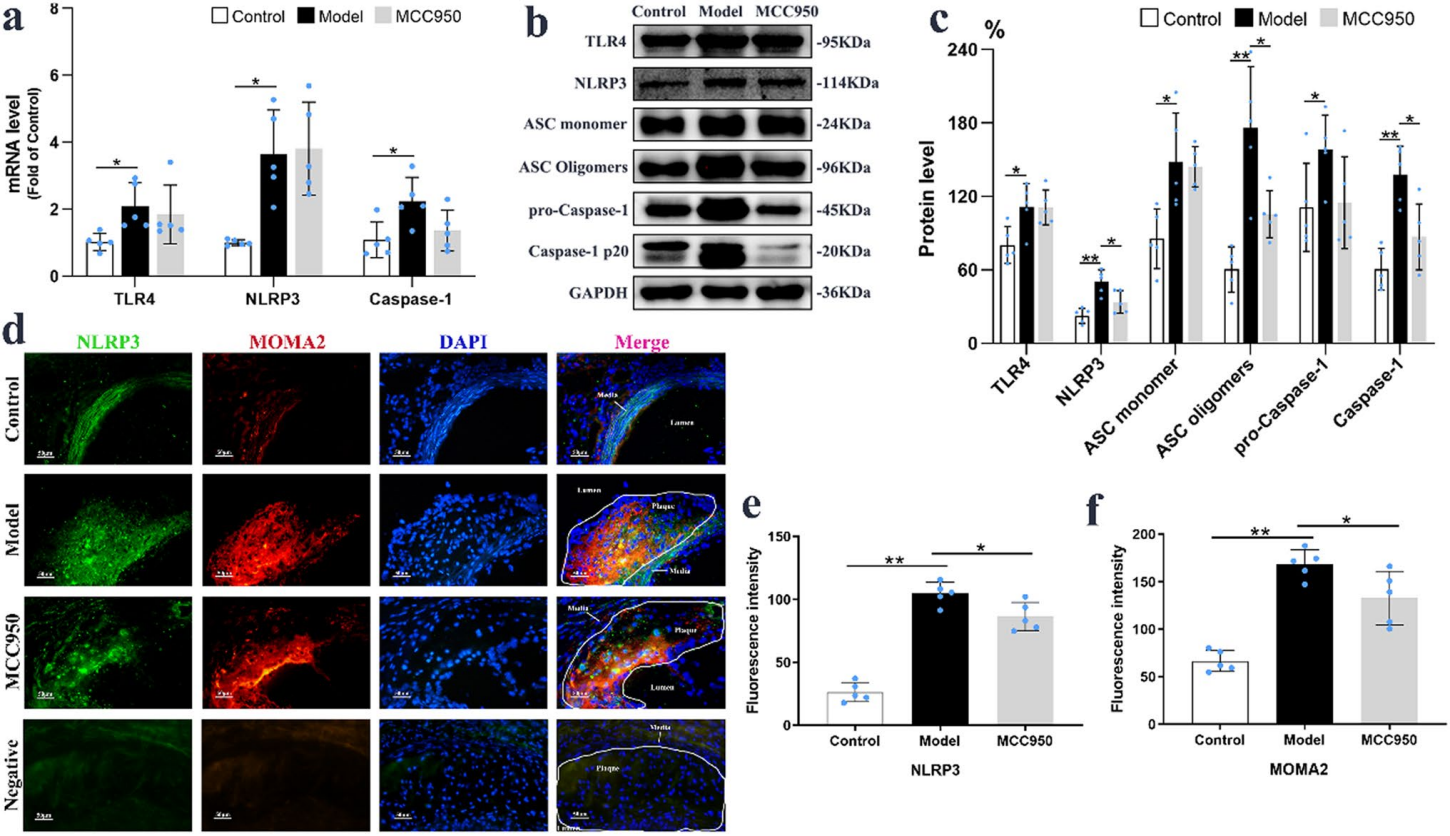
MCC950 inhibited NLRP3 inflammasome activation of macrophages in the aorta. The mRNA levels of TLR4, NLRP3 and Caspase-1 in aorta (n = 5/group) were shown on (**a**), while the protein levels of TLR4, NLRP3, ASC monomer, ASC oligomers, pro-Caspase-1 and Caspase-1 (n = 5/group) were exhibited on (**b**,**c**). The fluorescence stained images and quantitative analysis of NLRP3 (green), MOMA2 (red) in aortic root of mice (n = 5/group) were displayed on (**d**–**f**). GAPDH served as an internal control. The magnification was ×400, Scale bar = 50 μm. Data were presented as mean ± SD. *P < 0.05 was considered significant, **P < 0.01.

The mRNA and protein levels of TLR4, NLRP3 and Caspase-1 were all increased in the Model group compared to Control group (*P* < 0.05; Fig. 3a–c), but there were no abnormal changes on the mRNA levels of other inflammasome like NLRP1, NLRC4, AIM2 (Supplementary Fig. IIId–f) and Caspase-3 and Caspase-11 (Supplementary Fig. IVd,e) in apoE^−/−^ mice. Then MCC950 administration significantly lowered the protein expressions of NLRP3 (33.96 ± 9.19% *vs* 50.14 ± 9.89%; *P* = 0.028) and Caspase-1 (86.98 ± 26.83% *vs* 137.87 ± 23.31%; *P* = 0.013) which in turn blocked ASC oligomerization (105.50 ± 19.20% *vs* 175.92 ± 50.12%; *P* = 0.019) to inhibit NLRP3 inflammasome activation. However, MCC950 intervention neither affected the transcription levels of TLR4, NLRP3 and Caspase-1 nor the translation levels of TLR4 and pro-Caspase-1 in the aorta (Fig. 3a–c). This result suggested that MCC950 inhibited NLRP3 inflammasome activation through regulating protein translation or post-translational modification. What’s more, MCC950 significantly reduced intra-macrophage NLRP3 expression (86.43 ± 11.34 vs 104.73 ± 8.96; *P* = 0.022) and macrophage content (132.96 ± 28.09 vs 168.58 ± 15.19; *P* = 0.037) in aortic root of apoE^−/−^ mice (Fig. 3d–f). Immunofluorescence co-localization analysis demonstrated that NLRP3 was mainly expressed in the macrophages of the vessel wall.

### MCC950 mitigated NLRP3 mediated macrophages pyroptosis within AS plaque

**Figure 4:**
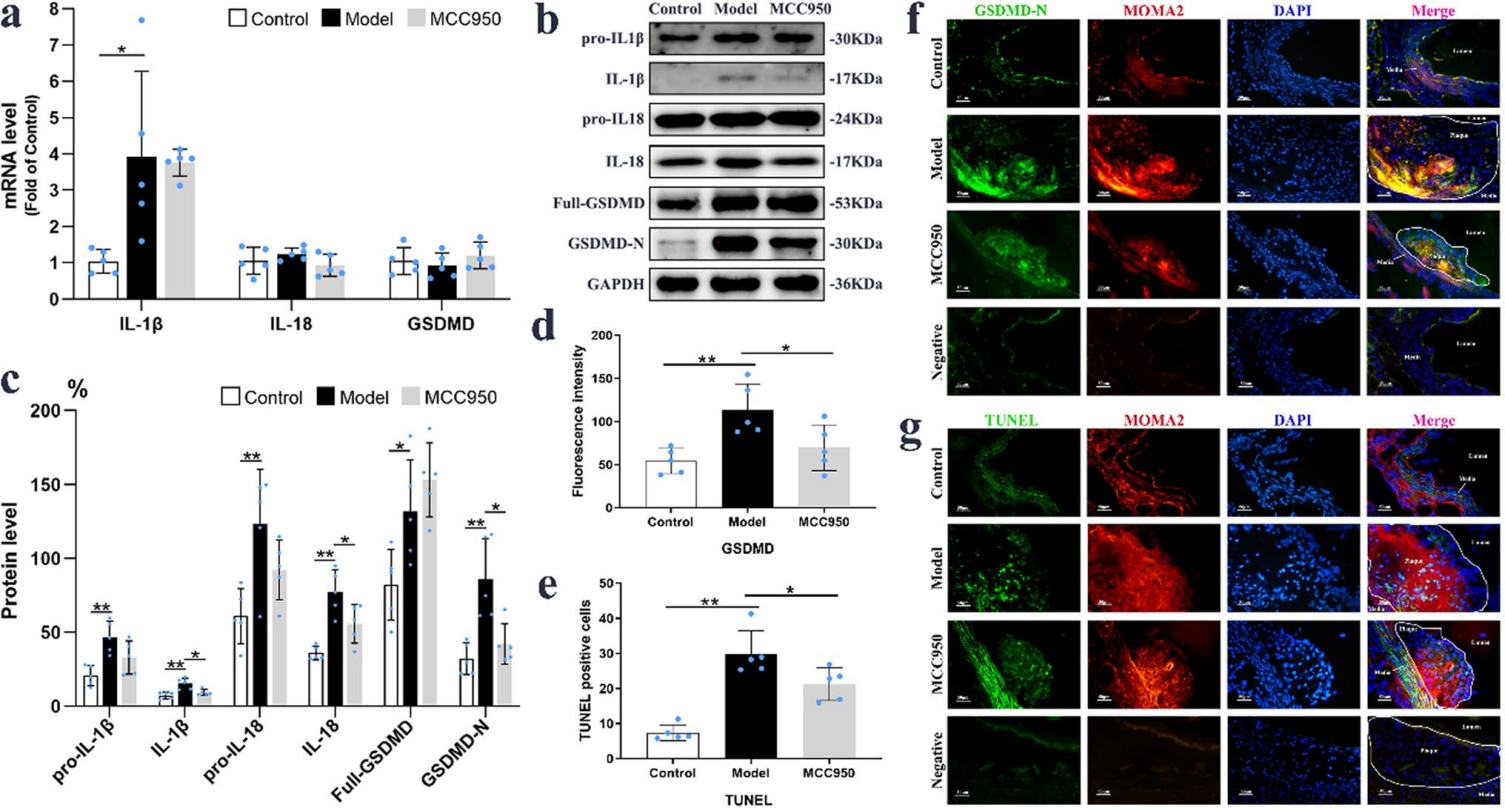
MCC950 mitigated NLRP3 mediated pyroptosis of macrophages within AS plaque. The mRNA and protein (n = 5/group) levels of IL-1β, IL-18, GSDMD-N in aorta were quantified and shown on (**a**–**c**). The representative images of GSDMD-N (green), MOMA2 (red) and DAPI (blue) in aortic root with immunofluorescent staining (n = 5/group) were exhibited on (**d**,**f**). TUNEL (green) and MOMA2 (red) stained pictures (n = 5/group) in atherosclerotic plaques and quantitation of TUNEL were shown on (**e**,**g**). GAPDH served as an internal control. The magnification was ×400, Scale bar = 50 μm. Data were presented as mean ± SD. *P < 0.05 was considered significant, **P < 0.01.

After activation of NLRP3 inflammasome, downstream proteins including IL-1β, IL-18, GSDMD were dissociated to active forms to trigger pyroptosis. As shown on Fig. 4, the mRNA level of IL-1β and protein levels of IL-1β, pro-IL-18, IL-18, Full-GSDMD, GSDMD-N were obviously elevated in the aorta of Model group (*P* < 0.05; Fig. 4a–c). MCC950 attenuated the upregulation of IL-1β (9.42 ± 1.99% *vs* 15.35 ± 3.71%; *P* = 0.014), IL-18 (55.83 ± 13.01% *vs* 77.33 ± 15.03%; *P* = 0.042) and GSDMD-N (42.11 ± 13.64% *vs* 85.92 ± 27.54%; *P* = 0.013) in model group, but had no significant effects on precursive expressions of these proteins and mRNA expression of TNF-α (Supplementary Fig. Ic). In addition, the fluorescence intensity of GSDMD-N was pronouncedly upregulated in macrophages within aorta of Model group (113.74 ± 29.76 *vs* 54.24 ± 14.63; *P* = 0.004), which was reversed by MCC950 treatment (69.73 ± 26.67 *vs* 113.74 ± 29.76; *P* = 0.039; Fig. 4d,f). Furthermore, TUNEL staining demonstrated that increased DNA fragments (TUNEL positive cells on Fig. 4g) in Model group were markedly lowered in MCC950 group (21.19 ± 4.64 *vs* 29.91 ± 6.54; *P* = 0.041; Fig. 4e). The above results implied that MCC950 could prevent aortic macrophages pyroptosis in AS.

### MCC950 inhibited ox-LDL induced NLRP3 inflammasome activation in macrophages

**Figure 5:**
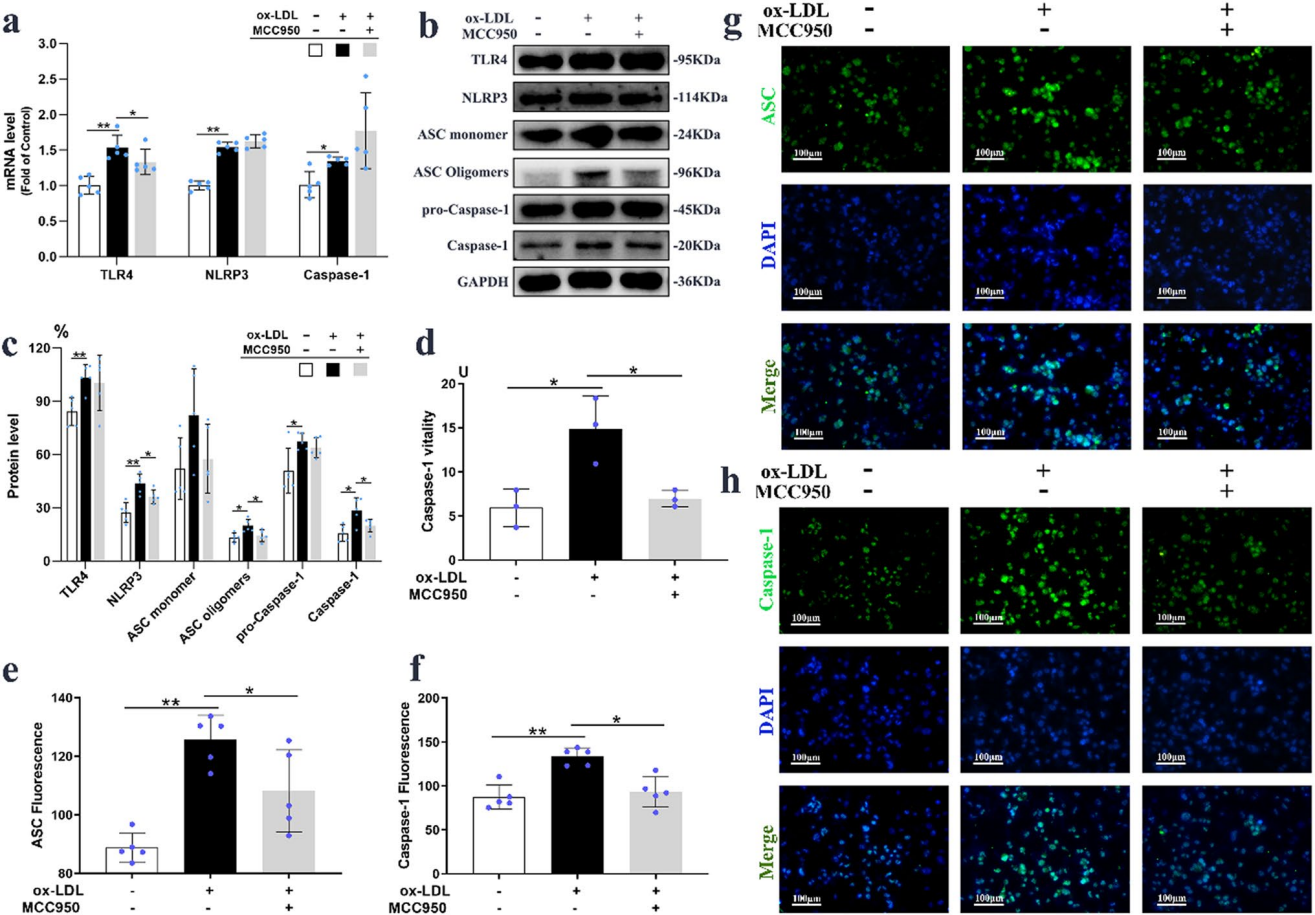
MCC950 inhibited ox-LDL induced NLRP3 inflammasome activation in macrophages. The mRNA and protein (n = 5/group) expressions of TLR4, ASC, NLRP3, Caspase-1 in ox-LDL induced macrophages were shown on (**a**–**c**). The vitality of Caspase-1 was measured and presented on (**d**). The representative images and quantitation of ASC and Caspase-1 by immunofluorence staining (n = 5/group) were exhibited on (**e**–**h**). GAPDH served as an internal control. The magnification was ×200, Scale bar = 100 μm. Data were presented as mean ± SD. *P < 0.05 was considered significant, **P < 0.01.

As depicted in Fig. 5a–c, the mRNA and protein expressions of TLR4, NLRP3, Caspase-1 were significantly enhanced (*P* < 0.01), however there were no effects on the mRNA levels of NLRP1, NLRC4, AIM2, Caspase-3, Caspase-4 and Caspase-5 (Supplementary Figs. IIIa–c and IVa–c) in 100 μg/ml ox-LDL induced macrophages. Addition of MCC950 markedly inhibited NLRP3 inflammasome activation through down-regulating NLRP3 expression (36.28 ± 3.83% *vs* 43.41 ± 5.66%; *P* = 0.048), reducing ASC oligomerization (14.15 ± 3.34% *vs* 19.81 ± 3.58%; *P* = 0.032), restraining Caspase-1 cleavage (19.94 ± 3.66% *vs* 28.38 ± 7.13%; *P* = 0.046; Fig. 5c). However, the ox-LDL stimulated transcriptional regulations of TLR4, NLRP3 and Caspase-1 were not affected by MCC950 pretreatment (Fig. 5a), which is consistently with in vivo results. The elevated vitality of Caspase-1 was reduced by 53.5% in the lysates with MCC950-treated macrophages compared with that in Model group (*P* = 0.024; Fig. 5d), suggested that MCC950 inhibited the ox-LDL induced activation of Caspase-1. Moreover, ASC oligomerization (Fig. 5e,g) and Caspase-1 activation (Fig. 5f,h) were observed by immunofluorescence staining which were enhanced in ox-LDL induced macrophages (*P* < 0.01), while MCC950 could clearly prevent these changes (*P* < 0.05).

### MCC950 inhibited ox-LDL induced macrophages pyroptosis in vitro

**Figure 6:**
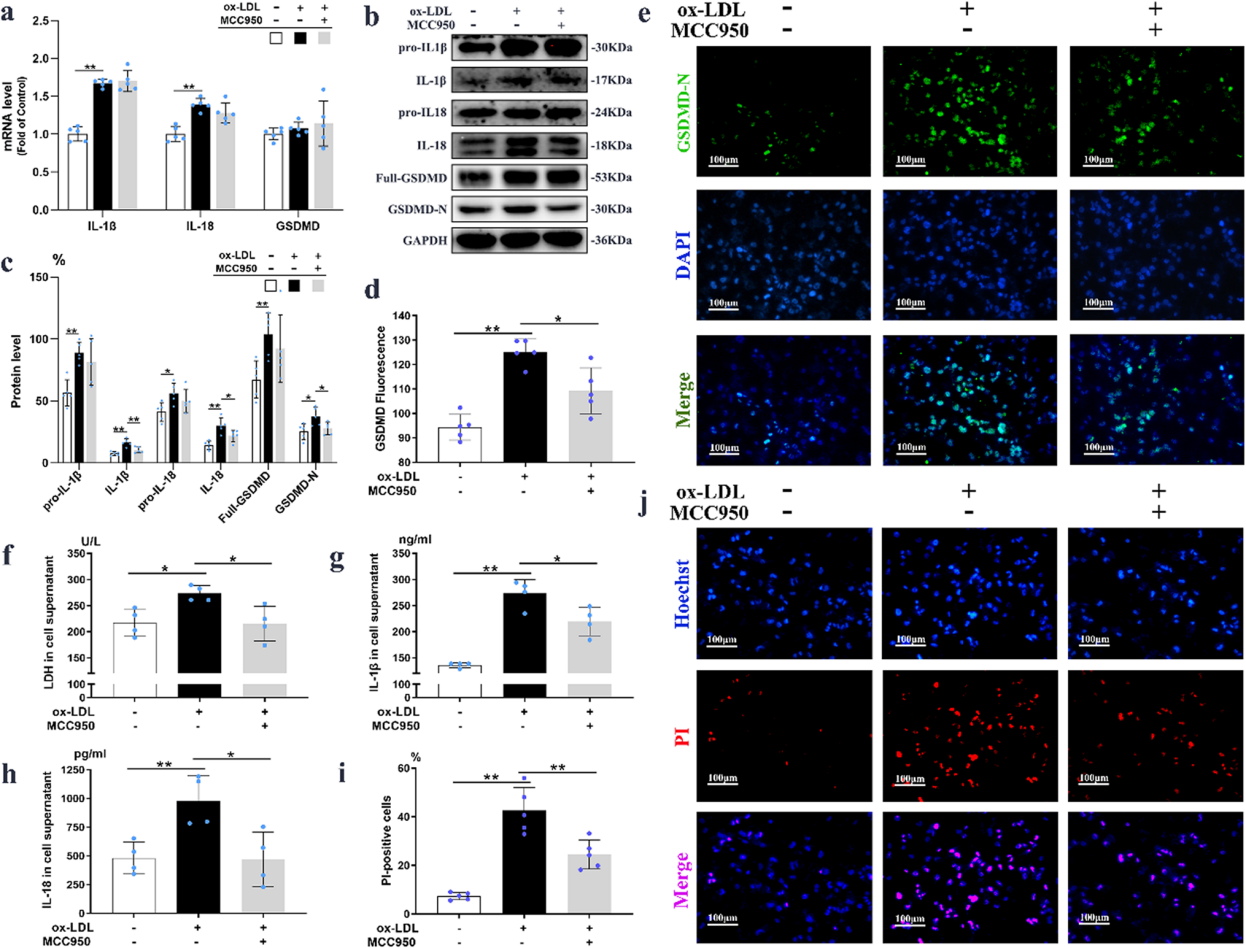
MCC950 inhibited ox-LDL induced macrophages pyroptosis in vitro. The mRNA and protein (n = 5/group) expressions of IL-1β, IL-18, GSDMD-N in ox-LDL induced macrophages were shown on (**a**–**c**). The representative images and quantitation of GSDMD-N by immunofluorence staining (n = 5/group) were presented on (**d**,**e**). The releases of LDH, IL-1β and IL-18 were measured by LDH assay kit (**f**) and corresponding ELISA kits (**g**,**h**). The representative images and positive rates of Immunofluorescence staining with Hoechst 33,342 (blue) and PI (red) were presented on (**i**,**j**) (n = 5/group). GAPDH served as an internal control. The magnification was ×200, Scale bar = 100 μm. Data were presented as mean ± SD. *P < 0.05 was considered significant, **P < 0.01.

As shown on Fig. 6b,c, the protein levels of IL-1β, IL-18, GSDMD-N were severally upregulated by 217.8% (16.38 ± 3.08% *vs* 7.52 ± 1.54%; *P* = 0.000), 213.8% (30.02 ± 6.28% *vs* 14.04 ± 3.52%; *P* = 0.001) and 148.0% (37.48 ± 7.28% *vs* 25.33 ± 6.41%; *P* = 0.023) in ox-LDL induced THP-1 macrophages, which were reversed by 1 μM MCC950 administration (*P* < 0.05). However, MCC950 treatment did not affect the mRNA transcription level and their precursor protein expressions of IL-1β, IL-18, and GSDMD-N in cell lysates (Fig. 6a). Correspondingly, the fluorescence intensity of GSDMD-N was prominently increased in ox-LDL induced macrophages (125.18 ± 5.23 *vs* 94.37 ± 5.27; *P* = 0.000), which was reversed by MCC950 treatment (109.29 ± 9.37 *vs* 125.18 ± 5.23; *P* = 0.011; Fig. 6d,e). As the consequence of forming pyroptotic pore by GSDMD-N, the releases of LDH, IL-1β and IL-18 in cell supernatants were increased by 25.8% (273.55 ± 14.94 *vs* 217.39 ± 26.46 U/L; *P* = 0.010; Fig. 6f), 100.81% (273.16 ± 26.49 *vs* 136.04 ± 4.86 ng/ml; *P* = 0.000; Fig. 6g) and 132.44% (982.09 ± 219.81 *vs* 482.98 ± 140.00 pg/ml; *P* = 0.020; Fig. 6h). Meanwhile, MCC950 addition blocked the releases of all these factors (*P* < 0.05). Moreover, the Hoechst33342/PI staining (Fig. 6i,j) results showed that MCC950 could significantly decrease the percentage of PI-positive cells in macrophages (*P* = 0.007). Interestingly, MCC950 exposure did not affect TNF-α transcription and secretion by THP-1 macrophages upon stimulation with ox-LDL (Supplementary Fig. Ia,b). Collectively, the above data indicated that MCC950 inhibited ox-LDL induced macrophages pyroptosis.

## Discussion

Ox-LDL accumulation is able to activate the NLRP3 inflammasome in macrophages within aortic plague^23^. Pyroptosis involved in ox-LDL induced human macrophage death through the promotion of Caspase-1 activation is important for the formation of unstable plaques in atherosclerosis^24^. In this study, we observed that the activation of NLRP3 inflammasome and macrophages pyroptosis were enhanced in the aorta of apoE^−/−^ mice with high-fat diet. What’s more, ox-LDL increased the expression of NLRP3 rather than NLRP1, NLRC4 and AIM2, and then initiated ASC oligomerization and pro-Caspase-1 recruitment to assemble NLRP3 inflammasome complex in THP-1-derived macrophages, which resulted in Caspase-1 activation and then maturation of IL-1β, IL-18 and GSDMD dissociation. Moreover, ox-LDL was found to facilitate membranolysis, DNA fragmentation and inflammation release of macrophages, thereby it mediate pyroptosis^25^. The finding was consistent with our results that revealed upregulation of GSDMD-N, and the increased release of LDH, IL-1β, IL-18 in ox-LDL induced macrophages.

As our results showed MCC950, a potent selective NLRP3 inhibitor, is active both in mice and in human cells lines. Some research demonstrated that MCC950 treatment significantly reduced the development of atherosclerotic lesions as determined by maximal stenosis, average plaque size, and plaque volume in the carotid artery of apoE^−/−^ mice^22^. Other study had shown that MCC950 significantly attenuated atherosclerotic plaques formation, lowered systemic inflammation, decreased macrophages content in the aortic arches of apoE^−/−^ mice^26^. In this study, we exhibited that MCC950 has the effect of anti-atherosclerosis by inhibiting plaque formation in the aortas of apoE^−/−^ mice with high fat diet for 12 weeks. Furthermore, MCC950 reduced the serum levels of pro-inflammatory factors in particular TNF-α, IL-1β, IL-6, IL-18, with no effect on serum lipid profiles and ox-LDL level in apoE^−/−^ mice, suggesting that MCC950 had a well anti-inflammatory effect on preventing atherosclerosis.

In this study, we found that MCC950 treatment decreased the serum level of TNF-α in Western-type diet fed apoE^−/−^ mice, however MCC950 did not improve the mRNA level of TNF-α in aortas of apoE^−/−^ mice and ox-LDL induced THP-1 macrophages, and the release of TNF-α in the cell supernatant. As the NLRP3 inflammasome acts independently of TNF-α, the NLRP3 inhibitor MCC950 should have no effect on the expression of TNF-α. However, some studies have shown that MCC950 had a reduction in TNF-α expression of several tissues. For example, aortic gene expression of TNF-α was significantly upregulated after 10 weeks of diabetes, which was significantly attenuated by MCC950^21^. MCC950 treatment (10 mg/kg) reduced the protein expressions of TNF-α in the hippocampus of aged mice after surgery^27^. Furthermore, protein level of TNF-α was significantly reduced in the left basal cortical of subarachnoid hemorrhage rats after receiving MCC950^28^. Agampodi et al. suggested that MCC950 could attenuate TNF-α expression in the distal colon of spontaneous colitis mice, but did not change the serum level of TNF-α^29^. Combined with the above researches, the opposite role of MCC950 on TNF-α may be ascribed to the body and tissue specificity, and more experimental models are needed to elucidate the anti-inflammatory effect of MCC950*.*

**Figure 7:**
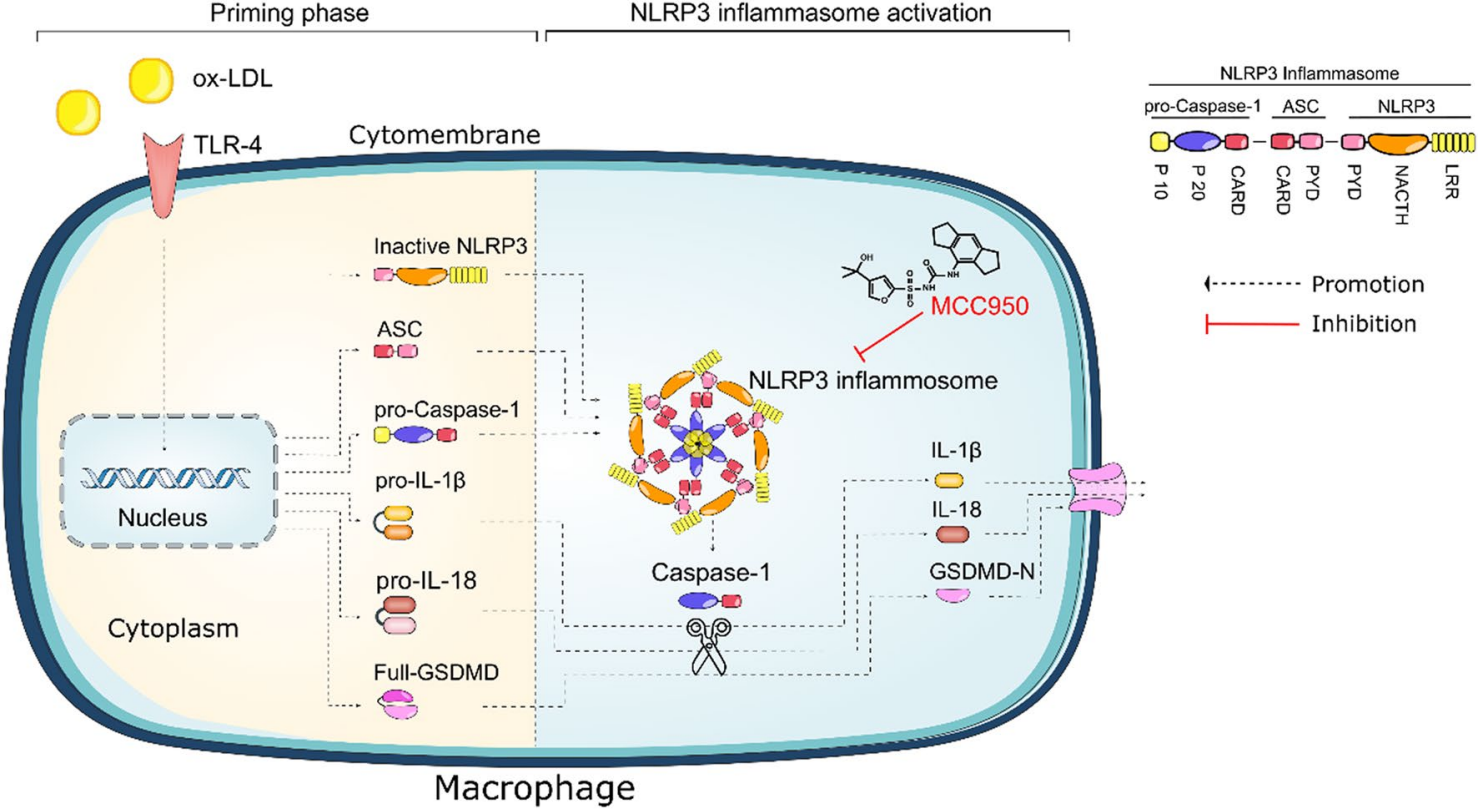
Ox-LDL can be recognized by pattern recognition receptor such as TLR-4, which in turn induce the upregulation of NLRP3, pro-caspase-1, GSDMD, pro-IL-1β, and pro-IL-18 (priming step) in macrophages. Then, NLRP3 would associate with ASC and pro-caspase-1 to form multi-protein complexes, which was referred as NLRP3 inflammasome (priming step). MCC950 inhibits NLRP3 activation and blocks ASC oligomerization and pro-Caspase1 cleavage into active Caspase-1 (activation step). As a result, Caspase-1 dependent pyroptosis and the formation of IL-1β, IL-18 and GSDMD-N in ox-LDL induced THP-1 macrophages are impeded.

It’s well known that NLRP3 inflammasome plays an important role in atherosclerotic inflammation^30^. However, MCC950 did not affect mRNA transcription levels of NLRP3 or NLRP3-related genes in the carotid arteries^22^. Furthermore, the LPS-stimulated inductions of NF-κB and pro-IL-1β in bone marrow derived macrophages were not impacted by MCC950 pretreatment^20^. These findings suggested that MCC950 did not inhibit the priming phase of NLRP3 activation. In our study, we found that MCC950 administration affected neither the expression levels of TLR4 nor the transcription levels of NLRP3 and its downstream protein in both mouse aorta and ox-LDL induced macrophages. Besides, we showed that MCC950 addition attenuated the upregulation of NLRP3, Caspase-1, IL-1β, IL-18, GSDMD-N and ASC oligomerization, but had no significant effects on pro-caspase-1, pro-IL-1β, and pro-IL-18 expressions of both in vivo and in vitro AS model. These results were consistent with other report that used vascular smooth muscle cells by ox-LDL^31^. Our data suggested that MCC950 inhibited ox-LDL induced NLRP3 inflammasome activation and pyroptosis based on regulating protein translation or post-translational modification of NLRP3, ASC, Caspase-1, IL-1β, IL-18 and GSDMD (Fig. 7).

NLRP3 protein is consist of a C-terminal leucine-rich repeat (LRR) domain, a central nucleotide-binding and oligomerization domain (NOD, also known as NACHT domain), and an amino-terminal pyrin domain (PYD)^32^. Recent research revealed that MCC950 specifically binded to the NLRP3 ATP hydrolysis (or Walker B) motif within the NACHT domain to prevent NLRP3 activation and IL-1β maturation^33^. Further study indicated that MCC950 binded to a region that crossed Walker B with partial Walker A to affect the conformation and oligomerization of NLRP3^34^. In addition, other study showed that NLRP3-activating stimuli opened NLRP3 to its active conformation by separating the N terminal from the C terminal, but MCC950 kept NLRP3 active conformation in inactive state^35^. Combing in vivo and in vitro results, we pointed out that MCC950 could ameliorate ox-LDL induced NLRP3 activation and inflammasome assembly in macrophages. However, the binding site of MCC950 to NLRP3 upon ox-LDL stimulation and its downstream mechanism needed to be further elucidated.

## Conclusion

Our findings showed that MCC950 alleviated atherosclerotic plaque formation and systemic inflammation, primarily via inhibiting the activation of NLRP3 inflammasome and macrophages pyroptosis in both apoE^−/−^ mice with high fat diet and ox-LDL induced THP-1 macrophages. The mechanism of MCC950 on anti-atherosclerosis mainly involved in inhibiting the step of NLRP3 inflammasome activation rather than its priming step.

## Materials and methods

### Materials

Trypsinase, Penicillin–Streptomycin, Roswell Park Memorial Institute (RPMI) 1640 medium and fetal bovine serum (FBS) were obtained from Gibco (New York, USA). Phorbol 12-myristate-13-acetate (PMA) was purchased from Biogems (New Jersey, USA). MCC950 was purchased from APExBIO (Houston, USA). Ox-LDL was purchased from Yiyuan Biotech Inc (YB-002-1, Guangzhou, China). The primary antibodies against IL-1β and GAPDH were purchased from Abcam (Cambridge, UK). The antibodies against TLR-4, ASC, Caspase-1 and GSDMD were purchased from Santa Cruz (Dallas, USA). The primary antibodies against NLRP3 and IL18 were purchased from Invitrogen (Carlsbad, USA). High-fat diets were purchased from Jiangsu Medicience bio-pharmaceutical Co., Ltd. All reagents used in this study were of analytical grade.

### Animal husbandry

15 Eight-week old male C57BL/6J and 30 age-matched male apolipoprotein E-deficient (apoE^−/−^) mice were purchased from Beijing Vital River Laboratory Animal Technologies Co. Ltd. All mice were raised in standard cages under controlled light/dark cycle of 12 h at 22 ± 2 °C and humidity of 45 ± 5%. After a week of acclimatization, C57BL/6J mice were fed with normal diet as Control group, while apoE^−/−^ mice that were fed with high-fat diet (0.15% cholesterol + 21% fat) were randomly assigned into Model group (administrated with normal saline) and MCC950 group (intraperitoneal injection of 10 mg/kg MCC950 three times a week for 12 weeks, simultaneously with the high fat diet, n = 15). After 6 weeks of high fat diet, the complete aortas of two mice in each group were stained by *en face* Oil red O to evaluate atherosclerosis progression. After 12 weeks of treatment, venous bloods were immediately drawn from ophthalmic plexus of mice, while the heart and whole aortic tree were harvested. Three aortas of each group were randomly selected for *en-face* Oil red O staining (n = 3), while half of the remaining 10 aortas were used for RT-qPCR (n = 5) and half for Western blot (n = 5). Six of the 10 hearts were frozen sectioned into aortic root for HE (n = 6), Oil red O (n = 6), immunofluorescence (n = 5) and TUNEL staining (n = 5), while the others of hearts were paraffin sectioned into aortic root for HE staining (n = 4).

### Cell culture

The myeloid leukemia mononuclear cells, THP-1, were obtained from American Type Culture Collection (ATCC) and cultivated in RPMI-1640 media supplemented with 10% FBS and 1% antibiotics (penicillin 100 U/ml; streptomycin 100 mg/ml). THP-1 cells were cultured at density of 1 × 10^6^ cells/ml in a humidified chamber with 5% CO_2_ at 37 °C. The macrophages induction from THP-1 cells were achieved by being incubated in RPMI‐1640 medium with 100 ng/ml PMA and 0.3% bovine serum albumin (BSA) for 24 h. Then, THP-1 derived macrophages were incubated with MCC950 (1 μM), followed by 100 μg/ml ox‐LDL for 48 h to establish in vitro model of pyroptosis.

### Aortic lesion assessment

The full-length of aorta including iliac bifurcation was dissected out and opened along the ventral midline. *En face* preparations were dipped in 60% isopropyl-alcohol, and stained with 0.6% Oil-Red-O solution for 15 min. The hearts were fixed with 4% paraformaldehyde and then cut in half parallel to the left auricular appendage. The sections containing aortic sinus were embedded in OCT medium (kept at 4 °C) and sliced to thickness of 10 μm. Hematoxylin–eosin and Oil Red O stainings were used to estimate the size of aortic lesions. Histological images were captured with a digital camera mounted on a Leica microscope and parameters were analyzed using Image J software (Version 1.8.0, https://imagej.en.softonic.com/).

### Serum analysis

Serums were harvested by spinning of whole blood at 3000 rpm for 15 min at 4 °C. Then, serum levels of TC, TG, LDL-C, HDL-C and ox-LDL were determined using commercially available kits (Nanjing Jiancheng Bioengineering Institute, Nanjing, China) under manufacturer’s instructions. Meanwhile, the serum levels of inflammatory factors (GM-CSF, IL-1α, IL-1β, IL-4, IL-6, IL-10, IL-12, IL-13, IL-17, IFN-γ, KC, MCP-1, MIP-2 and TNF-α) were assessed using Luminex xMAP technology according to manufacturer’s instructions. The results were presented as heat maps.

### RNA purification and real-time qPCR

Aortic and cellular RNAs were extracted using RNA isolation kit (Beijing TianGen Biotech Co., Ltd) following manufacture’s manual. The total RNAs were reverse transcribed into cDNA using the FastQuant RT kit (Beijing TianGen Biotech Co., Ltd) in 20 μl reaction volumes containing 2 μg of total RNAs. Real-time qPCR was then performed using 7500 Fast Real-Time PCR System (Carlsbad, USA) and the cycling program included an initial denaturation step at 95 °C for 15 min followed by 40 cycles (95 °C for 10 s, 60 °C for 30 s, and 72 °C for 1 min). The relative levels of mRNAs were assessed by 2^−ΔΔCT^ method. All primers for RT-qPCR were synthesized by Sangon Biotech (Shanghai) Co., Ltd and shown in Tables 1, 2 and Supplementary Tables I, II. The CT values of each targeted gene in vivo and in vitro were shown on supplementary files.

**Table 1:** Oligonuceotide sequences for *Mus musculus* gene expansion.

Name	Primer sequence	Products ( bp)	Gene ID
TLR-4	Forward: GCTTTCACCTCTGCCTTCAC	174	21,898
	Reverse: GAAACTGCCATGTTTGAGCA		
NLRP3	Forward: ATGCTGCTTCGACATCTCCT	196	216,799
	Reverse: AACCAATGCGAGATCCTGAC		
Caspase-1	Forward: CACAGCTCTGGAGATGGTGA	207	12,362
	Reverse: TCTTTCAAGCTTGGGCACTT		
IL-1β	Forward: GCCCATCCTCTGTGACTCAT	230	16,176
	Reverse: AGGCCACAGGTATTTTGTCG		
IL-18	Forward: GACAGCCTGTGTTCGAGGAT	188	16,173
	Reverse: TGGATCCATTTCCTCAAAGG		
GSDMD-N	Forward: TGCGTGTGACTCAGAAGACC	204	69,146
	Reverse: ATAAAGCTCCAGGCAGCGTA

**Table 2:** Oligonuceotide sequences for *Homo sapiens* gene expansion.

Name	Primer sequence	Products (bp)	Gene ID
TLR-4	Forward: GATAGCGAGCCACGCATTCA	167	7099
	Reverse: TTAGGAACCACCTCCACGCA		
NLRP3	Forward: CTTCTCTGATGAGGCCCAAG	200	114,548
	Reverse: GCAGCAAACTGGAAAGGAAG		
Caspase-1	Forward: GCTTTCTGCTCTTCCACACC	160	834
	Reverse: CATCTGGCTGCTCAAATGAA		
IL-1β	Forward: GGGCTCAAGGCAAAGAATC	204	3553
	Reverse: TTCTGCTTGAGAGGTGCTGA		
IL-18	Forward: TGCATCAACTTTGTGGCAAT	169	3606
	Reverse: ATAGAGGCCGATTTCCTTGG		
GSDMD-N	Forward: GGTTCTGGAAACCCCGTTAT	248	79,792
	Reverse: CAAGGTGTTAGGGTCCACAC		

### Western blotting

Total proteins were extracted from aortas (in vitro study) or macrophages (in vivo study), then protein concentrations were measured by BCA Kit (Thermo Fisher Scientific, USA). The extracted proteins were separated by SDS-PAGE and then transferred to 0.4 μm PVDF membranes (Millipore, USA). After blocking with 5% skimmed milk in TBST (Tris-buffered saline with 0.1% Tween20) for 2 h at room temperature, the membranes were incubated with one of the following primary antibodies: rabbit anti-TLR4 (1:500), rabbit anti-NLRP3 (1:500), mouse anti-Caspase-1 (1:250), mouse anti-ASC (1:500), mouse anti-GSDMD (1:250), rabbit anti-IL-1β (1:500), rabbit anti-IL-18 (1:1000) and rabbit anti-GAPDH (1:10,000), overnight at 4 °C. Then, the membranes were incubated with the corresponding secondary antibodies (goat anti-rabbit IgG 1:10,000 or horse anti-mouse IgG 1:1000) for 2 h following adequate washes and visualization through Enhanced Chemiluminescence (ECL) method. The images were recorded in gel documentation system (ChemiDoc™ MP, Bio-Rad, CA). GAPDH was used as an internal control. Image J software was employed for quantification via multiple the areas of a band with its corresponding optical density. The original images of protein blots in vivo and in vitro were shown on supplementary files.

### Immunofluorescence

The frozen sections of aorta root and cell slides were permeabilized in 0.3% Triton X-100 for 30 min at 37 °C, then blocked by 10% goat serum in PBS for 30 min at room temperature. Thereafter, primary antibodies either NLRP3 (1:100) or ASC (1:100) or Caspase-1 (1:200) or GSDMD (1:100) or MOMA2 (macrophage marker, 1:200) at 4 °C overnight. Negative control staining was performed by replacing primary antibodies with PBS. Next, the sections were stained with fluorescence-labelled secondary antibody respectively for 1 h at room temperature and then counterstained with DAPI for 5 min. Finally, sections were visualized under fluorescence microscope (Olympus Microscope IX3, Japan) and fluorescence intensity was assessed through Image J software.

### Cell death assay

Pyroptosis is characterized by loss of cell membrane potential and DNA fragmentation which can be detected by TUNEL staining^36^. The frozen sections of aortic root were permeabilized with 0.3% Triton X-100 for 15 min at room temperature. Then, the TUNEL reaction mixtures from one-step TUNEL assay kit (Roche, Switzerland) were applied for 2 h at 37 °C in the dark. Subsequently, sections were sealed with goat serum for 30 min and incubated with primary antibody MOMA2 (1:200) and the corresponding secondary antibody (1:300). Finally, DAPI was used to counterstain nucleus and the samples were visualized using Olympus fluorescence microscope. Double-fluorescent staining with Hoechst 33342 and PI was used to assess the formation of membrane pores during pyroptosis. The cells were stained with Hoechst 33342 staining solution (Solarbio, Beijing) for live cells and 2 μg/ml PI for 20 min in 5% CO_2_ at 37 °C. Then the cells were washed three times with PBS. The fluorescences of Hoechst 33342 and PI were detected by a Inverted fluorescence microscope (Leica DMI6000B, Germany) and the percentage of PI-positive cells was evaluated by Image J software.

### LDH release and ELISA measurement

LDH release assay was carried out to measure the release of cell contents caused by pyroptosis. The supernatant was collected from Cell cultured medium and its LDH level was determined using LDH assay kit (Nanjing Jiancheng Bioengineering Institute, China) according to the manufacturer’s protocol, while the secretions of IL-1β, IL-18 and TNF-α in the supernatant were measured using ELISA kits (Abcam, UK). The absorbance was read at 450 nm with a microplate reader.

### Statistics

Statistical data were shown as means ± SD. SPSS software (Version 18.0, https://www.ibm.com/cn-zh/analytics/spss-statistics-software) was used to analyze data while GraphPad Prism software (version 7.0, https://www.graphpad.com/) was used to draw graphs. One-way ANOVA with Newman–Keuls multiple comparisons test was applied to normal distribution and homogeneous variance, while Dunnett’ T3 test was used to non-normal distribution. *P* < 0.05 indicates statistical significance.

### Ethical approval

The animal study was performed in accordance with ARRIVE relevant guidelines and regulations, and was approved by Tianjin University of Traditional Chinese Medicine of Medicine Animal Care and Use Committee. All methods were carried out in accordance with relevant guidelines and regulations.

## Supplementary Information

Supplementary Information.

## Abbreviations

AIM2: Absent in melanoma 2
AS: Atherosclerosis
ApoE^−/−^: Apolipoprotein E knockout
ASC: Apoptosis-associated speck-like protein containing a CARD
Caspases: Cysteinyl aspartate specific proteinases
FBS: Fetal bovine serum
GM-CSF: Granulocyte–macrophage colony stimulating factor
GSDMD: Gasdermin D
GSDMD-N: Amino terminal GSDMD
HDL-C: High density lipoprotein-cholesterol
HFD: High fat diet
IFN-γ: Interferon-gamma
IL-1β: Interleukin-1β
IL-6: Interleukin-6
IL-18: Interleukin-18
LDL-C: Low density lipoprotein-cholesterol
MCP-1: Monocyte chemoattractant protein-1
MIP-2: Macrophage inflammatory protein 2
NLRP3: Nucleotide-binding oligomerization domain-like receptor pyrin domain containing 3
NLRC4: Nucleotide-binding oligomerization domain, leucine-rich repeat and caspase recruitment domain-containing 4
Ox-LDL: Oxidized low-density lipoprotein
PMA: Phorbol 12-myristate-13-acetate
TC: Total cholesterol
TG: Triglyceride
TLR-4: Toll-like receptor 4
TNF-α: Tumour necrosis factor-α
