# Supplementary material for: The selective NLRP3 inhibitor MCC950 hinders atherosclerosis development by attenuating inflammation and pyroptosis in macrophages
Source: Sci Rep. 2021 Sep 29;11:19305. doi: 10.1038/s41598-021-98437-3 (PMC8481539; doi:10.1038/s41598-021-98437-3)

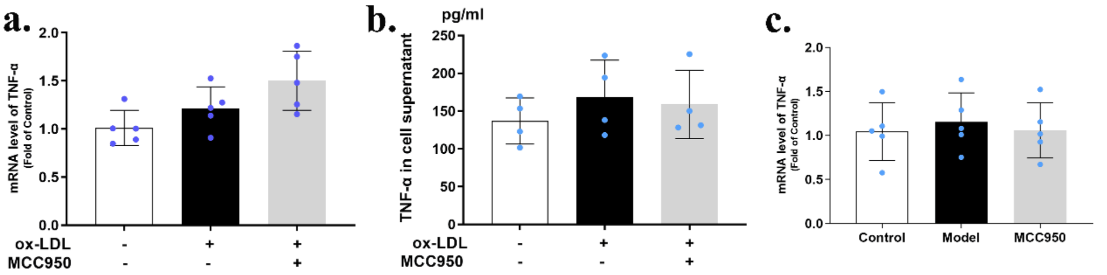

Supplementary Figure I. MCC950 exposure did not affect TNF-α transcription (a, n=5/group) and secretion (b, n=4/group) by THP-1 macrophages upon stimulation with ox-LDL. Also in vivo, MCC950 treatment did not affect mRNA level of TNF-α in the aortas of apoE$^{-/-}$ mice with high-fat diet (c, n=5/group).

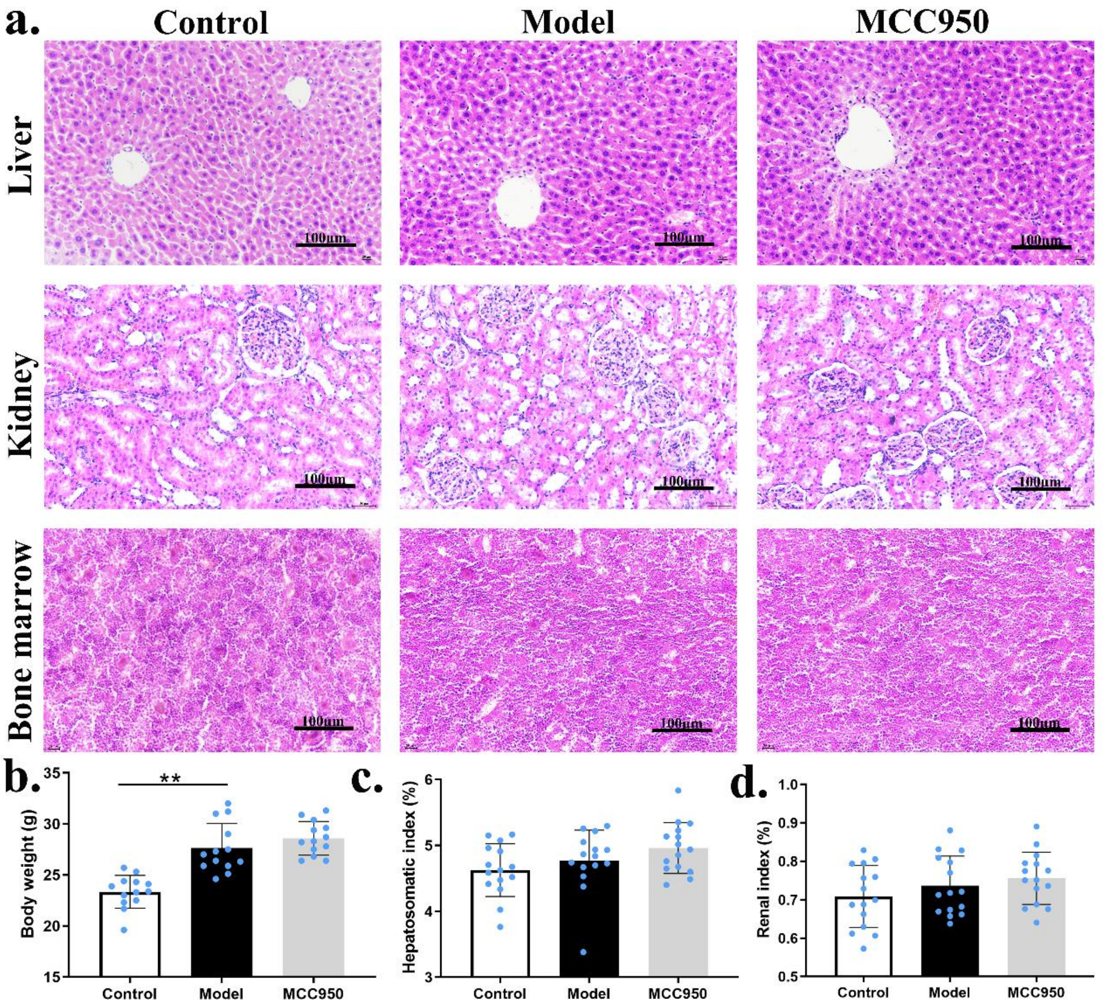

Supplementary Figure II. MCC950 treatment did not affect the body weight, hepatosomatic index and renal index of apoE$^{-/-}$ mice fed with western diets for 12 weeks (b~d, n=13). MCC950 showed a nontoxic effect on the liver, kidney and bone marrow

of apoE$^{-/-}$ mice. $^{*}P$<0.05 was considered significant, $^{**}P$<0.01.

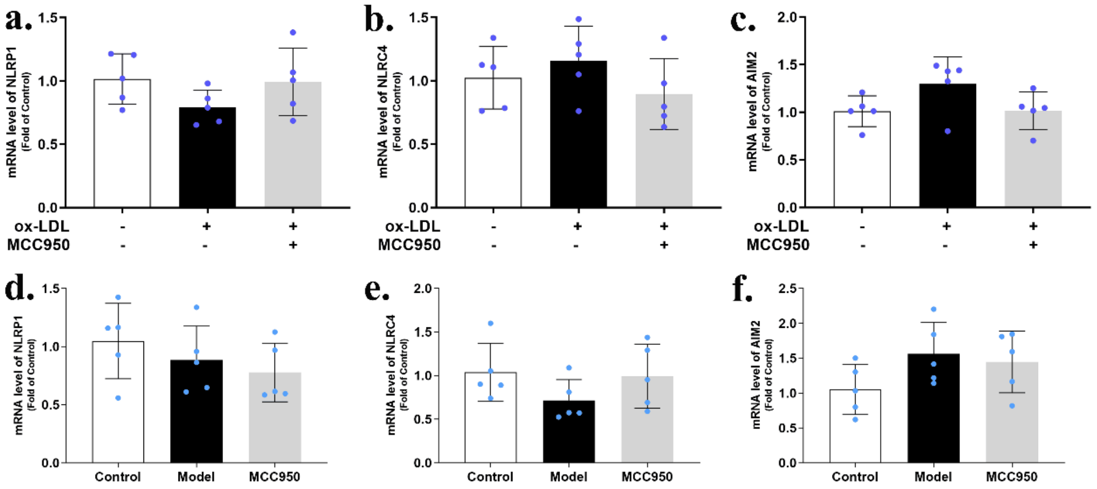

Supplementary Figure III. MCC950 treatment did not affect the mRNA levels of NLRP1 (a, d), NLRC4 (b, e) and AIM2 (c, f) in the aortas of apoE$^{-/-}$ mice with high-fat diet and in ox-LDL induced THP-1 macrophages. N=5/group.

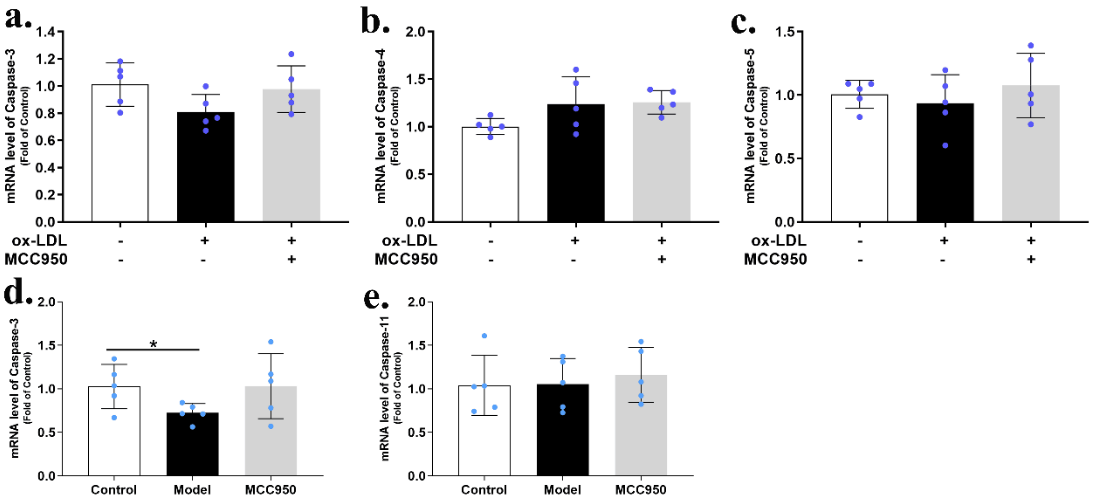

Supplementary Figure IV. MCC950 exposure did not affect the mRNA levels of Caspase-3 (a, d), Caspase-4 (also named Caspase-11 in mice; a, d) and Caspase-5 (c) in the aortas of apoE$^{-/-}$ mice with high-fat diet and in ox-LDL induced THP-1 macrophages. N=5/group, $^{*}P$<0.05 was considered significant.

Supplementary Table I. Primer sequences for *Mus musculus* gene expansion.

Name	Primer sequence	Products	Gene ID
AIM2	Forward: TCGTGATGAAATCCACCCTCA<br>Reverse: CTGTCTTGTTCCCACTGCCT	218bp	383619
Caspase-3	Forward: GGCTGACTTCCTGTATGCTTACTCTAC<br>Reverse: ATGCTGCAAAGGGACTGGATGAAC	96bp	12367
Caspase-11	Forward: TGACAAGCGTTGGGTTTTTGT<br>Reverse: CTGACTCCATGCCCTTCACCAT	123bp	12363
NLRP1	Forward: CTGCGGCAGTACCAATGAAG<br>Reverse: GCCTTTTGAGCATCTCGCAT	145bp	195046
NLRC4	Forward: TGCTGGAAGTCCTCTGGGAT<br>Reverse: GGGCTCGGCTATTGTCCTTT	174bp	268973
TNF-α	Forward: ATGAGAAGTTCCCAAATGGC<br>Reverse: CTCCACTTGGTGGTTTGCTA	125bp	21926

Supplementary Table II. Primer sequences for *Homan sapiens* gene expansion.

Name	Primer sequence	Products	Gene ID
AIM2	Forward: TAGGTTATTTGGGCATGCTCTC<br>Reverse: ACAACTTTGGGATCAGCCTCC	115bp	9447
Caspase-3	Forward: ATTTGGAACCAAAGATCATACATGG<br>Reverse: TTCCCTGAGGTTTGCTGCAT	185bp	836
Caspase-4	Forward: ACAAGACCCACGTGGAGAAG<br>Reverse: ACTTCCTCTAGGTGGCAGCA	151bp	837
Caspase-5	Forward: CACAGCCAGGGATATGGAGT<br>Reverse: GCCTGGACAATGATGACCTT	234bp	838
NLRP1	Forward: GGAGATCTCATGCCTGCAACT<br>Reverse: CCAAGACAACCTCCACCGAT	145bp	22861
NLRC4	Forward: TGCTGGAAGTCCTCTGGGAT<br>Reverse: GGGCTCGGCTATTGTCCTTT	174bp	58484
TNF-α	Forward: AGAACTCACTGGGGCCTACA<br>Reverse: GCTCCGTGTCTCAAGGAAGT	177bp	7124

mRNA level of TLR4 in aortas										
		TLR4	GAPDH							
Group	number	CT	CT	△CT	△CT Mean	△△CT	$2^{-\triangle\triangle CT}$	Mean	SD	Compared to Model
Control	C1	26.414	17.12	9.294	9.2668	0.0272	0.9813	1.026	0.2525	0.011536231
	C2	30.264	20.444	9.82		0.5532	0.6815			
	C3	27.185	17.937	9.248		-0.0188	1.0131			
	C4	24.839	15.658	9.181		-0.0858	1.0613			
	C5	27.684	18.893	8.791		-0.4758	1.3907			
Model	M1	25.277	16.615	8.662		-0.6048	1.5208	2.098	0.6913	
	M2	27.409	18.745	8.664		-0.6028	1.5187			
	M3	26.708	18.906	7.802		-1.4648	2.7603			
	M4	25.228	17.512	7.716		-1.5508	2.9298			
	M5	23.842	15.393	8.449		-0.8178	1.7627			
MCC950	MC1	25.314	16.681	8.633		-0.6338	1.5516	1.844	0.8758	0.623267759
	MC2	27.211	18.379	8.832		-0.4348	1.3517			
	MC3	27.148	19.648	7.5		-1.7668	3.403			
	MC4	28.423	19.633	8.79		-0.4768	1.3917			
	MC5	26.514	17.851	8.663		-0.6038	1.5197			
**mRNA level of NLRP3 in aortas**										
		NLRP3	GAPDH							
Group	number	CT	CT	△CT	△CT Mean	△△CT	$2^{-\triangle\triangle CT}$	Mean	SD	Compared to Model
Control	C1	31.969	17.12	14.849	14.6984	0.1506	0.9009	1.003	0.0869	0.00200095
	C2	34.981	20.444	14.537		-0.1614	1.1184			
	C3	32.702	17.937	14.765		0.0666	0.9549			
	C4	30.269	15.658	14.611		-0.0874	1.0625			
	C5	33.623	18.893	14.73		0.0316	0.9783			
Model	M1	30.272	16.615	13.657		-1.0414	2.0582	3.649	1.3117	
	M2	31.74	18.745	12.995		-1.7034	3.2567			
	M3	31.207	18.906	12.301		-2.3974	5.2685			
	M4	29.978	17.512	12.466		-2.2324	4.6992			
	M5	28.525	15.393	13.132		-1.5664	2.9616			
MCC950	MC1	29.668	16.681	12.987		-1.7114	3.2748	3.808	1.387	0.856668074
	MC2	31.593	18.379	13.214		-1.4844	2.798			
	MC3	31.84	19.648	12.192		-2.5064	5.682			
	MC4	32.059	19.633	12.426		-2.2724	4.8313			
	MC5	31.254	17.851	13.403		-1.2954	2.4545			
**mRNA level of Caspase-1 in aortas**										
		Caspase-1	GAPDH							
Group	number	CT	CT	△CT	△CT Mean	△△CT	$2^{-\triangle\triangle CT}$	Mean	SD	Compared to Model
Control	C1	26.805	17.12	9.685	9.1252	0.5598	0.6784	1.085	0.5315	0.018340287
	C2	29.645	20.444	9.201		0.0758	0.9488			
	C3	26.92	17.937	8.983		-0.1422	1.1036			
	C4	25.277	15.658	9.619		0.4938	0.7102			
	C5	27.031	18.893	8.138		-0.9872	1.9823			
Model	M1	24.671	16.615	8.056		-1.0692	2.0983	2.246	0.7009	
	M2	26.156	18.745	7.411		-1.7142	3.2811			
	M3	26.987	18.906	8.081		-1.0442	2.0622			
	M4	26.203	17.512	8.691		-0.4342	1.3512			
	M5	23.232	15.393	7.839		-1.2862	2.4388			
MCC950	MC1	24.608	16.681	7.927		-1.1982	2.2945	1.361	0.6126	0.066192176
	MC2	27.937	18.379	9.558		0.4328	0.7408			
	MC3	28.891	19.648	9.243		0.1178	0.9216			
	MC4	28.105	19.633	8.472		-0.6532	1.5727			
	MC5	26.624	17.851	8.773		-0.3522	1.2765			

**mRNA level of IL-1β in aortas**										
		IL-1β	GAPDH							
Group	number	CT	CT	△CT	△CT Mean	△△CT	$2^{-\triangle\triangle CT}$	Mean	SD	Compared to Model
Control	C1	30.018	17.12	12.898	12.401	0.497	0.7086	1.042	0.3257	0.026586322
	C2	32.343	20.444	11.899		-0.502	1.4162			
	C3	30.253	17.937	12.316		-0.085	1.0607			
	C4	27.675	15.658	12.017		-0.384	1.305			
	C5	31.768	18.893	12.875		0.474	0.72			
Model	M1	27.617	16.615	11.002		-1.399	2.6372	3.926	2.3558	
	M2	29.489	18.745	10.744		-1.657	3.1536			
	M3	30.63	18.906	11.724		-0.677	1.5988			
	M4	27.725	17.512	10.213		-2.188	4.5567			
	M5	24.852	15.393	9.459		-2.942	7.6848			
MCC950	MC1	27.039	16.681	10.358		-2.043	4.121	3.76	0.3722	0.880304777
	MC2	28.828	18.379	10.449		-1.952	3.8691			
	MC3	30.09	19.648	10.442		-1.959	3.8879			
	MC4	30.111	19.633	10.478		-1.923	3.7921			
	MC5	28.605	17.851	10.754		-1.647	3.1318			
**mRNA level of IL-18 in aortas**										
		IL-18	GAPDH							
Group	number	CT	CT	△CT	△CT Mean	△△CT	$2^{-\triangle\triangle CT}$	Mean	SD	Compared to Model
Control	C1	29.158	17.12	12.038	11.1586	0.8794	0.5436	1.059	0.3712	0.356720243
	C2	31.138	20.444	10.694		-0.4646	1.3799			
	C3	29.133	17.937	11.196		0.0374	0.9744			
	C4	26.91	15.658	11.252		0.0934	0.9373			
	C5	29.506	18.893	10.613		-0.5456	1.4596			
Model	M1	27.386	16.615	10.771		-0.3876	1.3082	1.238	0.1744	
	M2	29.326	18.745	10.581		-0.5776	1.4924			
	M3	29.765	18.906	10.859		-0.2996	1.2308			
	M4	28.604	17.512	11.092		-0.0666	1.0472			
	M5	26.397	15.393	11.004		-0.1546	1.1131			
MCC950	MC1	27.455	16.681	10.774		-0.3846	1.3055	0.934	0.3054	0.088820744
	MC2	29.856	18.379	11.477		0.3184	0.802			
	MC3	30.527	19.648	10.879		-0.2796	1.2139			
	MC4	31.27	19.633	11.637		0.4784	0.7178			
	MC5	29.677	17.851	11.826		0.6674	0.6296			
**mRNA level of GSDMD in aortas**										
		GSDMD	GAPDH							
Group	number	CT	CT	△CT	△CT Mean	△△CT	$2^{-\triangle\triangle CT}$	Mean	SD	Compared to Model
Control	C1	28.921	17.12	11.801	11.2256	0.5754	0.6711	1.049	0.3739	0.594691014
	C2	30.956	20.444	10.512		-0.7136	1.6399			
	C3	29.003	17.937	11.066		-0.1596	1.117			
	C4	27.204	15.658	11.546		0.3204	0.8008			
	C5	30.096	18.893	11.203		-0.0226	1.0158			
Model	M1	28.479	16.615	11.864		0.6384	0.6424	0.922	0.3478	
	M2	29.475	18.745	10.73		-0.4956	1.4099			
	M3	30.366	18.906	11.46		0.2344	0.85			
	M4	28.559	17.512	11.047		-0.1786	1.1318			
	M5	27.41	15.393	12.017		0.7914	0.5778			
MCC950	MC1	27.13	16.681	10.449		-0.7766	1.7131	1.205	0.3715	0.249179745
	MC2	29.461	18.379	11.082		-0.1436	1.1047			
	MC3	31.087	19.648	11.439		0.2134	0.8625			
	MC4	30.313	19.633	10.68		-0.5456	1.4596			
	MC5	29.251	17.851	11.4		0.1744	0.8861			

mRNA level of TNF-α in aortas									
		TNF-α	GAPDH						
Group	number	CT	CT	△CT	△CT Mean	△△CT	$2^{-\triangle\triangle CT}$	Mean	SD	Compared to Model
Control	C1	29.28	17.12	12.16	12.149	0.011	0.9924	1.045	0.328	0.620000045
	C2	32.01	20.444	11.566		-0.583	1.498			
	C3	30.012	17.937	12.075		-0.074	1.0526			
	C4	28.599	15.658	12.941		0.792	0.5775			
	C5	30.896	18.893	12.003		-0.146	1.1065			
Model	M1	28.054	16.615	11.439		-0.71	1.6358	1.153	0.3313	
	M2	31.307	18.745	12.562		0.413	0.7511			
	M3	30.689	18.906	11.783		-0.366	1.2888			
	M4	29.648	17.512	12.136		-0.013	1.0091			
	M5	27.431	15.393	12.038		-0.111	1.08			
MCC950	MC1	28.94	16.681	12.259		0.11	0.9266	1.058	0.314	0.654804412
	MC2	31.104	18.379	12.725		0.576	0.6708			
	MC3	31.591	19.648	11.943		-0.206	1.1535			
	MC4	31.175	19.633	11.542		-0.607	1.5231			
	MC5	29.976	17.851	12.125		-0.024	1.0168			

mRNA level of Caspase-3 in aortas									
		Caspase-3	GAPDH						
Group	number	CT	CT	△CT	△CT Mean	△△CT	$2^{-\triangle\triangle CT}$	Mean	SD	Compared to Model
Control	C1	27.194	17.12	10.074	9.954	0.12	0.9202	1.027	0.255	0.039932916
	C2	30.347	20.444	9.903		-0.051	1.036			
	C3	28.47	17.937	10.533		0.579	0.6694			
	C4	25.393	15.658	9.735		-0.219	1.1639			
	C5	28.418	18.893	9.525		-0.429	1.3463			
Model	M1	27.051	16.615	10.436		0.482	0.716	0.725	0.1052	
	M2	28.947	18.745	10.202		0.248	0.8421			
	M3	29.686	18.906	10.78		0.826	0.5641			
	M4	27.957	17.512	10.445		0.491	0.7115			
	M5	25.685	15.393	10.292		0.338	0.7911			
MCC950	MC1	26.41	16.681	9.729		-0.225	1.1688	1.03	0.3729	0.11626482
	MC2	27.71	18.379	9.331		-0.623	1.5401			
	MC3	29.958	19.648	10.31		0.356	0.7813			
	MC4	29.463	19.633	9.83		-0.124	1.0898			
	MC5	28.615	17.851	10.764		0.81	0.5704			

mRNA level of Caspase-11 in aortas									
		Caspase11	GAPDH						
Group	number	CT	CT	△CT	△CT Mean	△△CT	$2^{-\triangle\triangle CT}$	Mean	SD	Compared to Model
Control	C1	26.684	17.12	9.564	9.6116	-0.0476	1.0335	1.04	0.3449	0.942141407
	C2	30.397	20.444	9.953		0.3414	0.7893			
	C3	27.512	17.937	9.575		-0.0366	1.0257			
	C4	25.699	15.658	10.041		0.4294	0.7426			
	C5	27.818	18.893	8.925		-0.6866	1.6095			
Model	M1	25.836	16.615	9.221		-0.3906	1.3109	1.055	0.293	
	M2	28.818	18.745	10.073		0.4614	0.7263			
	M3	28.417	18.906	9.511		-0.1006	1.0722			
	M4	27.457	17.512	9.945		0.3334	0.7937			
	M5	24.547	15.393	9.154		-0.4576	1.3733			
MCC950	MC1	26.181	16.681	9.5		-0.1116	1.0804	1.16	0.3151	0.599948163
	MC2	28.108	18.379	9.729		0.1174	0.9218			
	MC3	28.633	19.648	8.985		-0.6266	1.5439			
	MC4	28.727	19.633	9.094		-0.5176	1.4316			
	MC5	27.742	17.851	9.891		0.2794	0.8239			

**mRNA level of NLRP1 in aortas**										
		NLRP1	GAPDH							
Group	number	CT	CT	△CT	△CT Mean	△△CT	$2^{-\triangle\triangle CT}$	Mean	SD	Compared to Model
Control	C1	28.76	17.12	11.64	10.8004	0.8396	0.5588	1.048	0.3248	0.426513503
	C2	31.024	20.444	10.58		-0.2204	1.1651			
	C3	28.843	17.937	10.906		0.1056	0.9294			
	C4	26.245	15.658	10.587		-0.2134	1.1594			
	C5	29.182	18.893	10.289		-0.5114	1.4254			
Model	M1	26.995	16.615	10.38		-0.4204	1.3383	0.884	0.2928	
	M2	30.258	18.745	11.513		0.7126	0.6102			
	M3	30.332	18.906	11.426		0.6256	0.6482			
	M4	28.374	17.512	10.862		0.0616	0.9582			
	M5	26.404	15.393	11.011		0.2106	0.8642			
MCC950	MC1	27.525	16.681	10.844		0.0436	0.9702	0.778	0.2524	0.558207752
	MC2	29.926	18.379	11.547		0.7466	0.596			
	MC3	31.151	19.648	11.503		0.7026	0.6145			
	MC4	31.207	19.633	11.574		0.7736	0.585			
	MC5	28.481	17.851	10.63		-0.1704	1.1254			

**mRNA level of NLRC4 in aortas**										
		NLRC4	GAPDH							
Group	number	CT	CT	△CT	△CT Mean	△△CT	$2^{-\triangle\triangle CT}$	Mean	SD	Compared to Model
Control	C1	26.372	17.12	9.252	9.1024	0.1496	0.9015	1.037	0.3335	0.116651412
	C2	28.869	20.444	8.425		-0.6774	1.5993			
	C3	27.475	17.937	9.538		0.4356	0.7394			
	C4	24.928	15.658	9.27		0.1676	0.8903			
	C5	27.92	18.893	9.027		-0.0754	1.0537			
Model	M1	26.523	16.615	9.908		0.8056	0.5721	0.715	0.2377	
	M2	27.723	18.745	8.978		-0.1244	1.0901			
	M3	28.937	18.906	10.031		0.9286	0.5254			
	M4	26.916	17.512	9.404		0.3016	0.8114			
	M5	25.295	15.393	9.902		0.7996	0.5745			
MCC950	MC1	25.854	16.681	9.173		0.0706	0.9522	0.993	0.3683	0.193728481
	MC2	28.244	18.379	9.865		0.7626	0.5894			
	MC3	28.381	19.648	8.733		-0.3694	1.2918			
	MC4	29.266	19.633	9.633		0.5306	0.6923			
	MC5	26.429	17.851	8.578		-0.5244	1.4383			

**mRNA level of AIM2 in aortas**										
		AIM2	GAPDH							
Group	number	CT	CT	△CT	△CT Mean	△△CT	$2^{-\triangle\triangle CT}$	Mean	SD	Compared to Model
Control	C1	25.938	17.12	8.818	9.4044	-0.5864	1.5015	1.051	0.3588	0.081256245
	C2	29.468	20.444	9.024		-0.3804	1.3017			
	C3	27.296	17.937	9.359		-0.0454	1.032			
	C4	25.751	15.658	10.093		0.6886	0.6205			
	C5	28.621	18.893	9.728		0.3236	0.7991			
Model	M1	24.881	16.615	8.266		-1.1384	2.2014	1.563	0.4482	
	M2	27.27	18.745	8.525		-0.8794	1.8396			
	M3	27.809	18.906	8.903		-0.5014	1.4156			
	M4	26.728	17.512	9.216		-0.1884	1.1395			
	M5	24.512	15.393	9.119		-0.2854	1.2187			
MCC950	MC1	25.867	16.681	9.186		-0.2184	1.1634	1.446	0.4436	0.689798208
	MC2	26.928	18.379	8.549		-0.8554	1.8093			
	MC3	28.38	19.648	8.732		-0.6724	1.5937			
	MC4	28.153	19.633	8.52		-0.8844	1.846			
	MC5	27.544	17.851	9.693		0.2886	0.8187			

mRNA level of TLR4 in macrophages										
		TLR4	GAPDH							
Group	number	CT	CT	△CT	△CT Mean	△△CT	$2^{-\triangle\triangle CT}$	Mean	SD	Compared to Model
Control	C1	26.312	22.925	3.387	3.1784	0.2086	0.8654	1.006	0.1271	0.000685222
	C2	24.764	21.443	3.321		0.1426	0.9059			
	C3	24.814	21.62	3.194		0.0156	0.9892			
	C4	23.896	20.928	2.968		-0.2104	1.157			
	C5	25.364	22.342	3.022		-0.1564	1.1145			
Model	M1	25.201	22.9	2.301		-0.8774	1.8371	1.533	0.1794	
	M2	23.876	21.244	2.632		-0.5464	1.4604			
	M3	24.308	21.756	2.552		-0.6264	1.5437			
	M4	23.226	20.52	2.706		-0.4724	1.3874			
	M5	23.707	21.049	2.658		-0.5204	1.4344			
MCC950	MC1	25.83	23.023	2.807		-0.3714	1.2936	1.332	0.1784	0.113651912
	MC2	24.757	22.298	2.459		-0.7194	1.6465			
	MC3	24.921	22.083	2.838		-0.3404	1.2661			
	MC4	24.386	21.492	2.894		-0.2844	1.2179			
	MC5	25.296	22.421	2.875		-0.3034	1.234			

mRNA level of NLRP3 in macrophages										
		NLRP3	GAPDH							
Group	number	CT	CT	△CT	△CT Mean	△△CT	$2^{-\triangle\triangle CT}$	Mean	SD	Compared to Model
Control	C1	25.15	22.925	2.225	2.2858	-0.0608	1.043	1.002	0.0629	1.20963E-06
	C2	23.779	21.443	2.336		0.0502	0.9658			
	C3	23.817	21.62	2.197		-0.0888	1.0635			
	C4	23.35	20.928	2.422		0.1362	0.9099			
	C5	24.591	22.342	2.249		-0.0368	1.0258			
Model	M1	24.501	22.9	1.601		-0.6848	1.6075	1.54	0.0685	
	M2	22.943	21.244	1.699		-0.5868	1.5019			
	M3	23.416	21.756	1.66		-0.6258	1.5431			
	M4	22.127	20.52	1.607		-0.6788	1.6008			
	M5	22.804	21.049	1.755		-0.5308	1.4447			
MCC950	MC1	24.682	23.023	1.659		-0.6268	1.5441	1.623	0.0904	0.140016096
	MC2	23.844	22.298	1.546		-0.7398	1.6699			
	MC3	23.575	22.083	1.492		-0.7938	1.7336			
	MC4	23.177	21.492	1.685		-0.6008	1.5166			
	MC5	23.985	22.421	1.564		-0.7218	1.6492			

mRNA level of Caspase-1 in macrophages										
		Caspase-1	GAPDH							
Group	number	CT	CT	△CT	△CT Mean	△△CT	$2^{-\triangle\triangle CT}$	Mean	SD	Compared to Model
Control	C1	23.286	22.925	0.361	0.7484	-0.3874	1.308	1.012	0.1832	0.004469098
	C2	22.214	21.443	0.771		0.0226	0.9845			
	C3	22.49	21.62	0.87		0.1216	0.9192			
	C4	21.963	20.928	1.035		0.2866	0.8198			
	C5	23.047	22.342	0.705		-0.0434	1.0305			
Model	M1	23.151	22.9	0.251		-0.4974	1.4117	1.347	0.0549	
	M2	21.617	21.244	0.373		-0.3754	1.2972			
	M3	22.054	21.756	0.298		-0.4504	1.3664			
	M4	20.909	20.52	0.389		-0.3594	1.2829			
	M5	21.336	21.049	0.287		-0.4614	1.3769			
MCC950	MC1	22.706	23.023	-0.317		-1.0654	2.0928	1.772	0.5324	0.113803901
	MC2	22.489	22.298	0.191		-0.5574	1.4716			
	MC3	21.486	22.083	-0.597		-1.3454	2.541			
	MC4	21.643	21.492	0.151		-0.5974	1.513			
	MC5	22.858	22.421	0.437		-0.3114	1.2409			

**mRNA level of IL-1β in macrophages**										
		IL-1β	GAPDH							
Group	number	CT	CT	△CT	△CT Mean	△△CT	$2^{-\triangle\triangle CT}$	Mean	SD	Compared to Model
Control	C1	19.322	22.925	-3.603	-3.754	0.151	0.9006	1.004	0.0952	6.64592E-07
	C2	17.832	21.443	-3.611		0.143	0.9056			
	C3	17.781	21.62	-3.839		-0.085	1.0607			
	C4	17.116	20.928	-3.812		-0.058	1.041			
	C5	18.437	22.342	-3.905		-0.151	1.1103			
Model	M1	18.417	22.9	-4.483		-0.729	1.6575	1.674	0.0493	
	M2	16.816	21.244	-4.428		-0.674	1.5955			
	M3	17.22	21.756	-4.536		-0.782	1.7195			
	M4	16.005	20.52	-4.515		-0.761	1.6947			
	M5	16.528	21.049	-4.521		-0.767	1.7017			
MCC950	MC1	18.323	23.023	-4.7		-0.946	1.9265	1.701	0.1375	0.684793167
	MC2	17.879	22.298	-4.419		-0.665	1.5856			
	MC3	17.593	22.083	-4.49		-0.736	1.6656			
	MC4	16.951	21.492	-4.541		-0.787	1.7255			
	MC5	17.986	22.421	-4.435		-0.681	1.6033			
**mRNA level of IL-18 in macrophages**										
		IL-18	GAPDH							
Group	number	CT	CT	△CT	△CT Mean	△△CT	$2^{-\triangle\triangle CT}$	Mean	SD	Compared to Model
Control	C1	25.699	22.925	2.774	2.6226	0.1514	0.9004	1.004	0.0989	0.000150999
	C2	24.12	21.443	2.677		0.0544	0.963			
	C3	24.328	21.62	2.708		0.0854	0.9425			
	C4	23.445	20.928	2.517		-0.1056	1.0759			
	C5	24.779	22.342	2.437		-0.1856	1.1373			
Model	M1	25.173	22.9	2.273		-0.3496	1.2742	1.39	0.0821	
	M2	23.406	21.244	2.162		-0.4606	1.3761			
	M3	23.884	21.756	2.128		-0.4946	1.4089			
	M4	22.673	20.52	2.153		-0.4696	1.3847			
	M5	23.083	21.049	2.034		-0.5886	1.5038			
MCC950	MC1	25.372	23.023	2.349		-0.2736	1.2088	1.28	0.1336	0.157560545
	MC2	24.585	22.298	2.287		-0.3356	1.2619			
	MC3	24.122	22.083	2.039		-0.5836	1.4986			
	MC4	23.751	21.492	2.259		-0.3636	1.2866			
	MC5	24.848	22.421	2.427		-0.1956	1.1452			
**mRNA level of GSDMD in macrophages**										
		GSDMD	GAPDH							
Group	number	CT	CT	△CT	△CT Mean	△△CT	$2^{-\triangle\triangle CT}$	Mean	SD	Compared to Model
Control	C1	26.185	22.925	3.26	3.3098	-0.0498	1.0351	1.002	0.0771	0.19799753
	C2	24.939	21.443	3.496		0.1862	0.8789			
	C3	24.886	21.62	3.266		-0.0438	1.0308			
	C4	24.259	20.928	3.331		0.0212	0.9854			
	C5	25.538	22.342	3.196		-0.1138	1.0821			
Model	M1	25.938	22.9	3.038		-0.2718	1.2073	1.074	0.0836	
	M2	24.461	21.244	3.217		-0.0928	1.0664			
	M3	25.091	21.756	3.335		0.0252	0.9827			
	M4	23.784	20.52	3.264		-0.0458	1.0323			
	M5	24.247	21.049	3.198		-0.1118	1.0806			
MCC950	MC1	26.394	23.023	3.371		0.0612	0.9585	1.14	0.2974	0.645627377
	MC2	25.458	22.298	3.16		-0.1498	1.1094			
	MC3	25.696	22.083	3.613		0.3032	0.8105			
	MC4	24.503	21.492	3.011		-0.2988	1.2301			
	MC5	25.061	22.421	2.64		-0.6698	1.5909			

**mRNA level of TNF-α in macrophages**										
		TNF-α	GAPDH							
Group	number	CT	CT	△CT	△CT Mean	△△CT	$2^{-\triangle\triangle CT}$	Mean	SD	Compared to Model
Control	C1	25.837	22.925	2.912	2.672	0.24	0.8467	1.012	0.1823	0.155606346
	C2	24.108	21.443	2.665		-0.007	1.0049			
	C3	23.899	21.62	2.279		-0.393	1.3131			
	C4	23.592	20.928	2.664		-0.008	1.0056			
	C5	25.182	22.342	2.84		0.168	0.8901			
Model	M1	25.22	22.9	2.32		-0.352	1.2763	1.214	0.2231	
	M2	23.63	21.244	2.386		-0.286	1.2193			
	M3	23.819	21.756	2.063		-0.609	1.5252			
	M4	23.329	20.52	2.809		0.137	0.9094			
	M5	23.532	21.049	2.483		-0.189	1.14			
MCC950	MC1	25.489	23.023	2.466		-0.206	1.1535	1.501	0.3064	0.128772863
	MC2	24.072	22.298	1.774		-0.898	1.8635			
	MC3	23.946	22.083	1.863		-0.809	1.752			
	MC4	23.598	21.492	2.106		-0.566	1.4804			
	MC5	24.764	22.421	2.343		-0.329	1.2561			
**mRNA level of Caspase-3 in macrophages**										
		Caspase-3	GAPDH							
Group	number	CT	CT	△CT	△CT Mean	△△CT	$2^{-\triangle\triangle CT}$	Mean	SD	Compared to Model
Control	C1	23.683	22.925	0.758	0.855	-0.097	1.0695	1.01	0.1593	0.05885701
	C2	22.144	21.443	0.701		-0.154	1.1127			
	C3	22.791	21.62	1.171		0.316	0.8033			
	C4	21.958	20.928	1.03		0.175	0.8858			
	C5	22.957	22.342	0.615		-0.24	1.181			
Model	M1	24.19	22.9	1.29		0.435	0.7397	0.809	0.1278	
	M2	22.673	21.244	1.429		0.574	0.6718			
	M3	22.613	21.756	0.857		0.002	0.9986			
	M4	21.76	20.52	1.24		0.385	0.7658			
	M5	22.103	21.049	1.054		0.199	0.8712			
MCC950	MC1	23.982	23.023	0.959		0.104	0.9304	0.977	0.1714	0.117298488
	MC2	22.849	22.298	0.551		-0.304	1.2346			
	MC3	22.868	22.083	0.785		-0.07	1.0497			
	MC4	22.682	21.492	1.19		0.335	0.7928			
	MC5	23.463	22.421	1.042		0.187	0.8784			
**mRNA level of Caspase-4 in macrophages**										
		Caspase-4	GAPDH							
Group	number	CT	CT	△CT	△CT Mean	△△CT	$2^{-\triangle\triangle CT}$	Mean	SD	Compared to Model
Control	C1	22.537	22.925	-0.388	-0.2198	-0.1682	1.1237	1.003	0.0845	0.113366239
	C2	21.194	21.443	-0.249		-0.0292	1.0204			
	C3	21.571	21.62	-0.049		0.1708	0.8883			
	C4	20.705	20.928	-0.223		-0.0032	1.0022			
	C5	22.152	22.342	-0.19		0.0298	0.9796			
Model	M1	22.002	22.9	-0.898		-0.6782	1.6001	1.24	0.2857	
	M2	20.987	21.244	-0.257		-0.0372	1.0261			
	M3	21.285	21.756	-0.471		-0.2512	1.1902			
	M4	20.416	20.52	-0.104		0.1158	0.9229			
	M5	20.284	21.049	-0.765		-0.5452	1.4592			
MCC950	MC1	22.542	23.023	-0.481		-0.2612	1.1985	1.257	0.1229	0.902215633
	MC2	21.602	22.298	-0.696		-0.4762	1.3911			
	MC3	21.732	22.083	-0.351		-0.1312	1.0952			
	MC4	20.969	21.492	-0.523		-0.3032	1.2339			
	MC5	21.749	22.421	-0.672		-0.4522	1.3681			

**mRNA level of Caspase-5 in macrophages**										
		Caspase-5	GAPDH							
Group	number	CT	CT	△CT	△CT Mean	△△CT	$2^{-\triangle\triangle CT}$	Mean	SD	Compared to Model
Control	C1	23.562	22.925	0.637	0.3638	0.2732	0.8275	1.005	0.1099	0.550273667
	C2	21.846	21.443	0.403		0.0392	0.9732			
	C3	21.916	21.62	0.296		-0.0678	1.0481			
	C4	21.169	20.928	0.241		-0.1228	1.0888			
	C5	22.584	22.342	0.242		-0.1218	1.0881			
Model	M1	23.477	22.9	0.577		0.2132	0.8626	0.935	0.2252	
	M2	22.338	21.244	1.094		0.7302	0.6028			
	M3	21.86	21.756	0.104		-0.2598	1.1973			
	M4	20.969	20.52	0.449		0.0852	0.9427			
	M5	21.314	21.049	0.265		-0.0988	1.0709			
MCC950	MC1	23.033	23.023	0.01		-0.3538	1.2779	1.076	0.2545	0.382970219
	MC2	22.761	22.298	0.463		0.0992	0.9336			
	MC3	21.971	22.083	-0.112		-0.4758	1.3907			
	MC4	22.234	21.492	0.742		0.3782	0.7694			
	MC5	22.776	22.421	0.355		-0.0088	1.0061			
**mRNA level of NLRP1 in macrophages**										
		NLRP1	GAPDH							
Group	number	CT	CT	△CT	△CT Mean	△△CT	$2^{-\triangle\triangle CT}$	Mean	SD	Compared to Model
Control	C1	25.807	22.925	2.882	3.1518	-0.2698	1.2056	1.016	0.1983	0.071084337
	C2	24.567	21.443	3.124		-0.0278	1.0195			
	C3	24.974	21.62	3.354		0.2022	0.8692			
	C4	24.456	20.928	3.528		0.3762	0.7705			
	C5	25.213	22.342	2.871		-0.2808	1.2149			
Model	M1	26.666	22.9	3.766		0.6142	0.6533	0.793	0.1343	
	M2	24.424	21.244	3.18		0.0282	0.9806			
	M3	25.248	21.756	3.492		0.3402	0.7899			
	M4	23.887	20.52	3.367		0.2152	0.8614			
	M5	24.757	21.049	3.708		0.5562	0.6801			
MCC950	MC1	26.079	23.023	3.056		-0.0958	1.0687	0.993	0.2656	0.172028165
	MC2	25.735	22.298	3.437		0.2852	0.8206			
	MC3	25.779	22.083	3.696		0.5442	0.6858			
	MC4	24.176	21.492	2.684		-0.4678	1.383			
	MC5	25.565	22.421	3.144		-0.0078	1.0054			
**mRNA level of NLRC4 in macrophages**										
		NLRC4	GAPDH							
Group	number	CT	CT	△CT	△CT Mean	△△CT	$2^{-\triangle\triangle CT}$	Mean	SD	Compared to Model
Control	C1	27.412	22.925	4.487	4.138	0.349	0.7851	1.024	0.2459	0.433049398
	C2	25.16	21.443	3.717		-0.421	1.3389			
	C3	25.587	21.62	3.967		-0.171	1.1258			
	C4	25.457	20.928	4.529		0.391	0.7626			
	C5	26.332	22.342	3.99		-0.148	1.108			
Model	M1	26.464	22.9	3.564		-0.574	1.4886	1.16	0.2731	
	M2	25.311	21.244	4.067		-0.071	1.0504			
	M3	25.525	21.756	3.769		-0.369	1.2915			
	M4	25.051	20.52	4.531		0.393	0.7615			
	M5	24.916	21.049	3.867		-0.271	1.2066			
MCC950	MC1	26.739	23.023	3.716		-0.422	1.3398	0.896	0.2786	0.168765848
	MC2	26.901	22.298	4.603		0.465	0.7245			
	MC3	26.872	22.083	4.789		0.651	0.6368			
	MC4	25.957	21.492	4.465		0.327	0.7972			
	MC5	26.587	22.421	4.166		0.028	0.9808			

**mRNA level of AIM2 in macrophages**										
		AIM2	GAPDH							
Group	number	CT	CT	△CT	△CT Mean	△△CT	$2^{-△△CT}$	Mean	SD	Compared to Model
Control	C1	27.698	22.925	4.773	4.7902	-0.0172	1.012	1.011	0.162	0.0854849
	C2	26.146	21.443	4.703		-0.0872	1.0623			
	C3	26.136	21.62	4.516		-0.2742	1.2093			
	C4	26.114	20.928	5.186		0.3958	0.7601			
	C5	27.115	22.342	4.773		-0.0172	1.012			
Model	M1	28.009	22.9	5.109		0.3188	0.8017	1.298	0.2836	
	M2	25.459	21.244	4.215		-0.5752	1.4899			
	M3	26.019	21.756	4.263		-0.5272	1.4411			
	M4	24.794	20.52	4.274		-0.5162	1.4302			
	M5	25.433	21.049	4.384		-0.4062	1.3252			
MCC950	MC1	28.324	23.023	5.301		0.5108	0.7018	1.015	0.1985	0.105649418
	MC2	27.064	22.298	4.766		-0.0242	1.0169			
	MC3	26.809	22.083	4.726		-0.0642	1.0455			
	MC4	25.957	21.492	4.465		-0.3252	1.2528			
	MC5	27.128	22.421	4.707		-0.0832	1.0594			

The following table shows the antibodies used in this experiment.

	Name	Company	Catalogue number	Molecular weight	Dilution rate
Primary antibodies	ASC	Santa Cruz	sc-514414	Monomer 24KDa<br>Dimer 48KDa	WB 1:500
	Caspase-1	Santa Cruz	sc-56036	Full length 45KDa<br>Cleaved 20KDa	WB 1:250
	GAPDH	ABCAM	ab181602	36KDa	WB 1:10000
	GSDMD	Santa Cruz	sc-393581	Full length 53KDa<br>GSDMD-N 30KDa	WB 1:250<br>IF 1:50
	IL-1β	ABCAM	ab9722	Full length 30KDa<br>Cleaved 17KDa	WB 1:500
	IL-18	Invitrogen	PA5-76082	Full length 24KDa<br>Cleaved 18KDa	WB 1:1000
	MOMA2	Santa Cruz	sc-59332	-	IF 1:100
	NLRP3	Invitrogen	PA5-115661	114KDa	WB 1:500<br>IF 1:100
	TLR4	Santa Cruz	sc-293072	95/120KDa	1:500
Secondary	Anti-mouse IgG,<br>HRP-linked Antibody	CST	#7076	-	1:1000
	Anti-rabbit IgG,<br>HRP-linked Antibody	ABCAM	Ab621	-	1:10000

The prestained protein Ladder was purchased from Thermo Fisher SCIENTIFIC (Catalogue number: 26616).

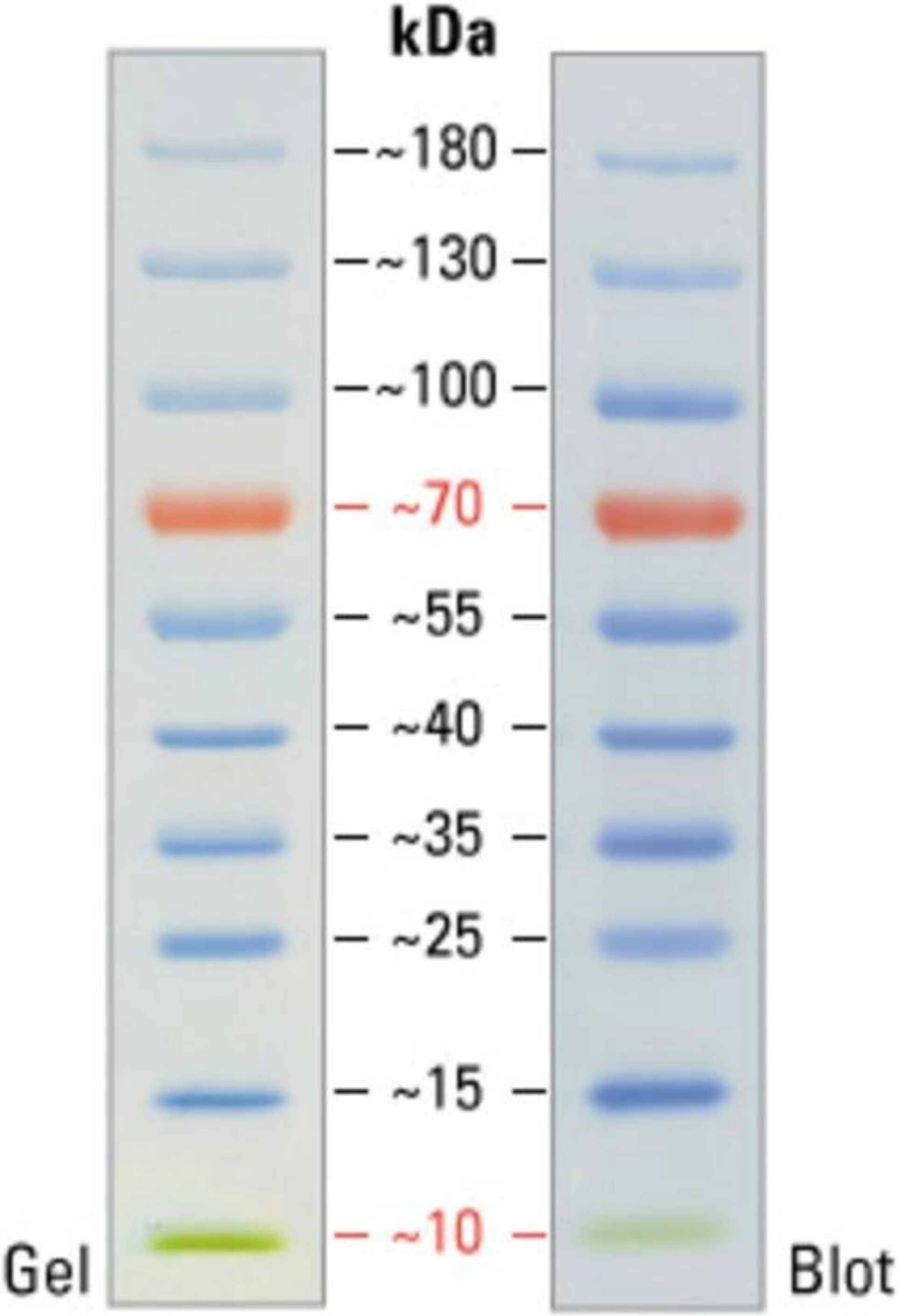

The protein samples for Western blot were divided into Control group (C1~C5, n=5), Model group (M1~M5, n=5) and MCC950 group (MC1~MC5, n=5).

The protein bands of TLR4 in aortas

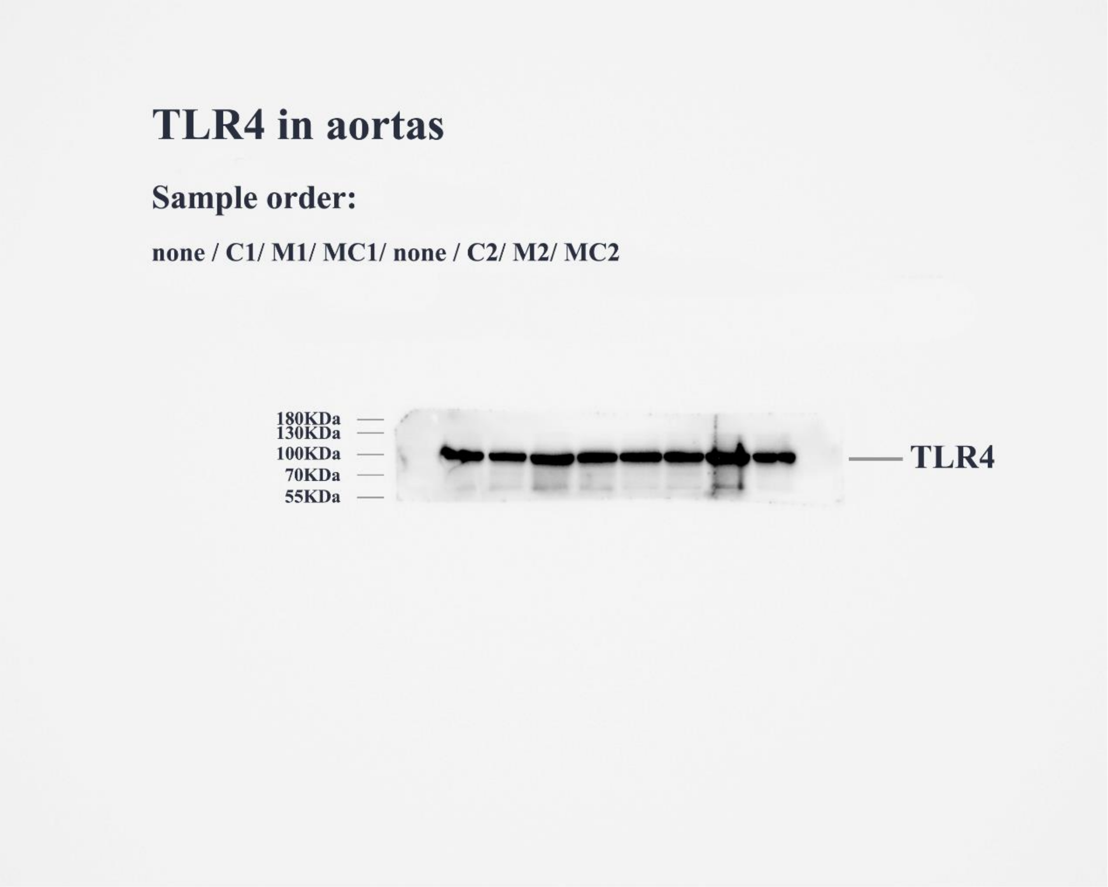

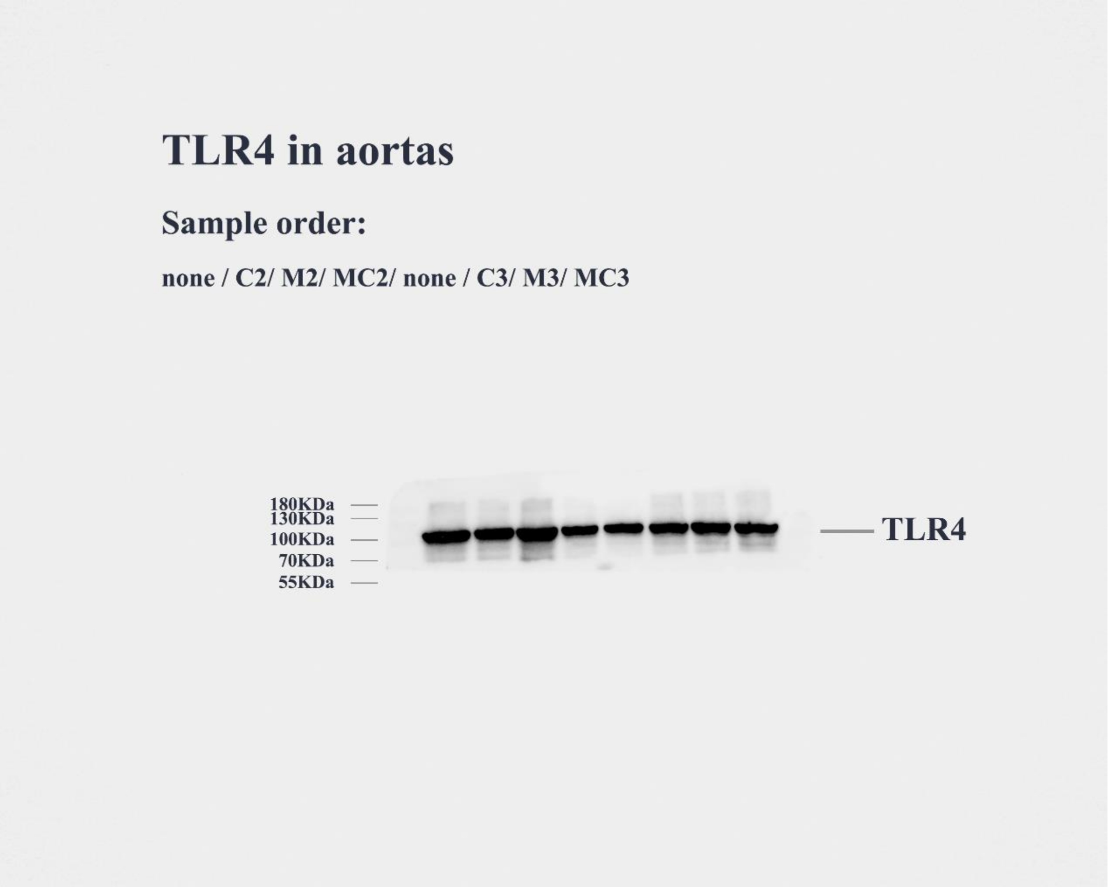

TLR4 in aortas

Sample order:

none / C3/ M3/ MC3/ none / C4/ M4/ MC4

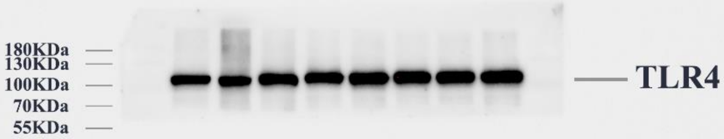

TLR4 in aortas

Sample order:

none / C4/ M4/ MC4/ none / C5/ M5/ MC5

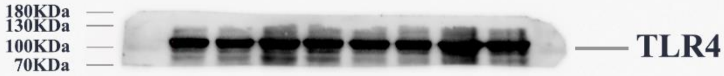

The protein bands of TLR4 in macrophages

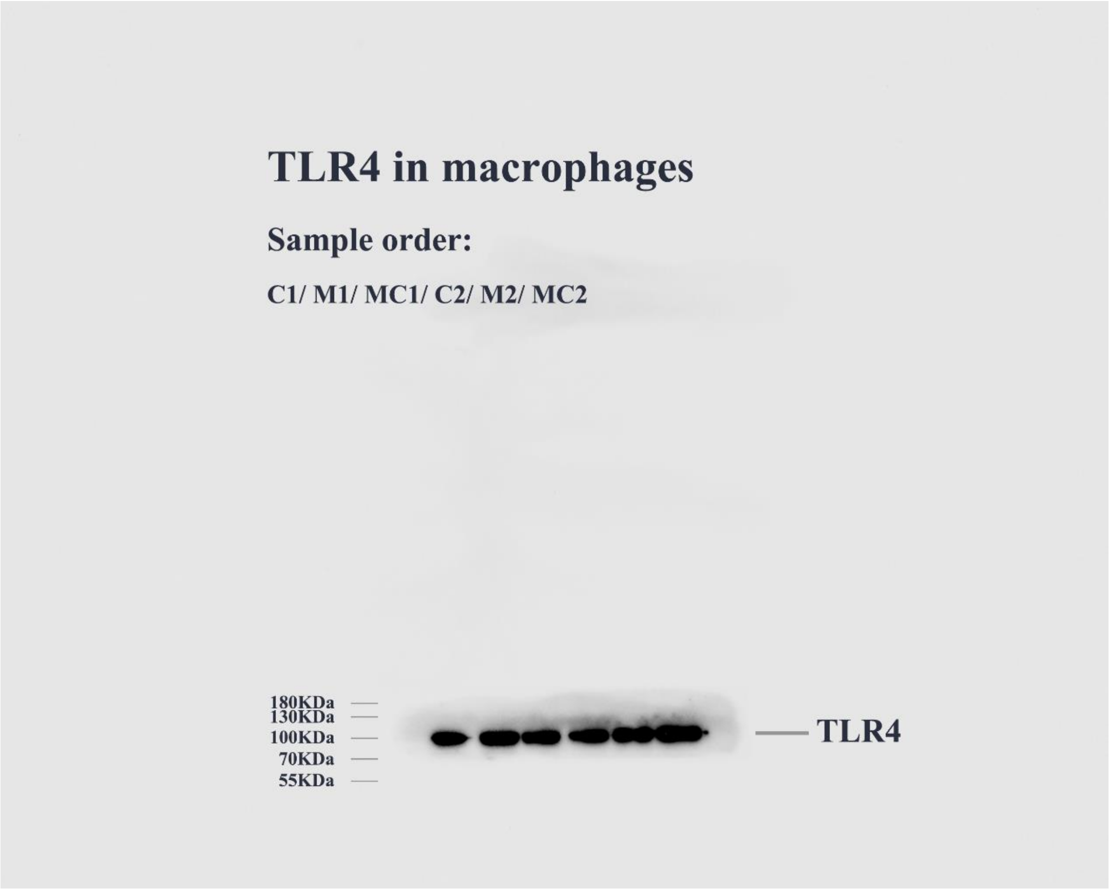

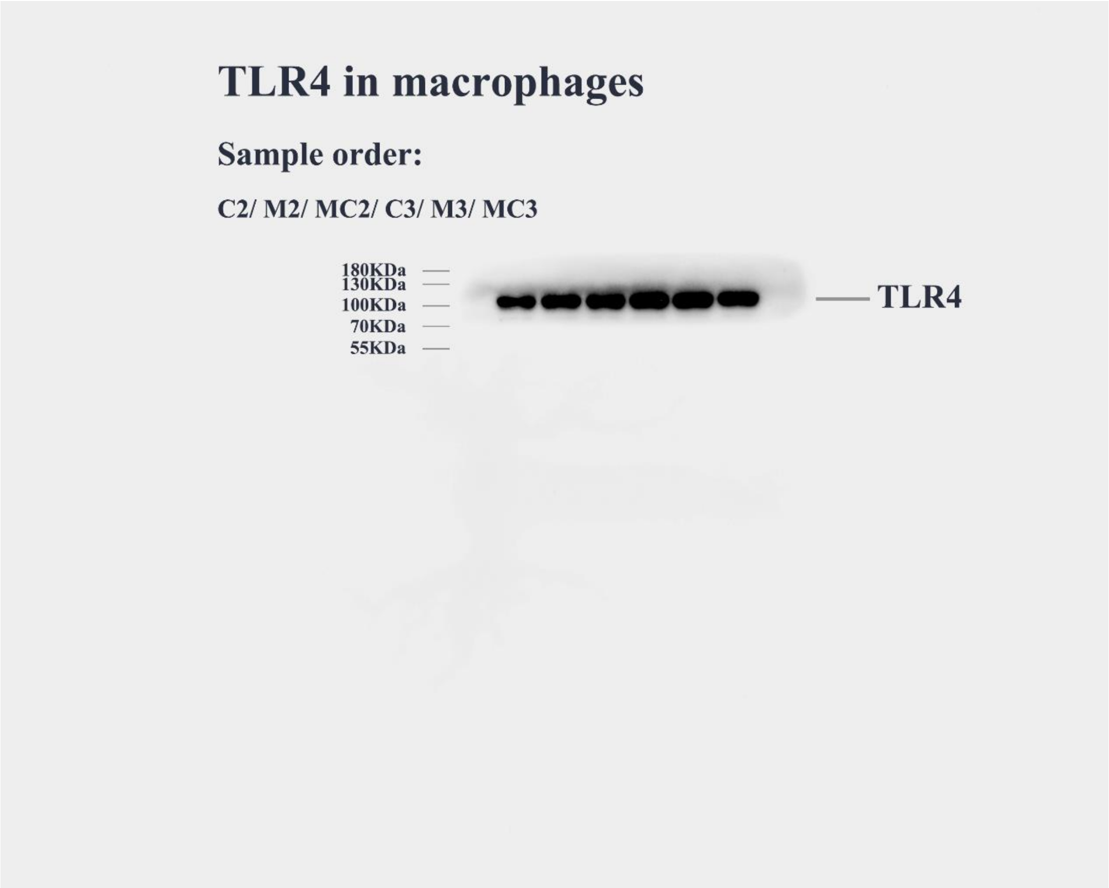

TLR4 in macrophages

Sample order:

C4/ M4/ MC4/ C5/ M5/ MC5

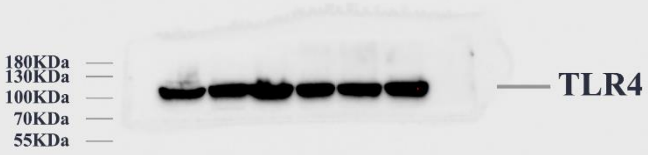

The protein bands of NLRP3 in aortas

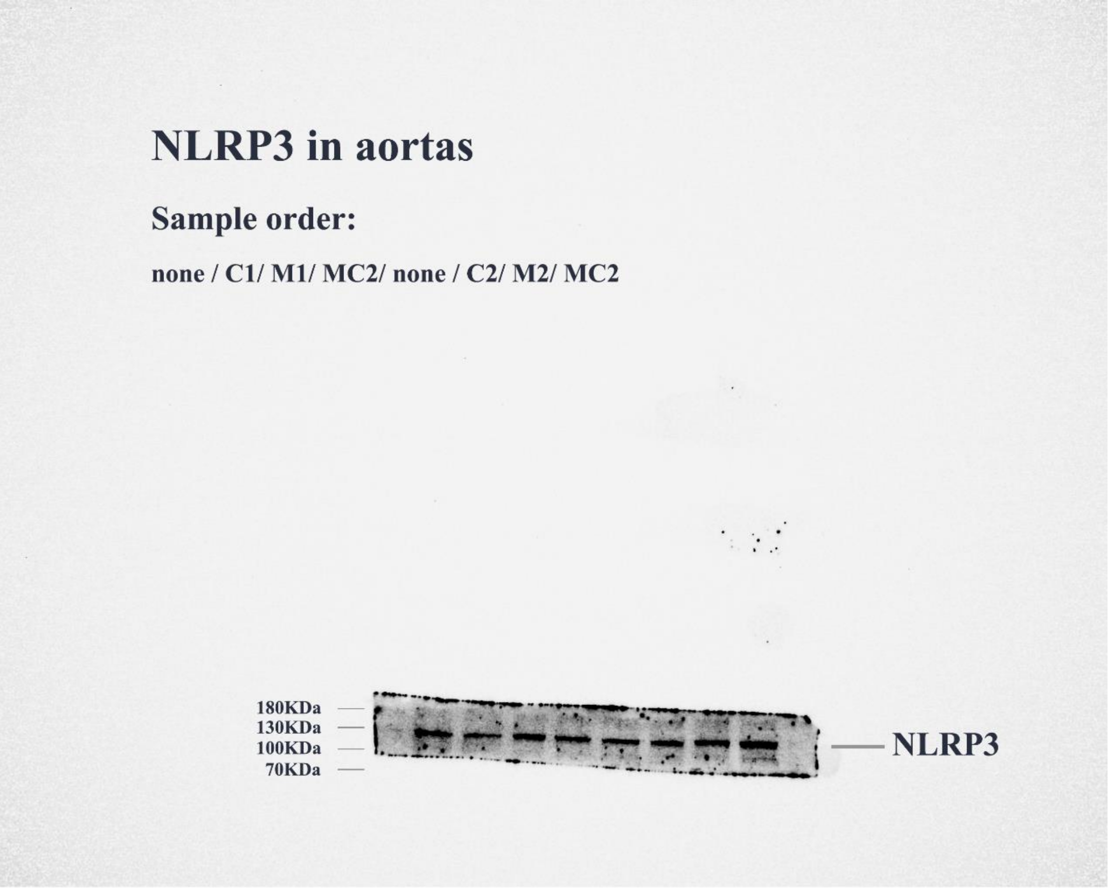

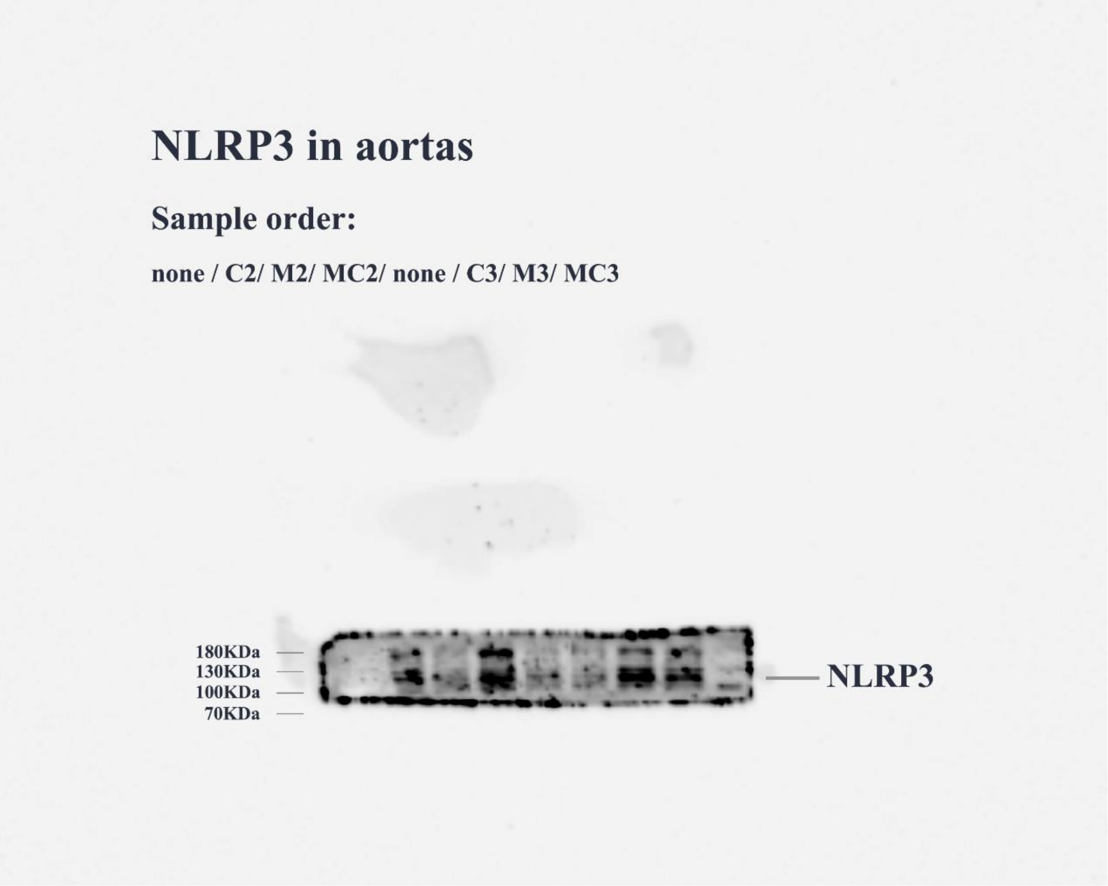

NLRP3

Sample order:

none / C3/ M3/ MC3/ none / C4/ M4/ MC4

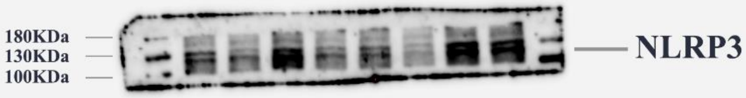

NLRP3 in aortas

Sample order:

none / C4/ M4/ MC4/ none / C5/ M5/ MC5

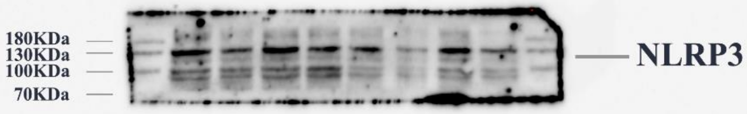

The protein bands of NLRP3 in macrophages

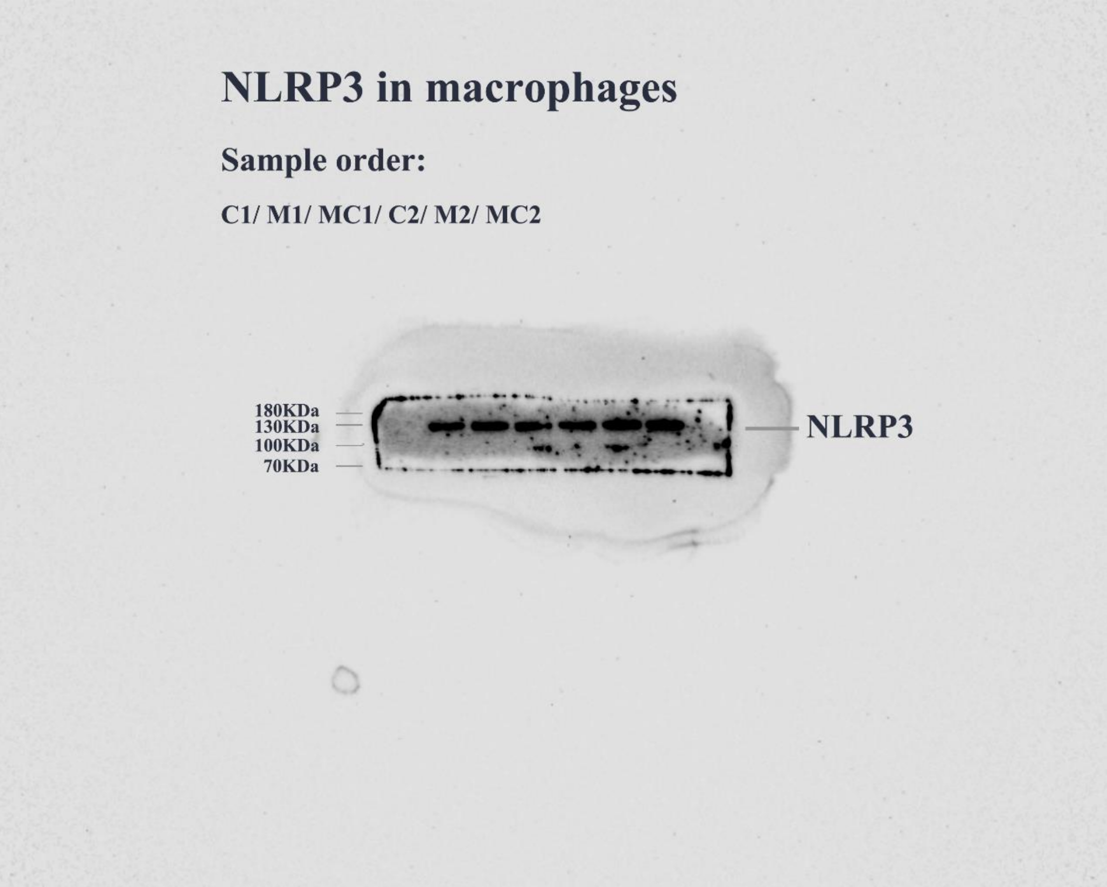

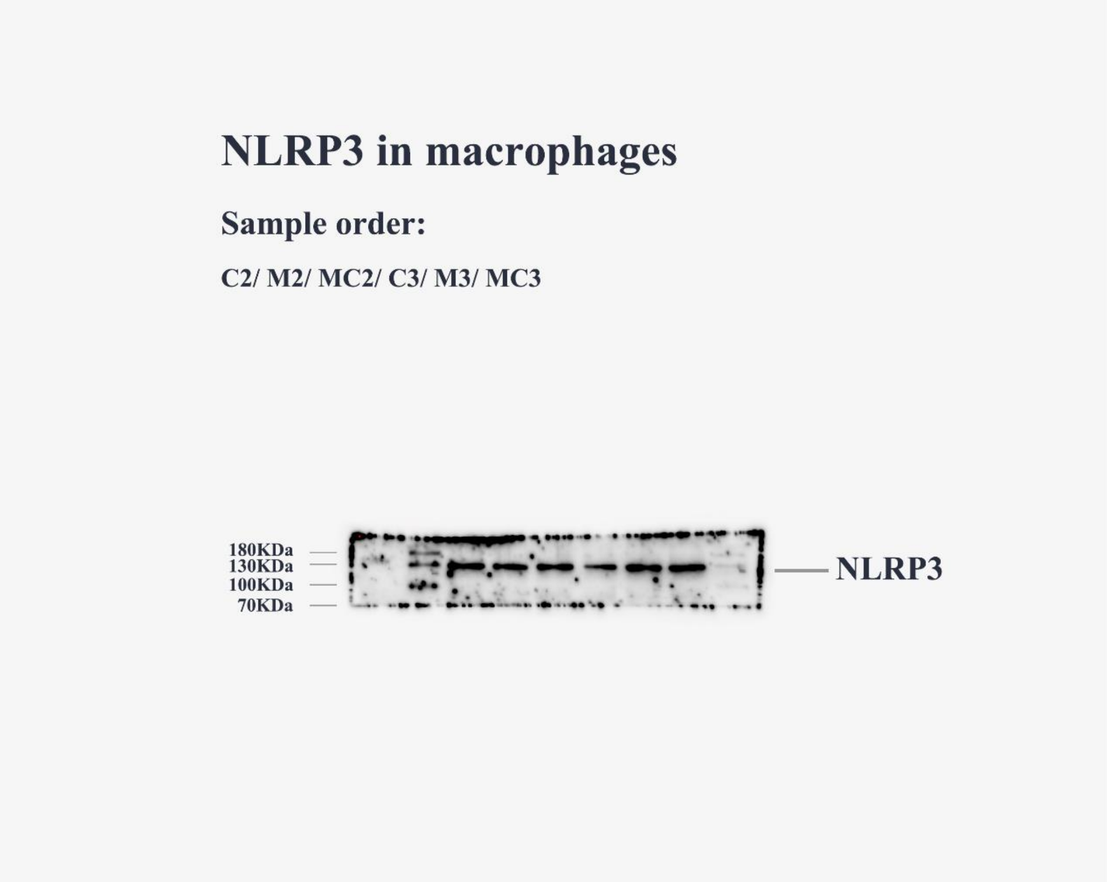

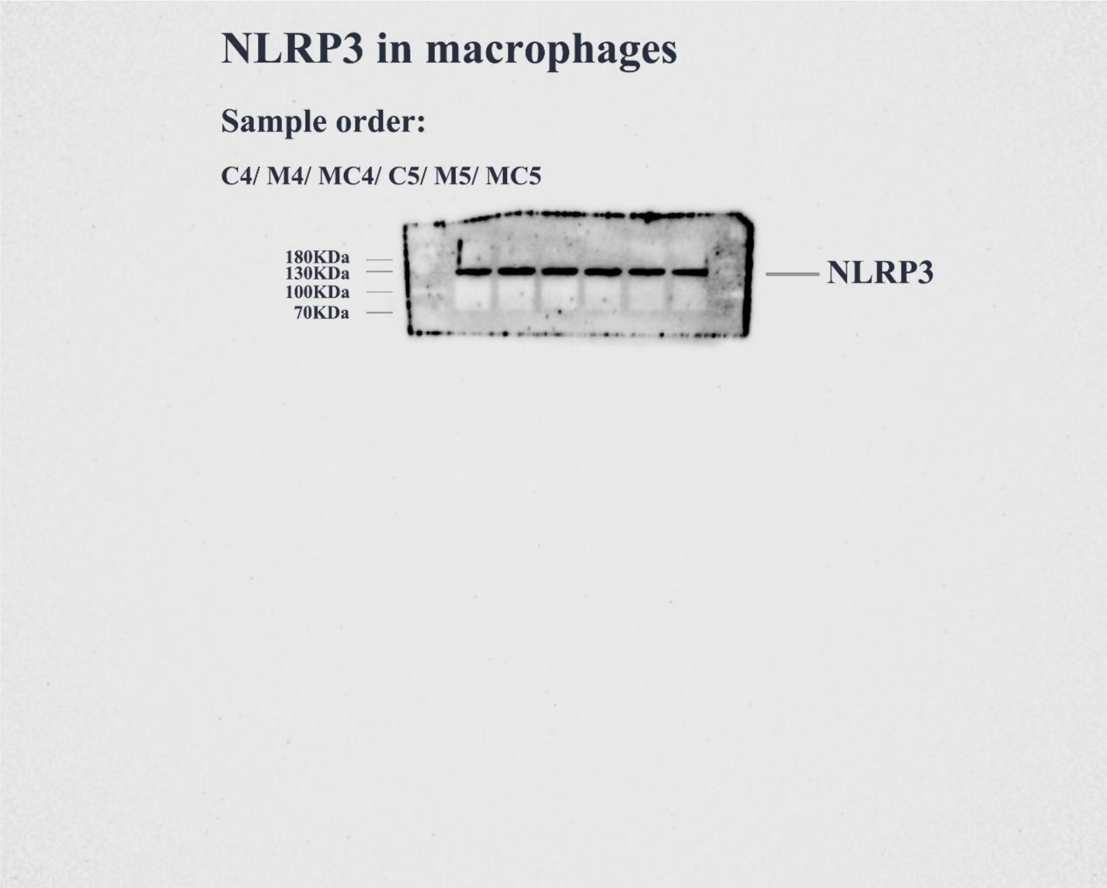

NLRP3 in macrophages
Sample order:
C4/ M4/ MC4/ C5/ M5/ MC5
180KDa
130KDa
100KDa
70KDa
NLRP3

The protein bands of ASC monomer in aortas

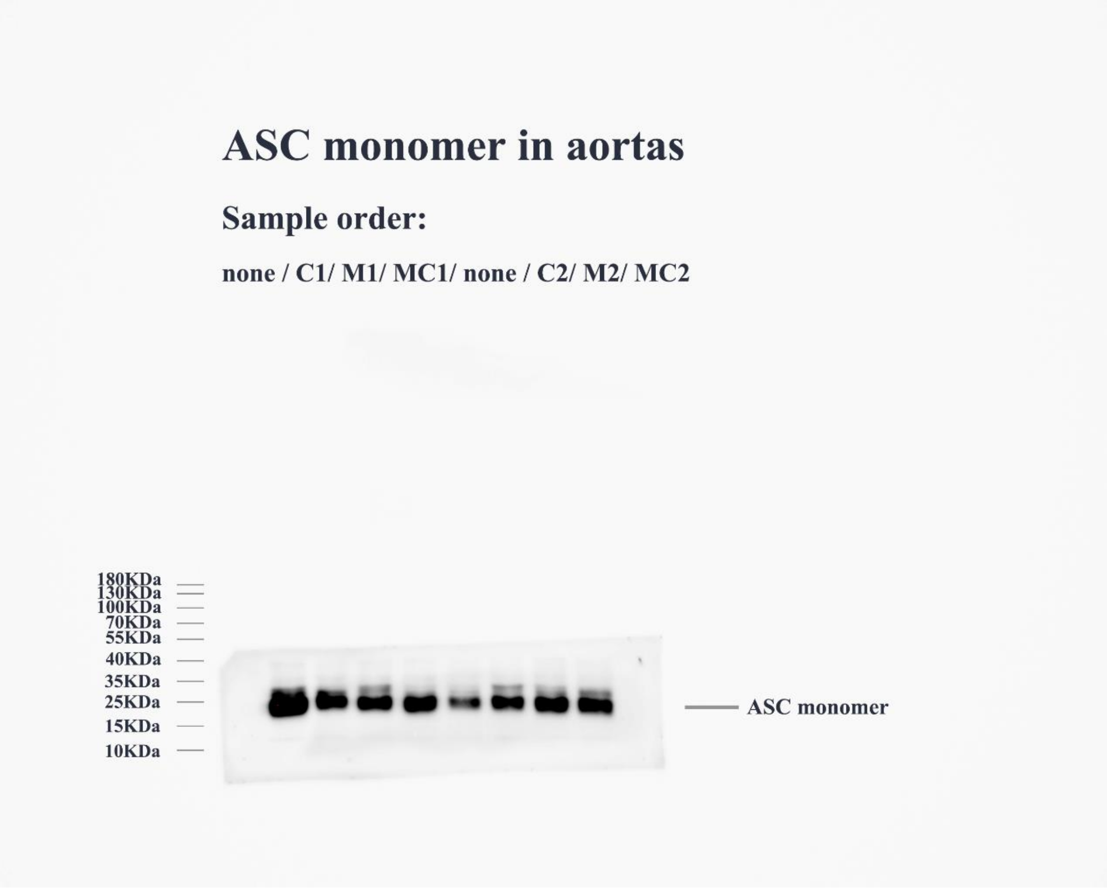

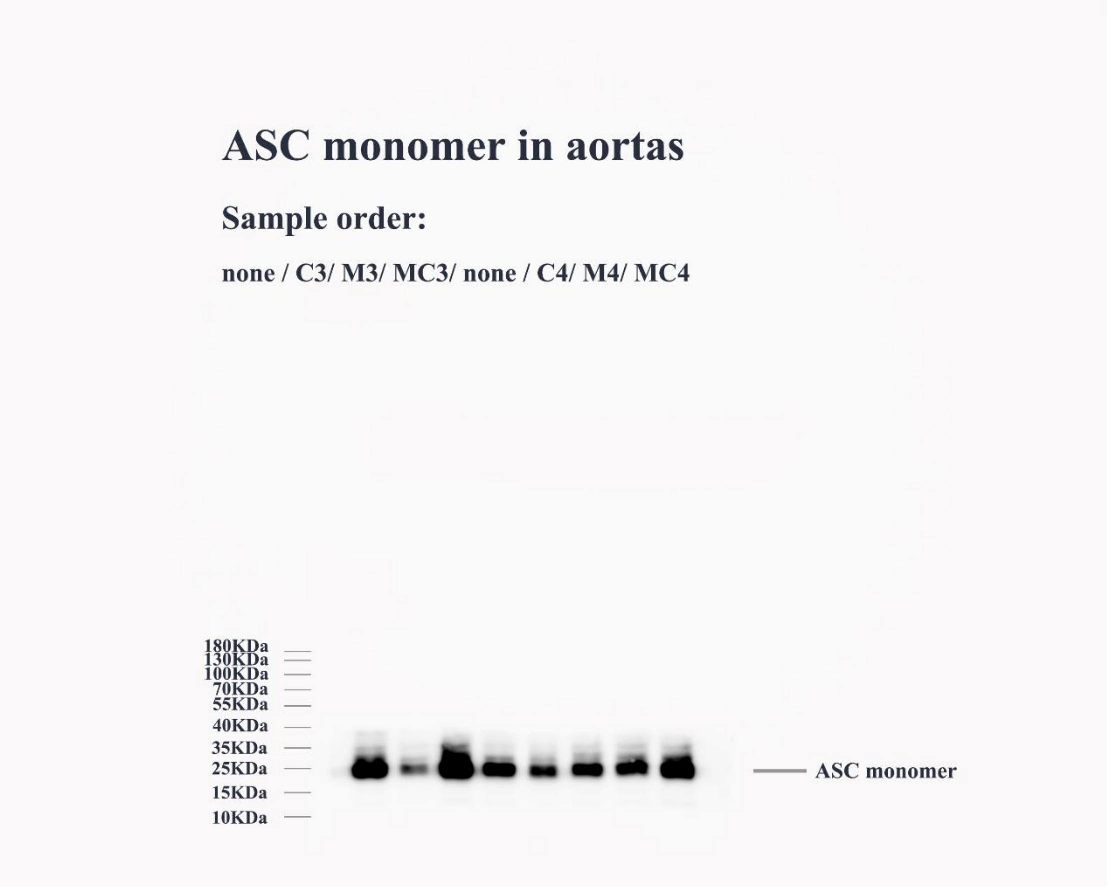

ASC monomer in aortas

Sample order:

none / C4/ M4/ MC4/ none / C5/ M5/ MC5

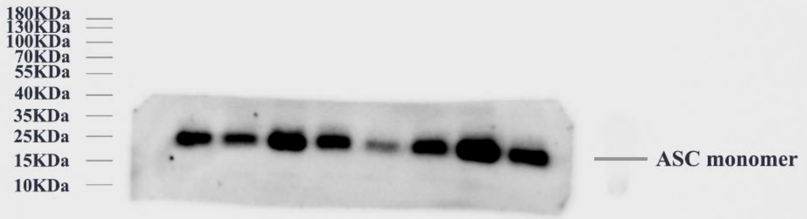

The protein bands of ASC oligomers in aortas

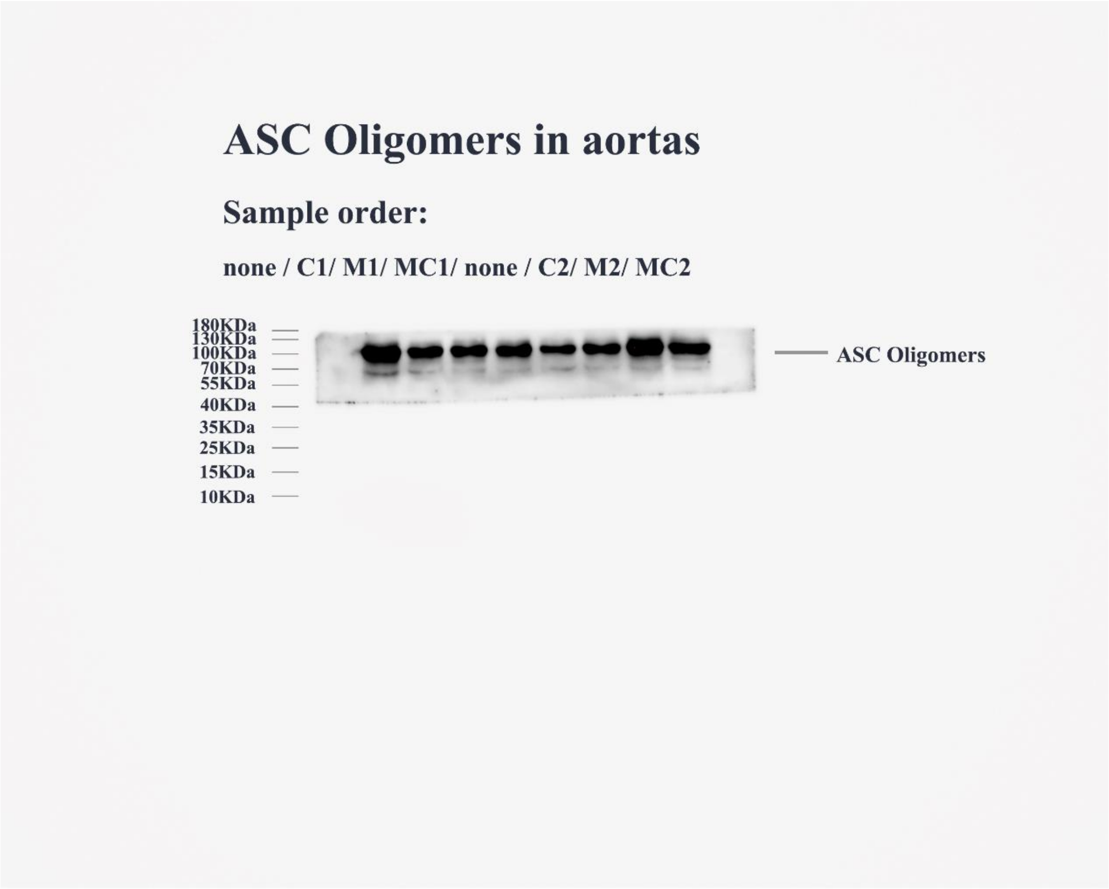

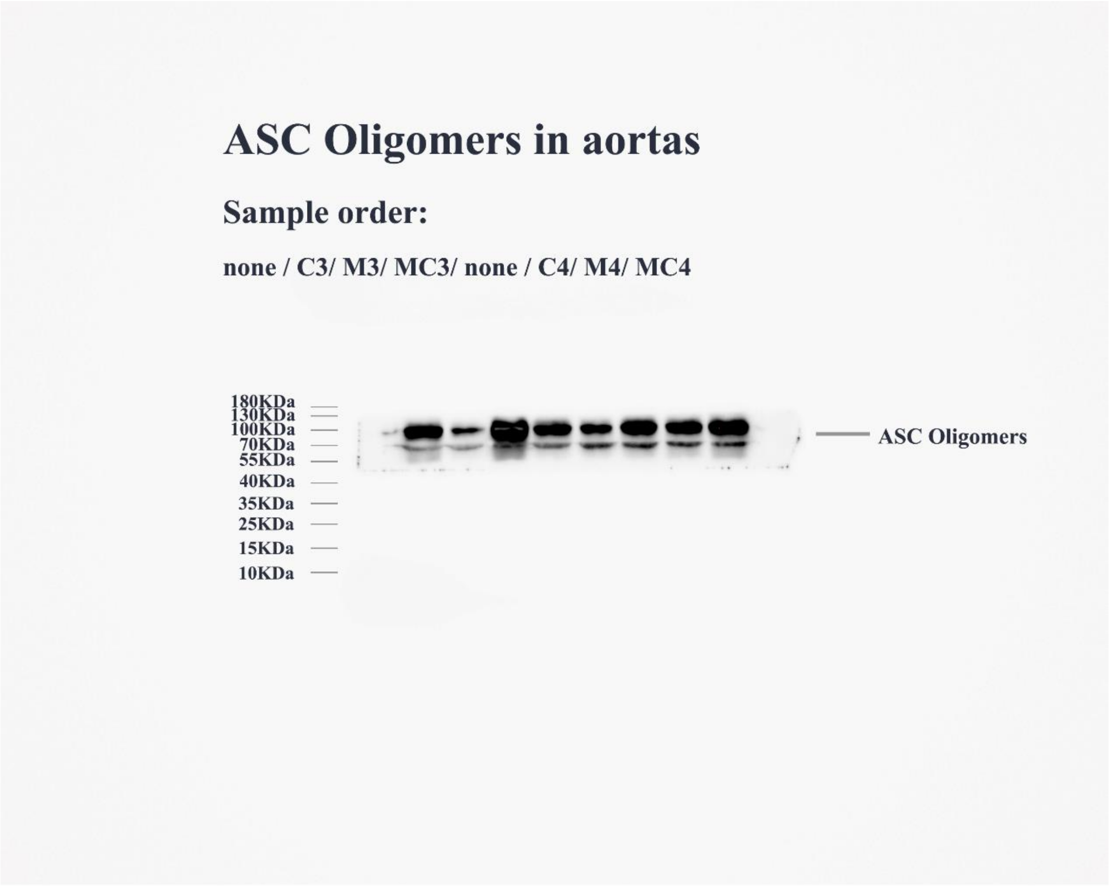

ASC Oligomers in aortas

Sample order:

none / C4/ M4/ MC4/ none / C5/ M5/ MC5

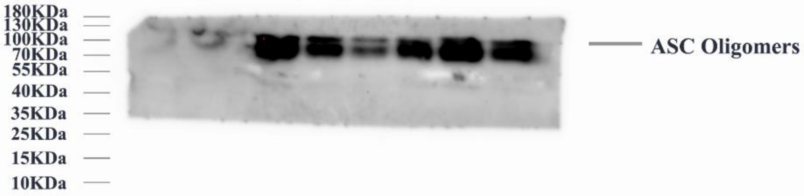

The protein bands of ASC in macrophages

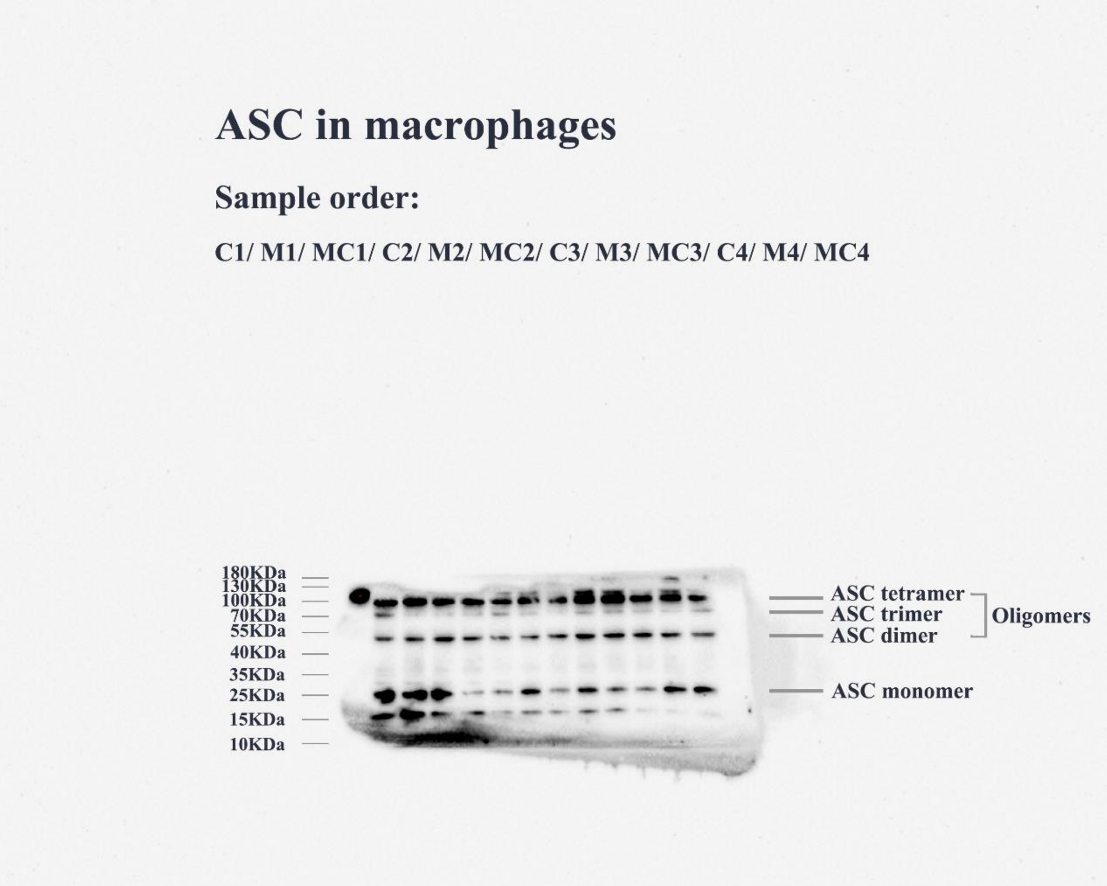

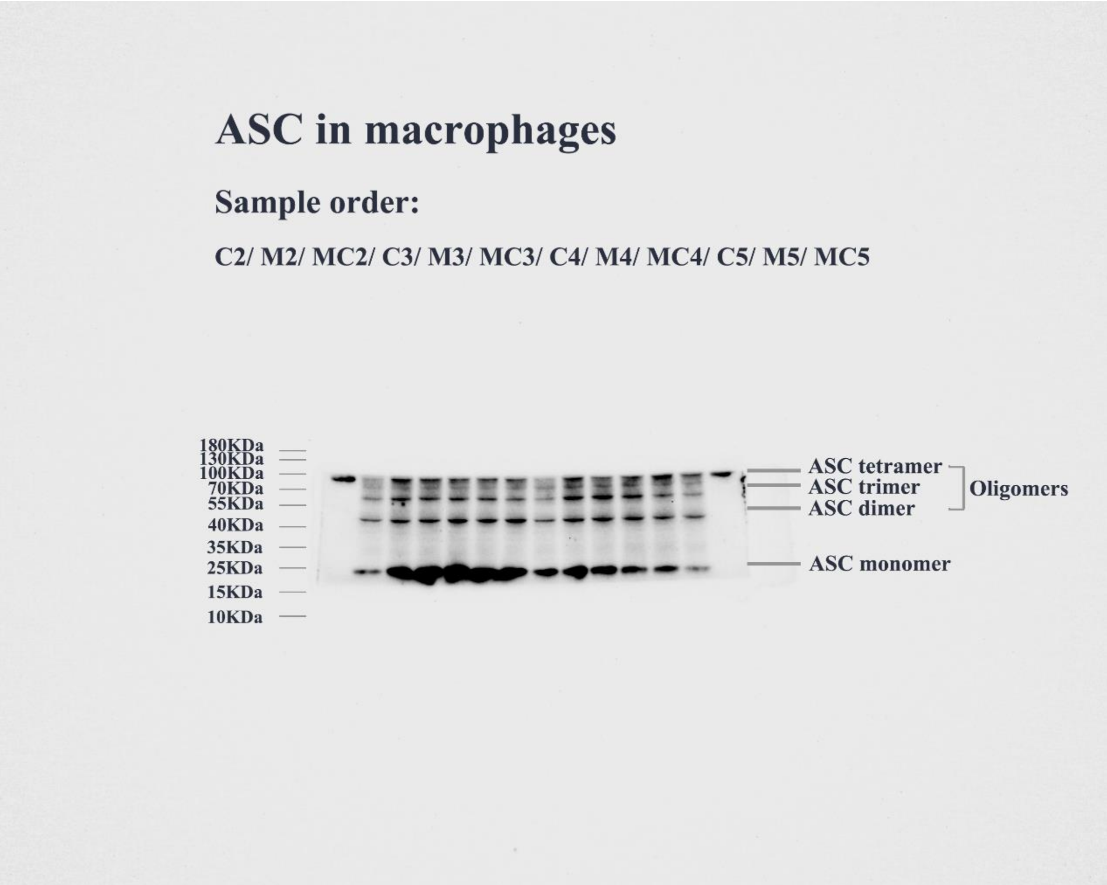

The protein bands of pro-Caspase1 in aortas

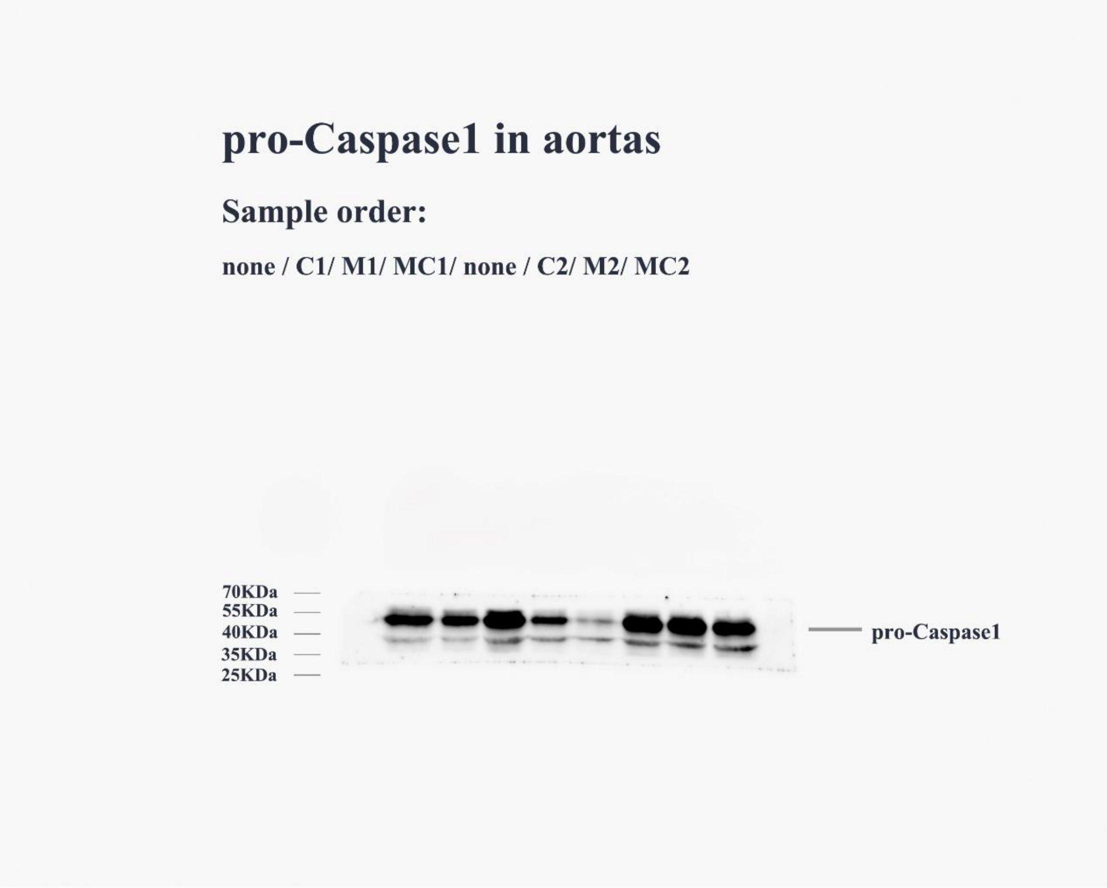

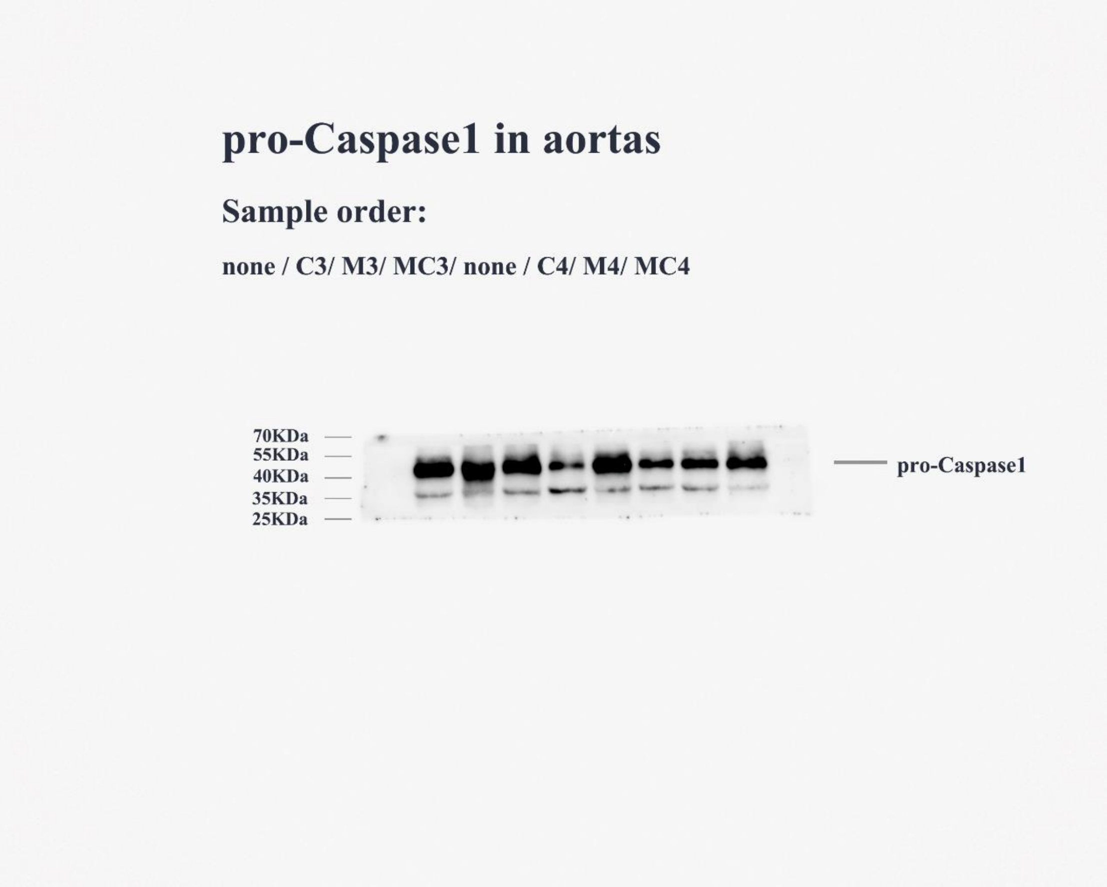

pro-Caspase1 in aortas

Sample order:

none / C4/ M4/ MC4/ none / C5/ M5/ MC5

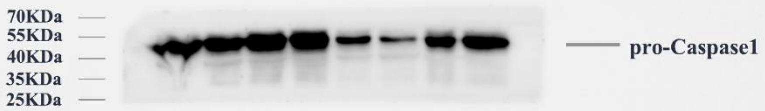

The protein bands of Caspase1 in aortas

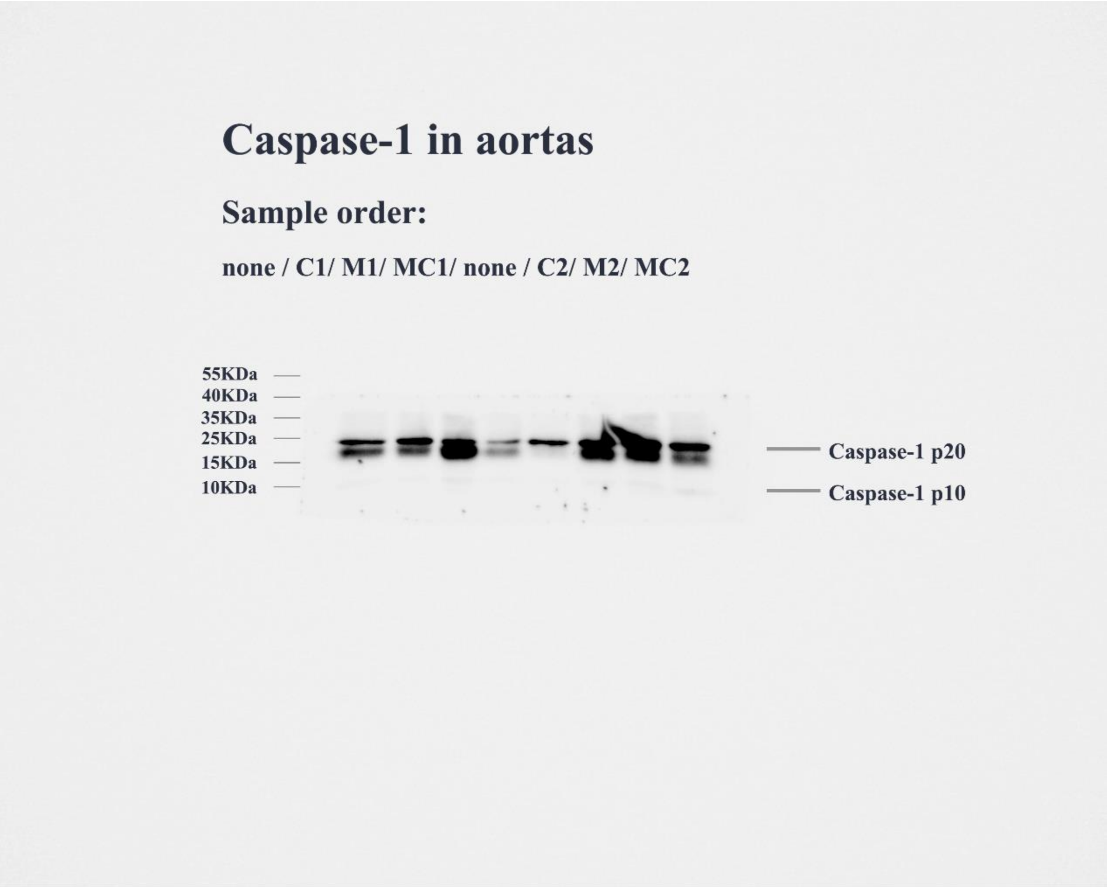

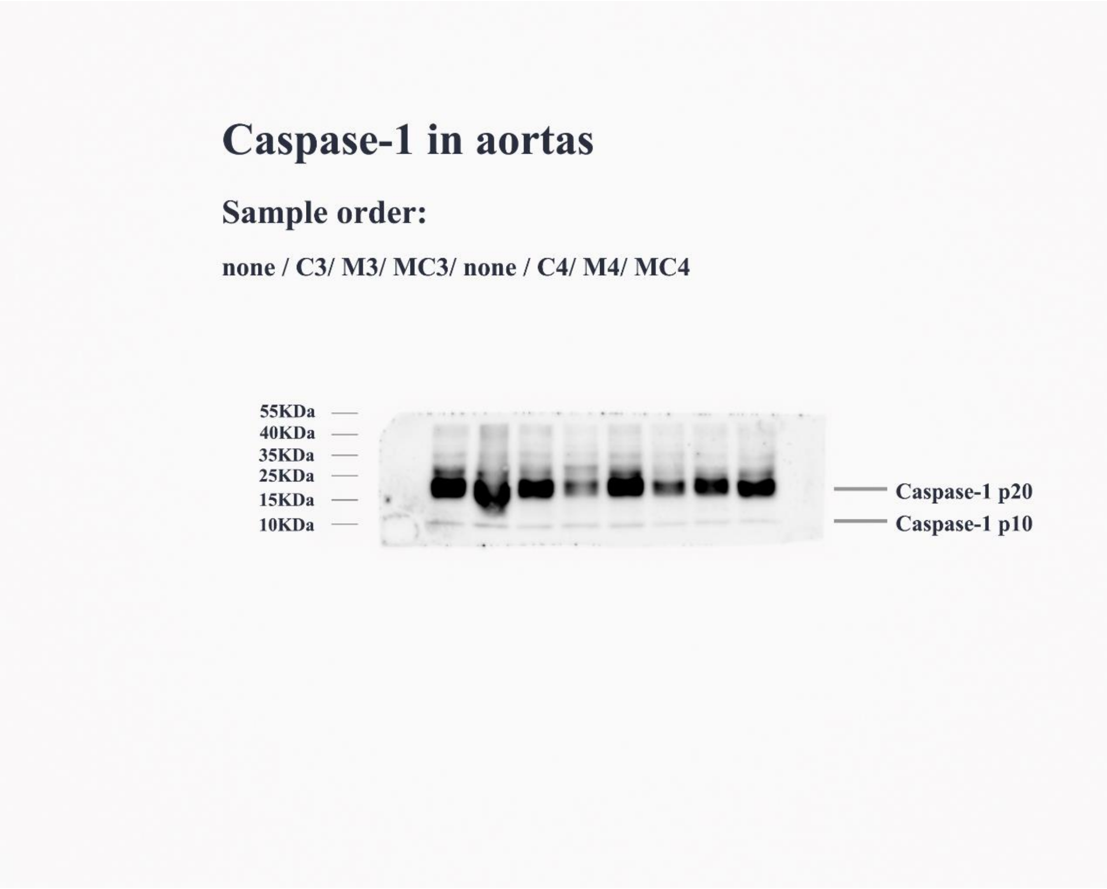

Caspase-1 in aortas

Sample order:

none / C4/ M4/ MC4/ none / C5/ M5/ MC5

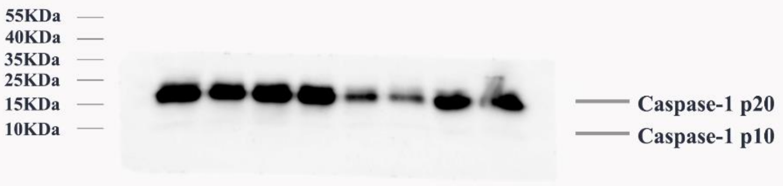

The protein bands of pro-Caspase1 and Caspase-1 in macrophages

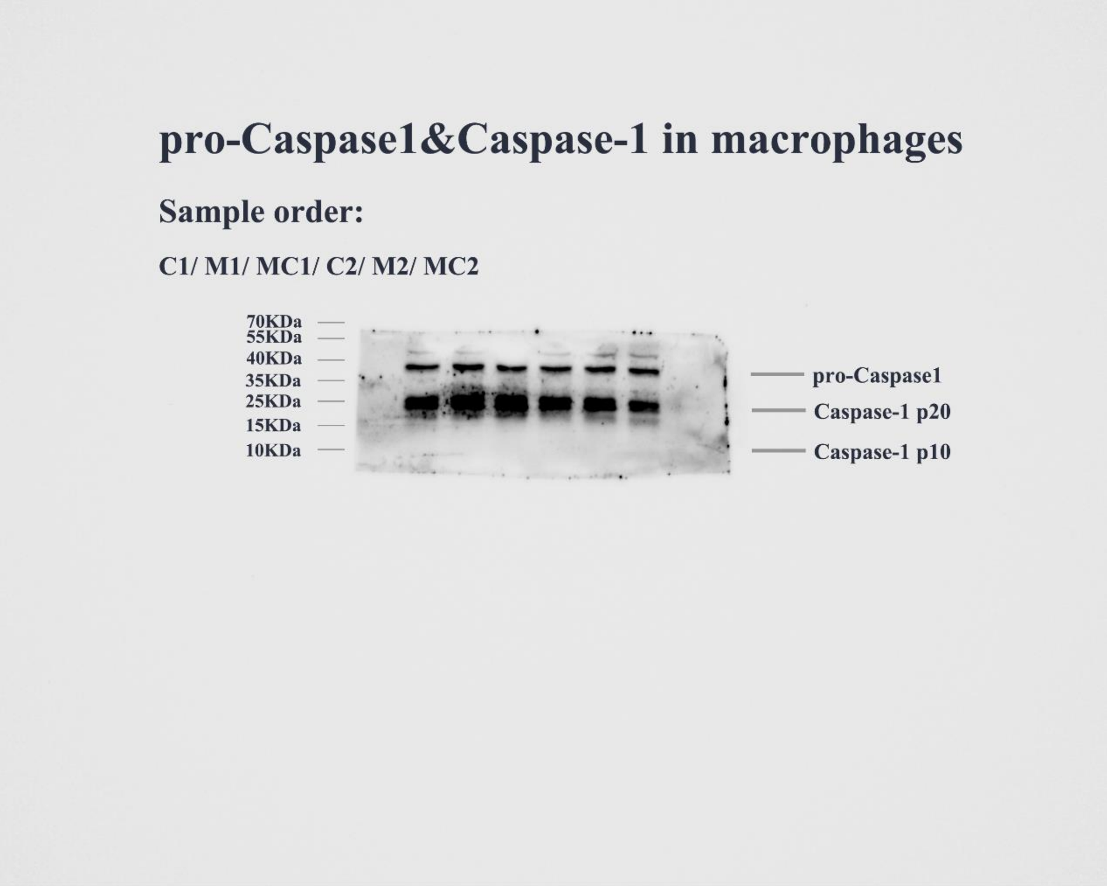

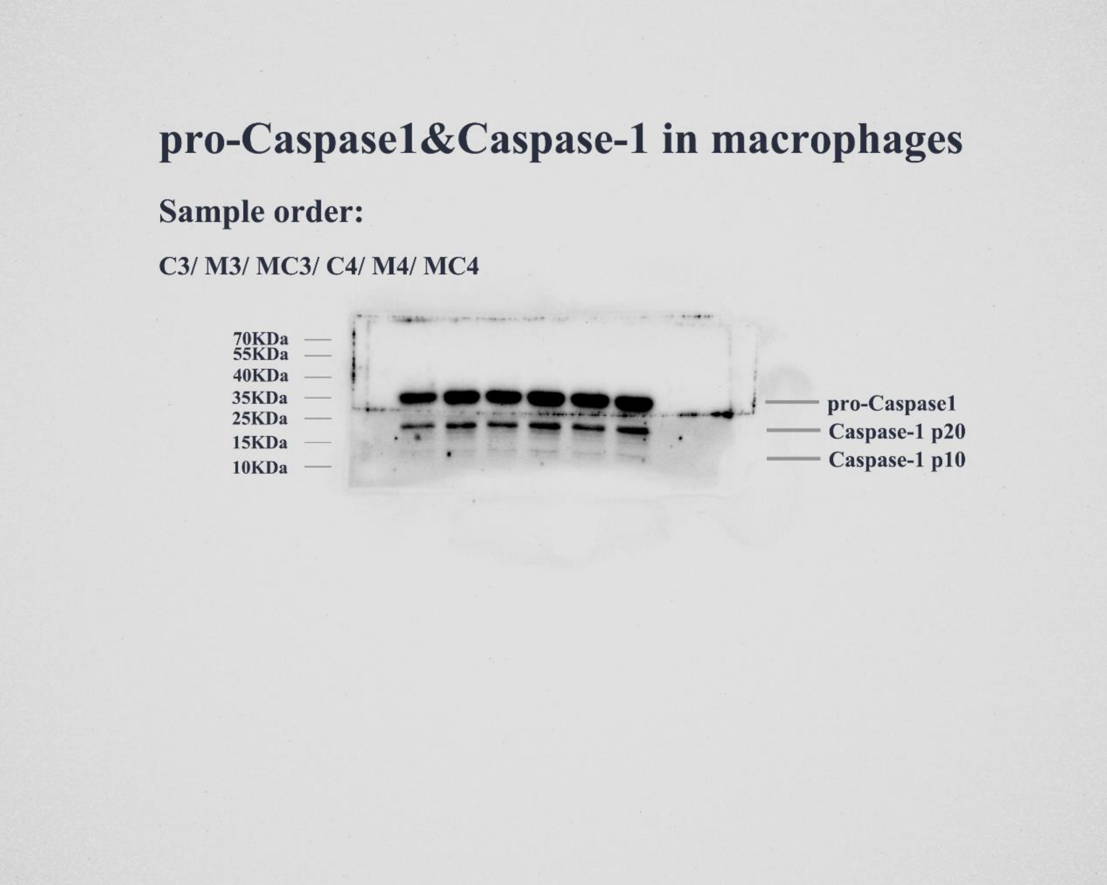

pro-Caspase1&Caspase-1 in macrophages

Sample order:

C4/ M4/ MC4/ C5/ M5/ MC5

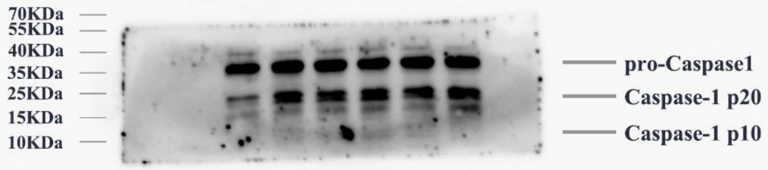

The protein bands of pro-IL1β and IL-1β in aortas

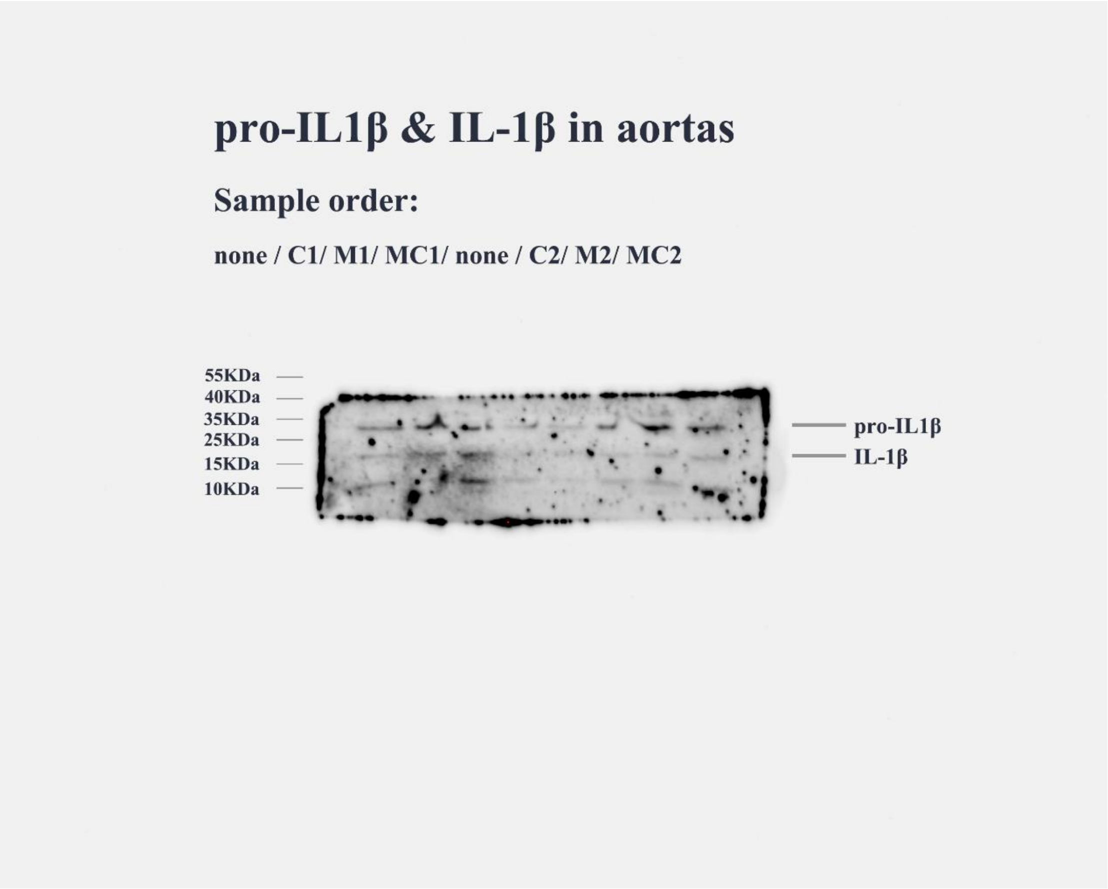

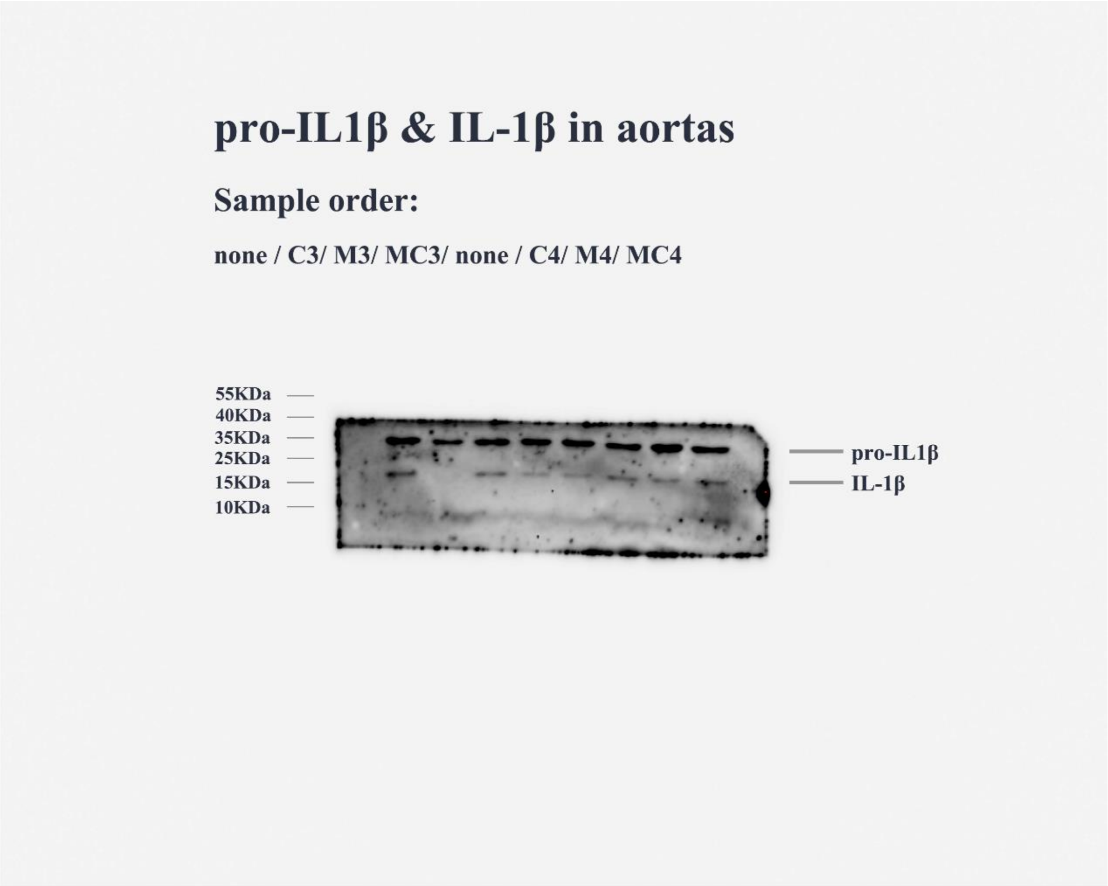

pro-IL1β & IL-1β in aortas

Sample order:

none / C4/ M4/ MC4/ none / C5/ M5/ MC5

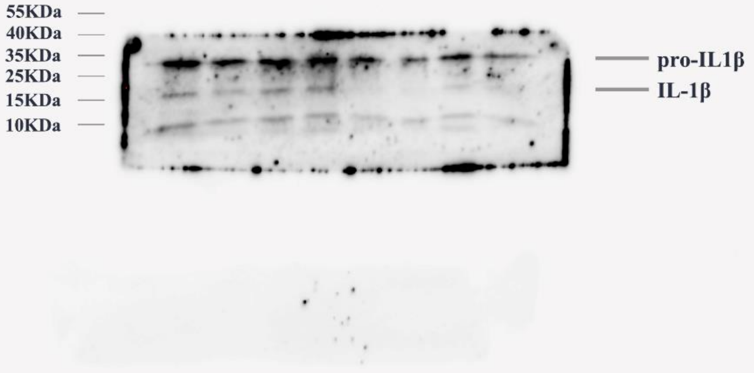

The protein bands of pro-IL1β in macrophages

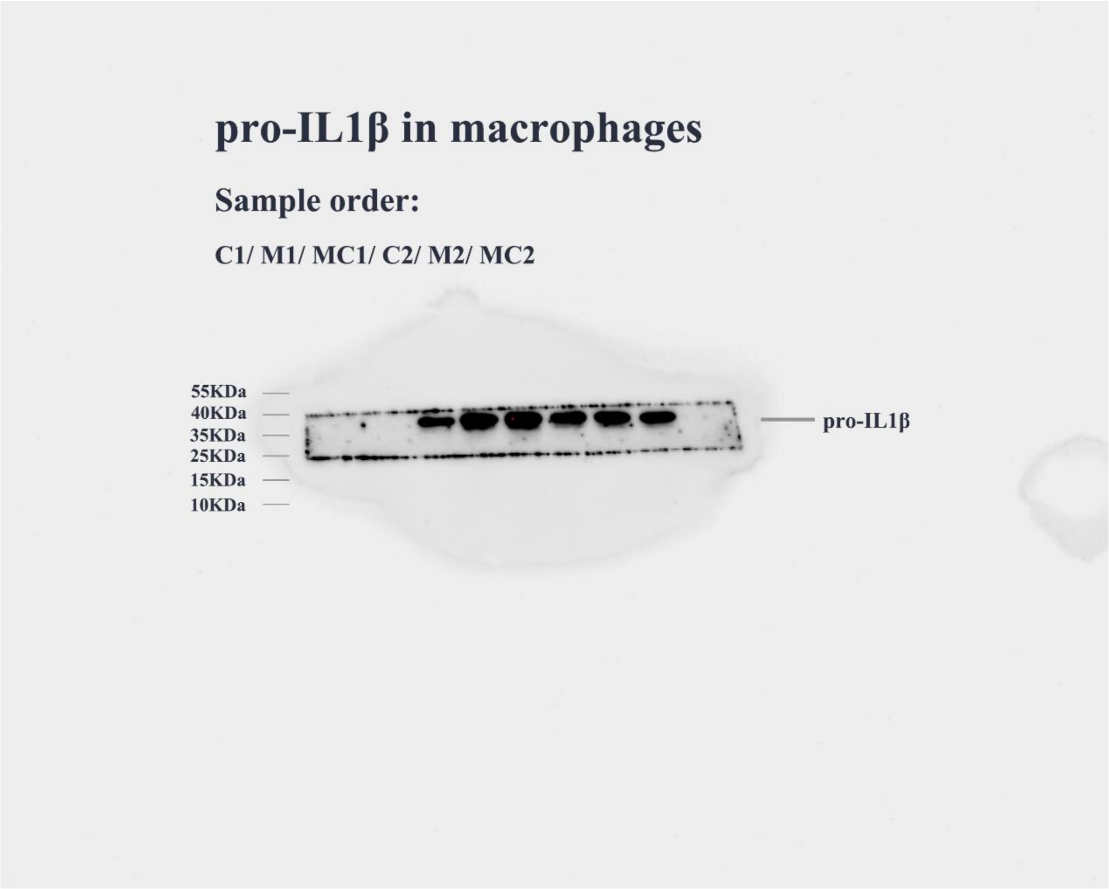

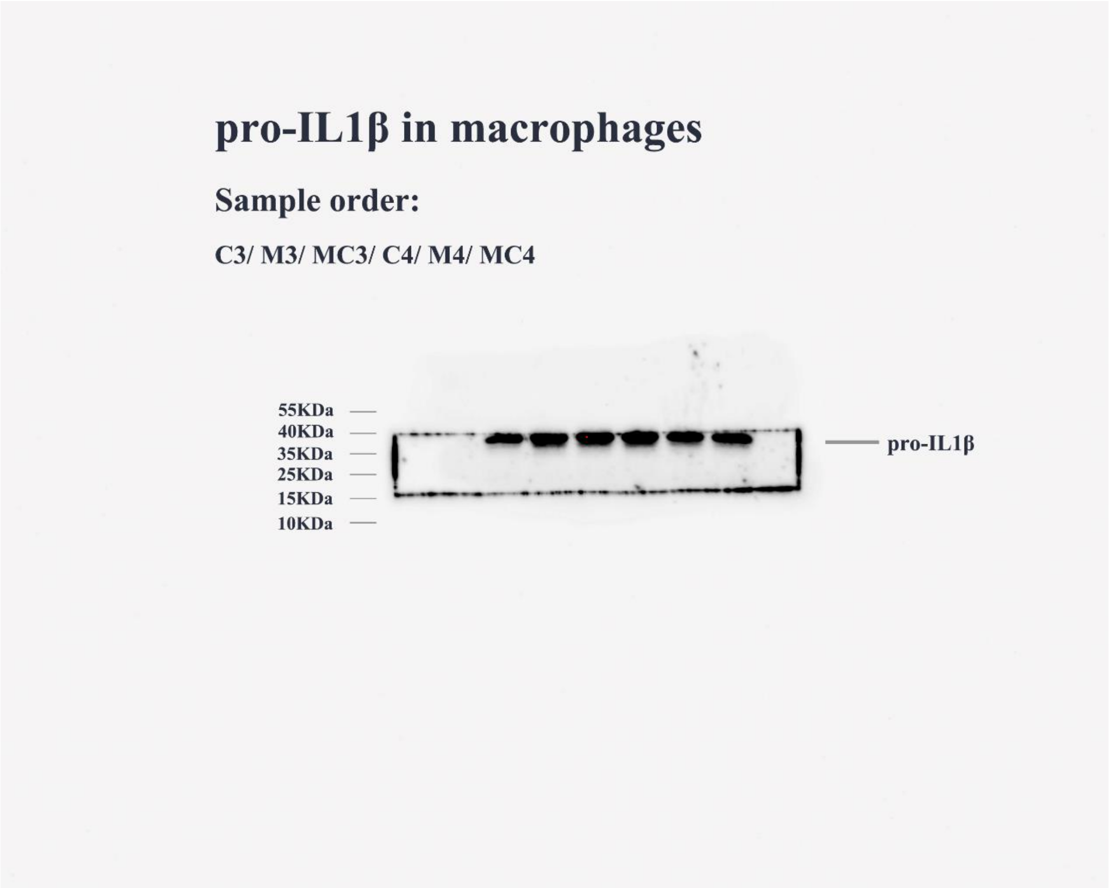

pro-IL1β in macrophages

Sample order:

C4/ M4/ MC4/ C5/ M5/ MC5

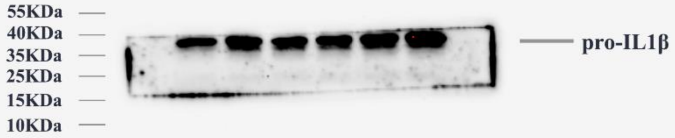

The protein bands of IL-1β in macrophages

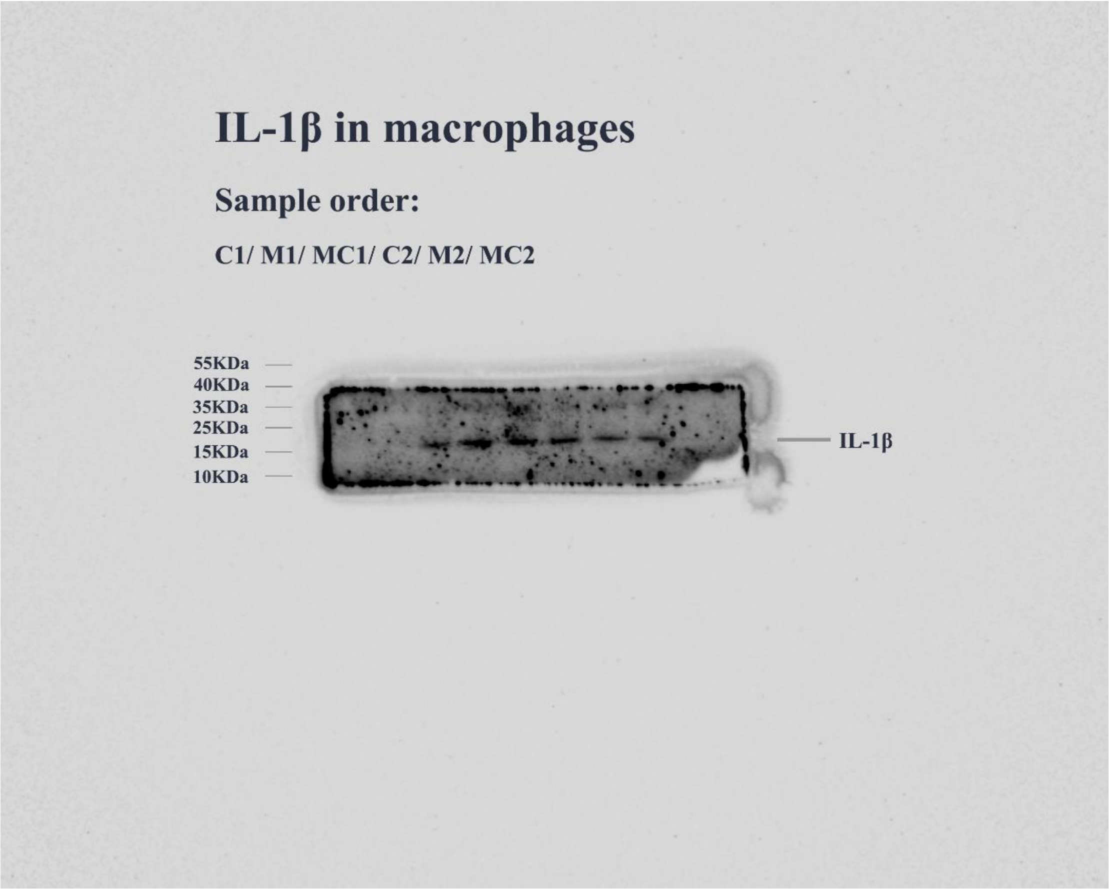

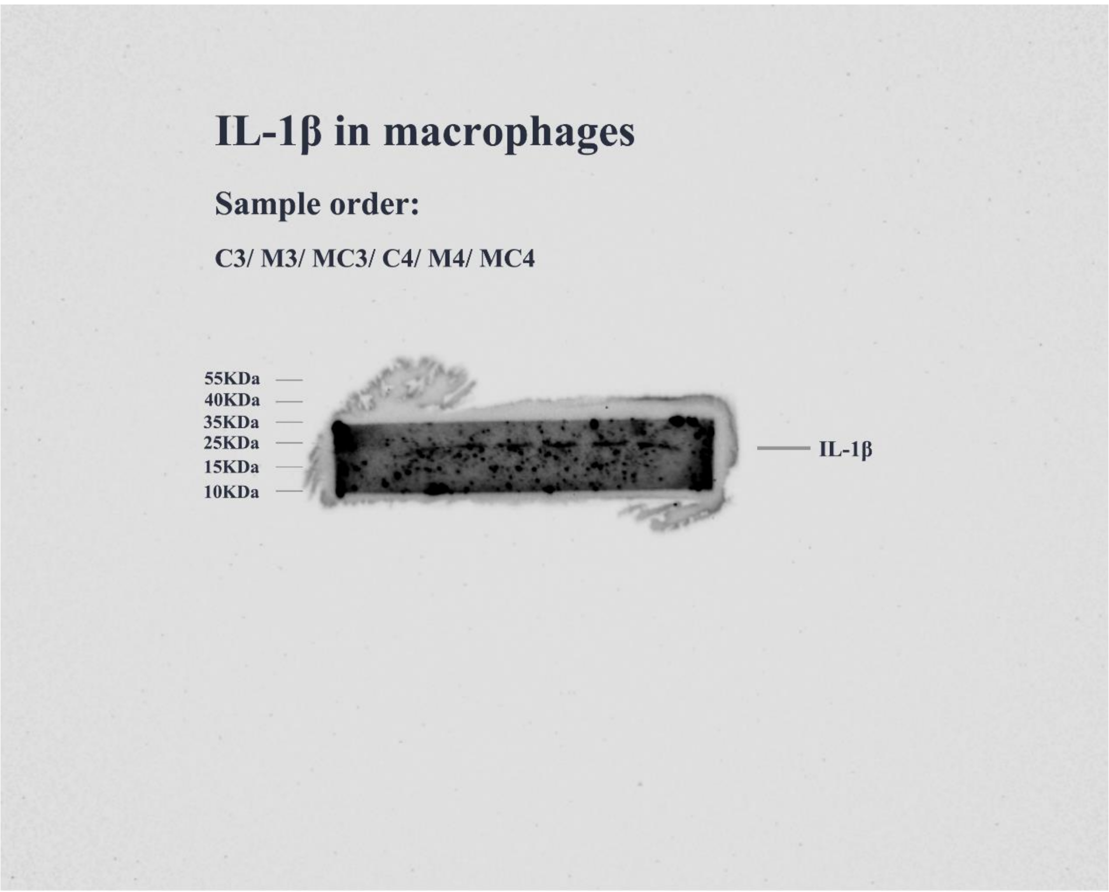

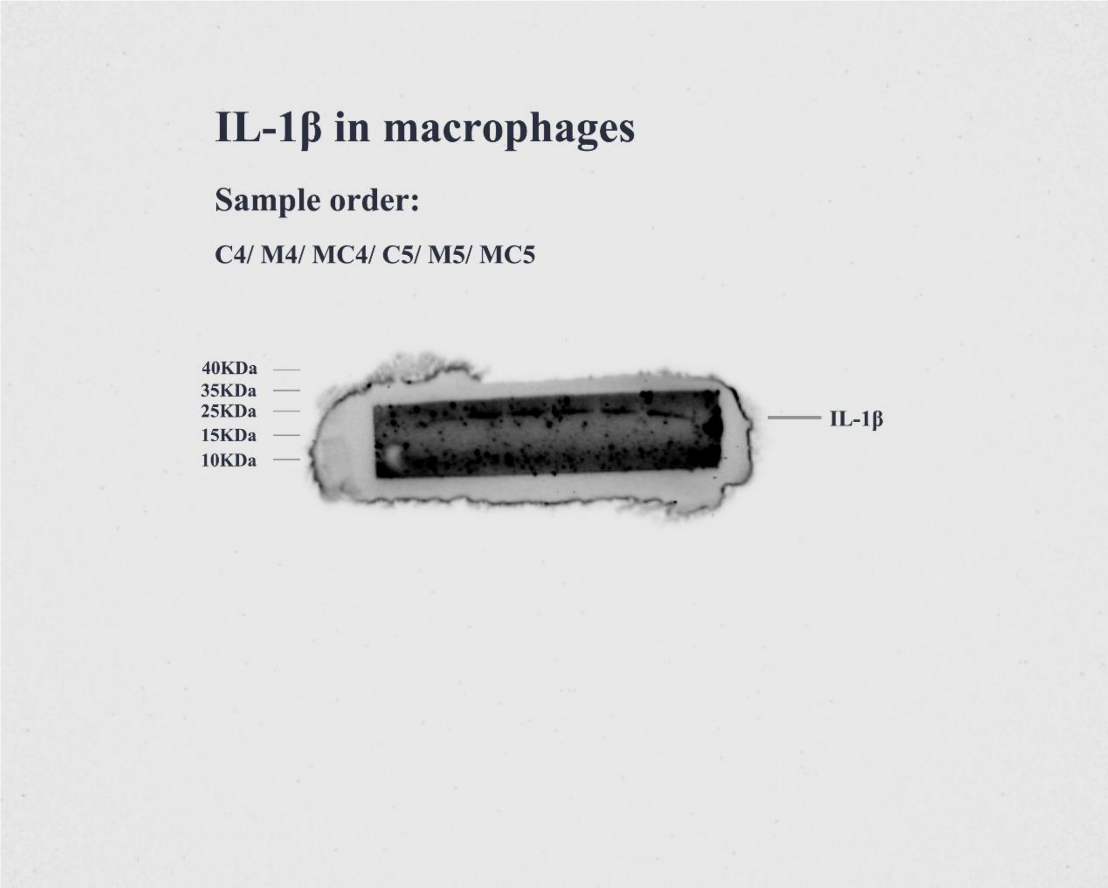
IL-1β in macrophages
Sample order:
C4/ M4/ MC4/ C5/ M5/ MC5
40KDa
35KDa
25KDa
15KDa
10KDa
IL-1β

The protein bands of pro-IL18 and IL-18 in aortas

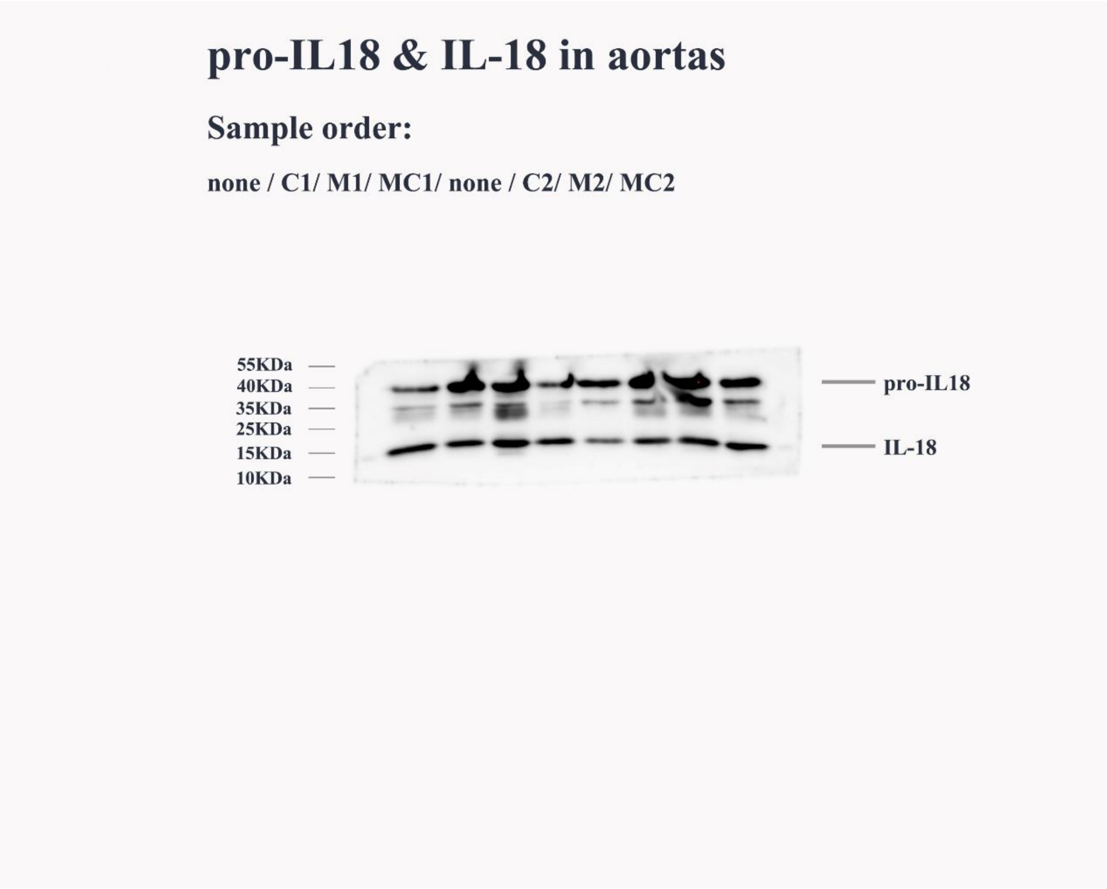

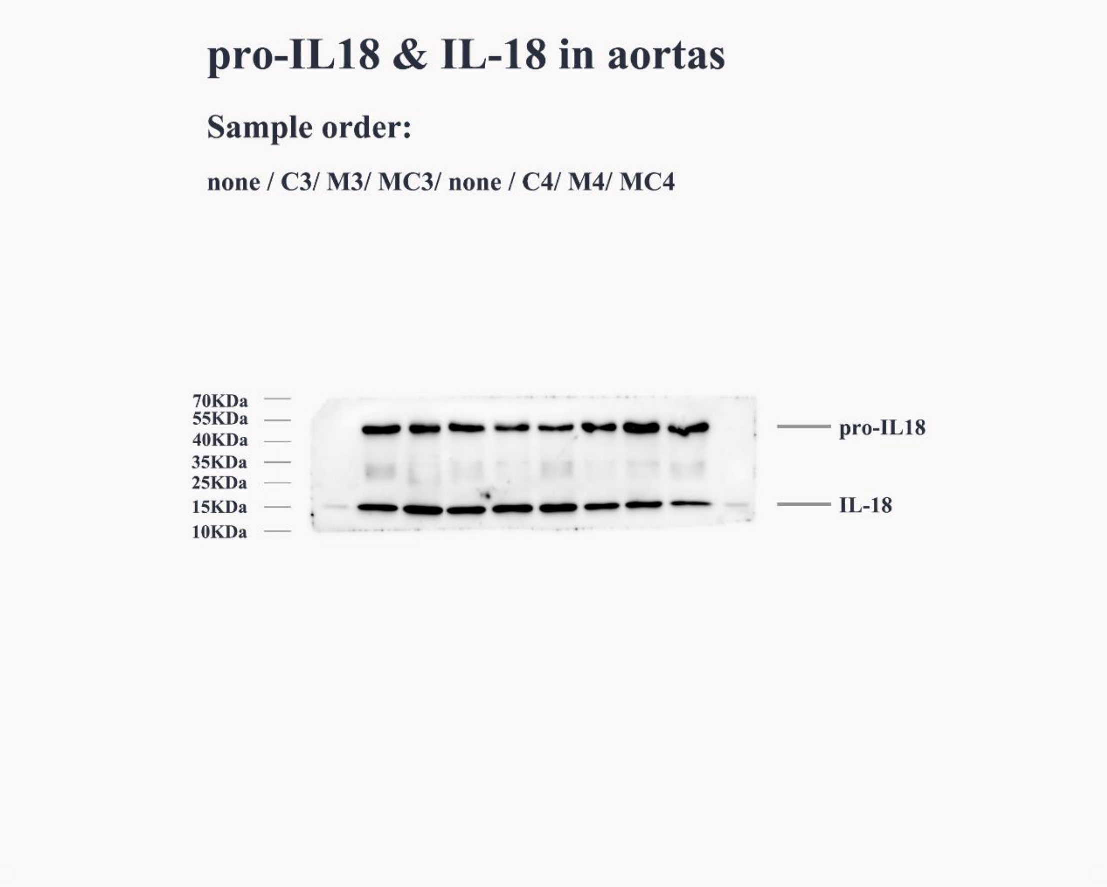

pro-IL18 & IL-18 in aortas

Sample order:

none / C4/ M4/ MC4/ none / C5/ M5/ MC5

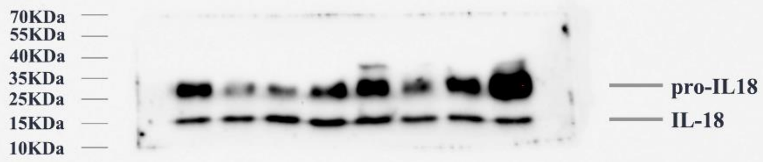

The protein bands of pro-IL18 in macrophages

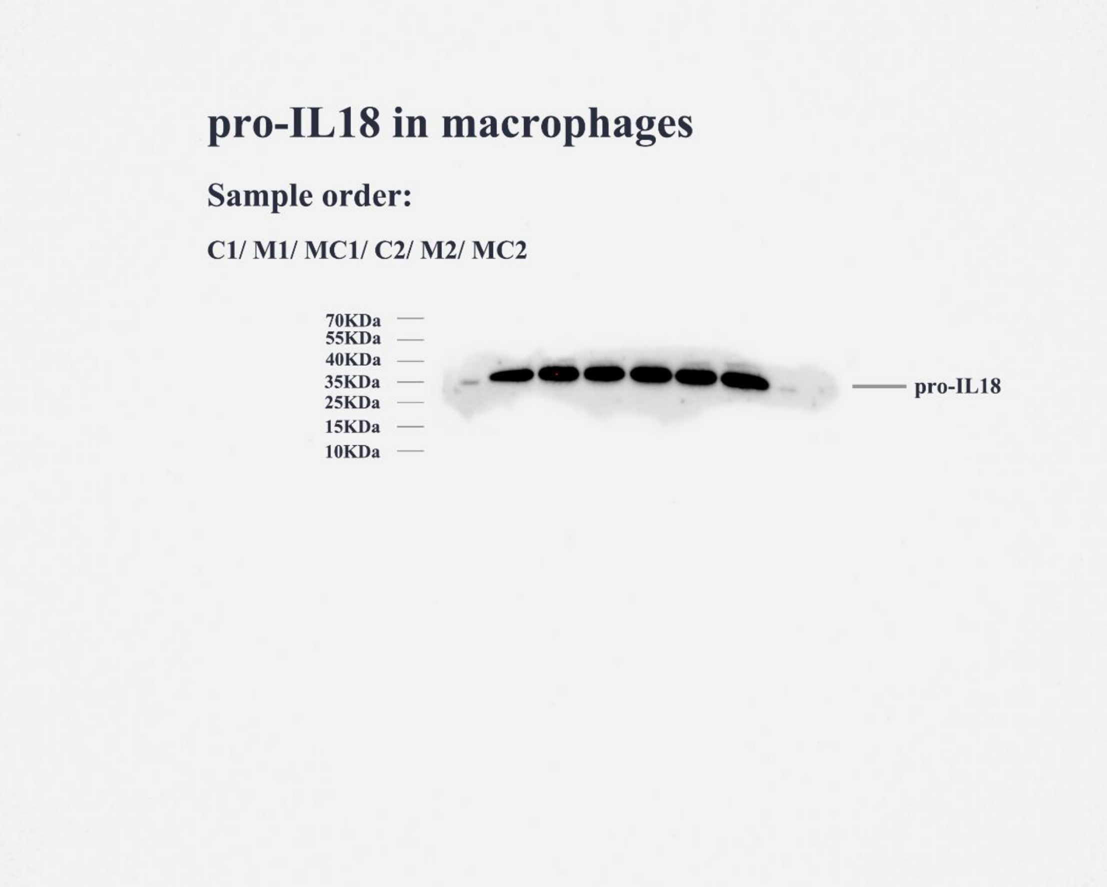

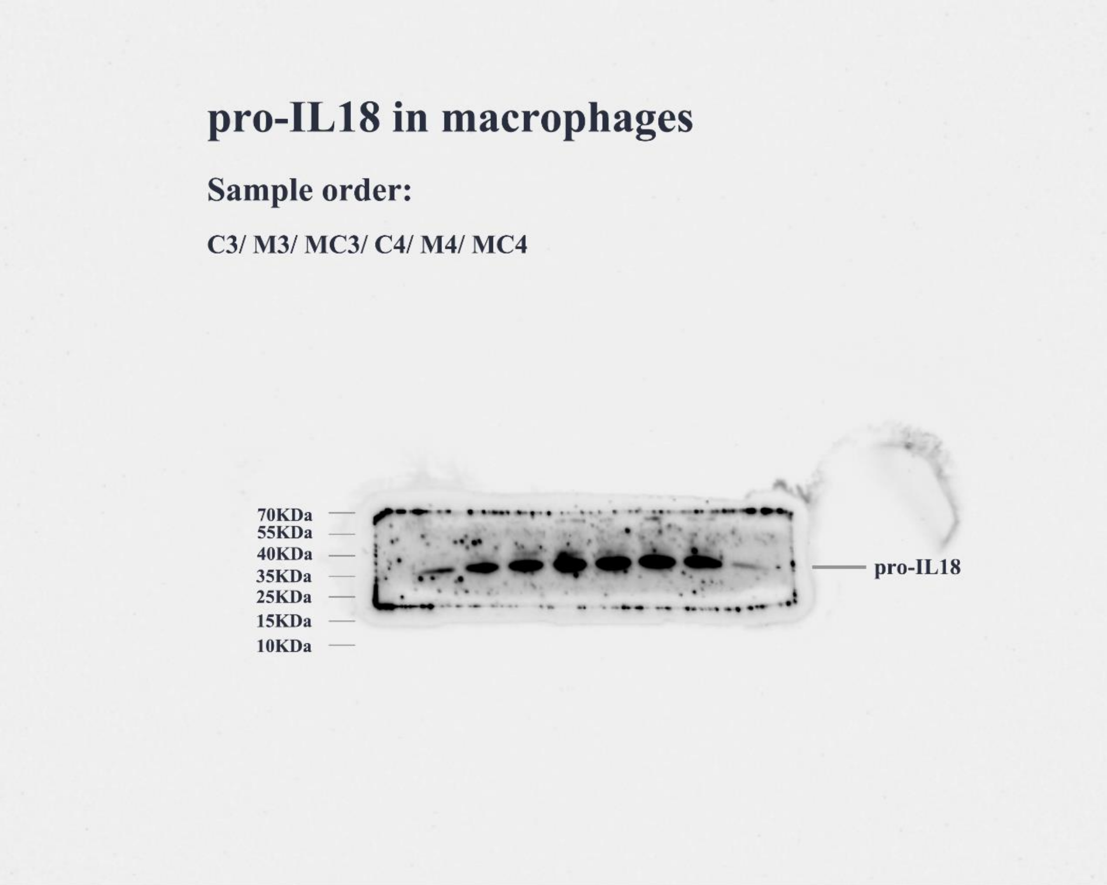

pro-IL18 in macrophages

Sample order:

C4/ M4/ MC4/ C5/ M5/ MC5

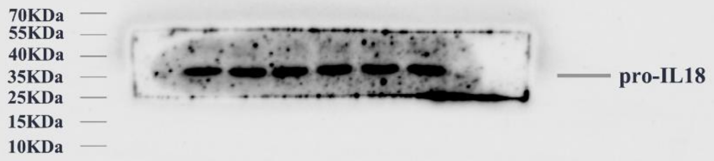

The protein bands of IL-18 in macrophages

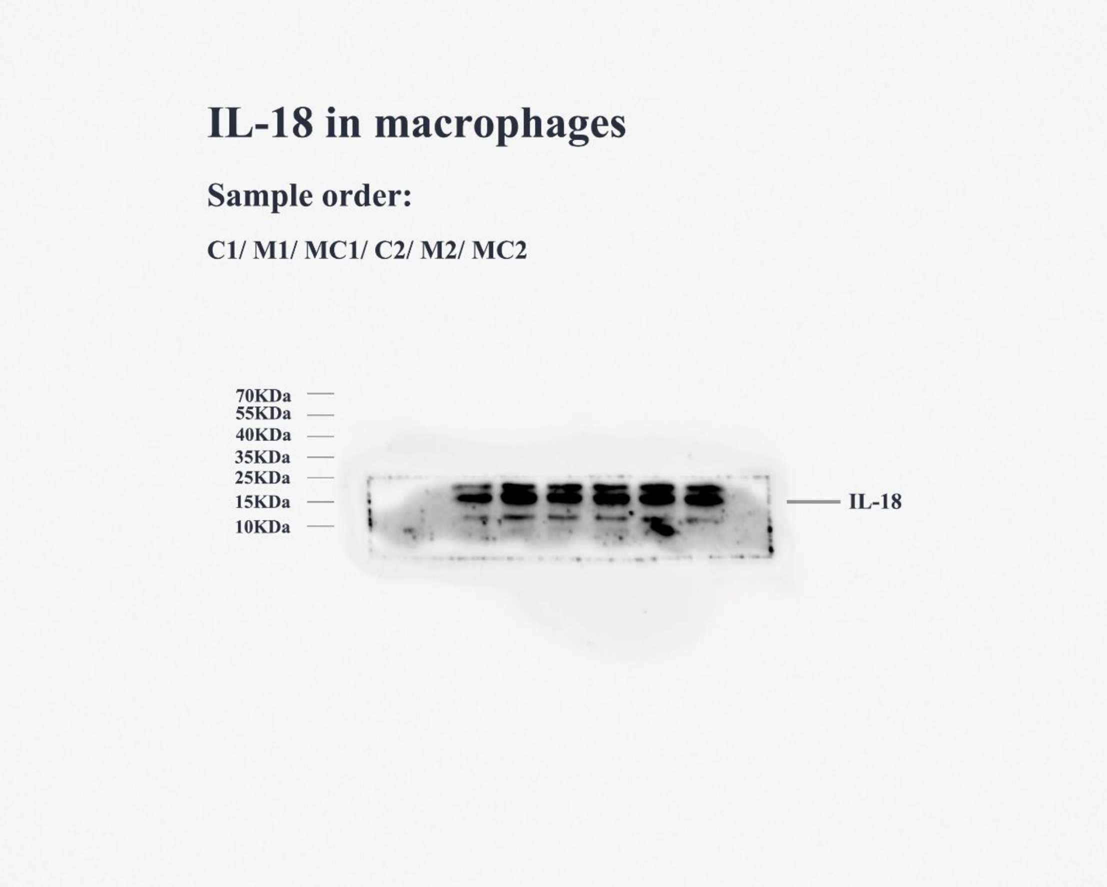

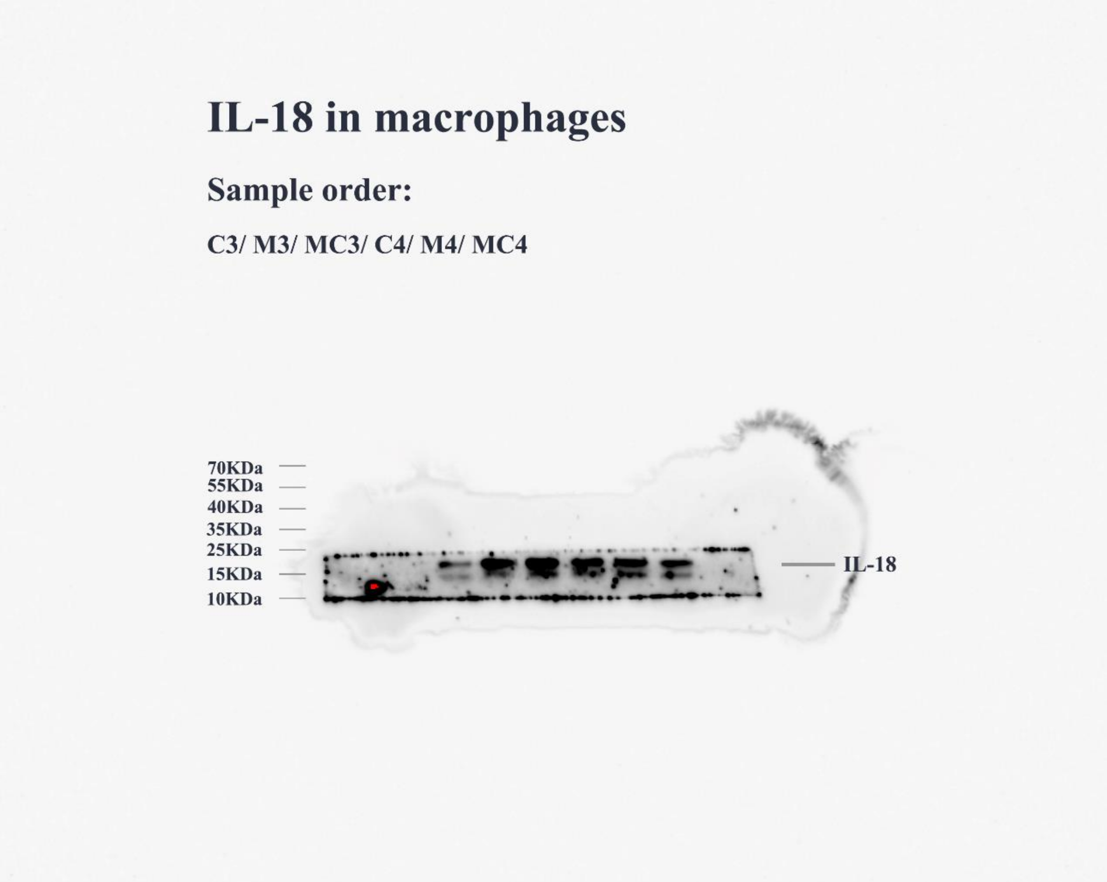

IL-18 in macrophages

Sample order:

C4/ M4/ MC4/ C5/ M5/ MC5

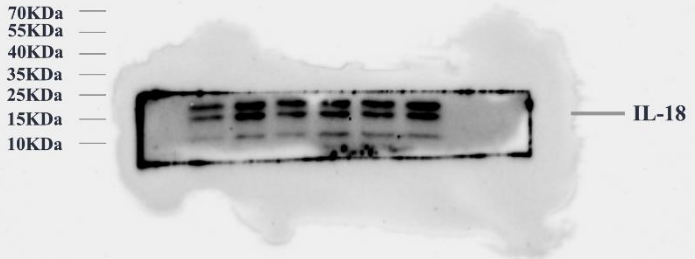

The protein bands of Full-GSDMD in aortas

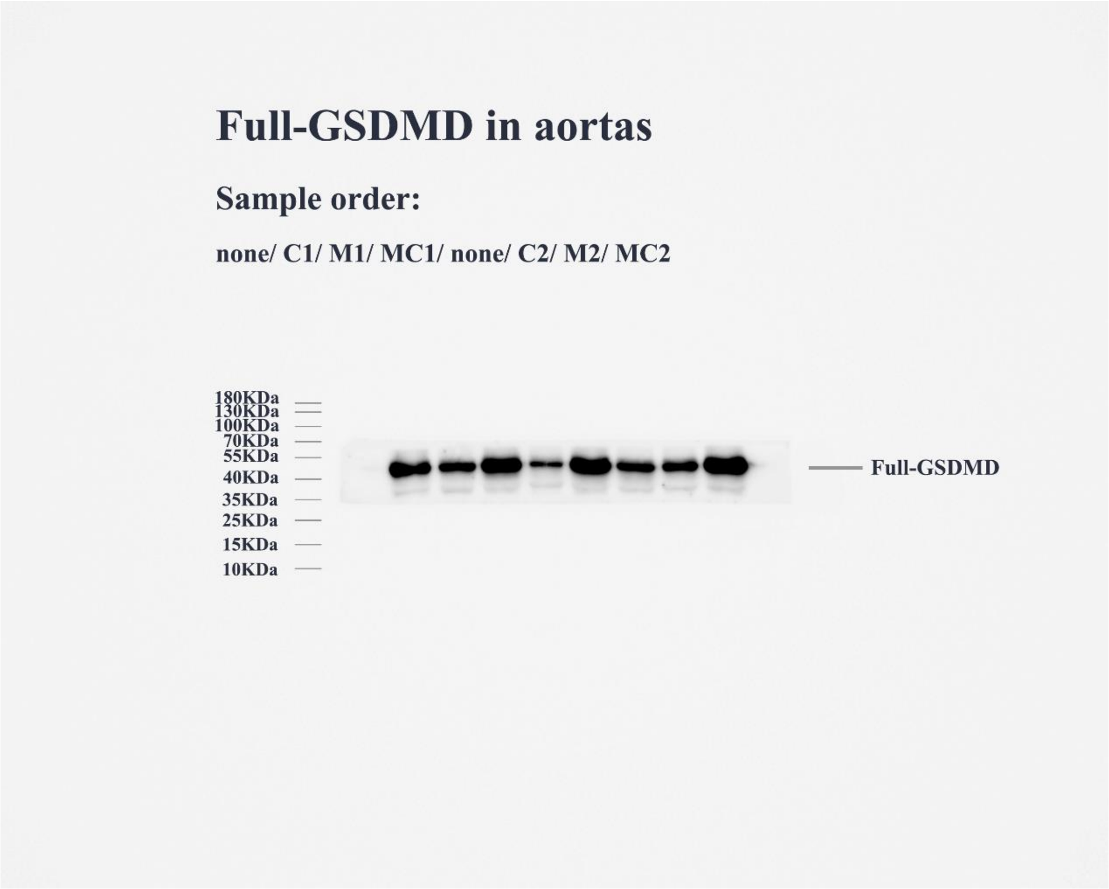

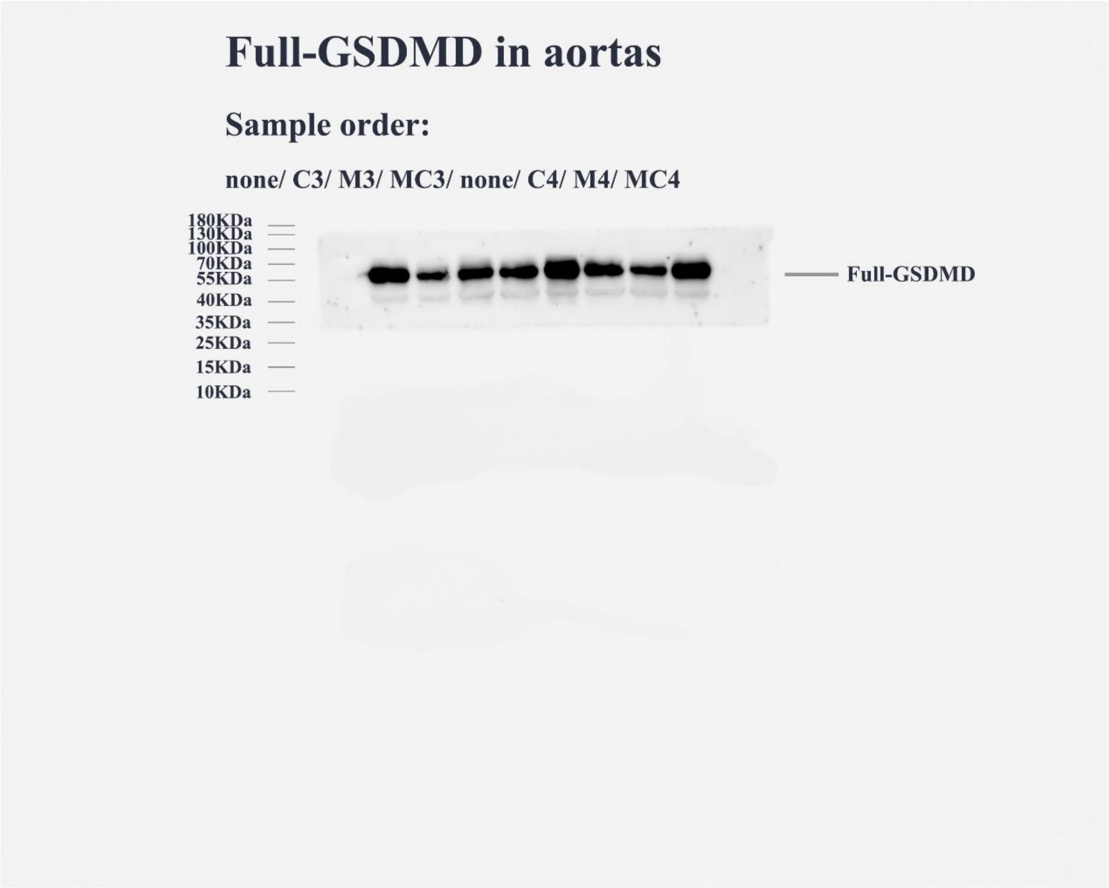

Full-GSDMD in aortas

Sample order:

none/ C4/ M4/ MC4/ none/ C5/ M5/ MC5

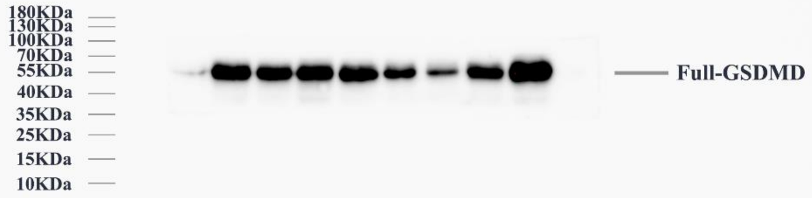

The protein bands of GSDMD-N in aortas

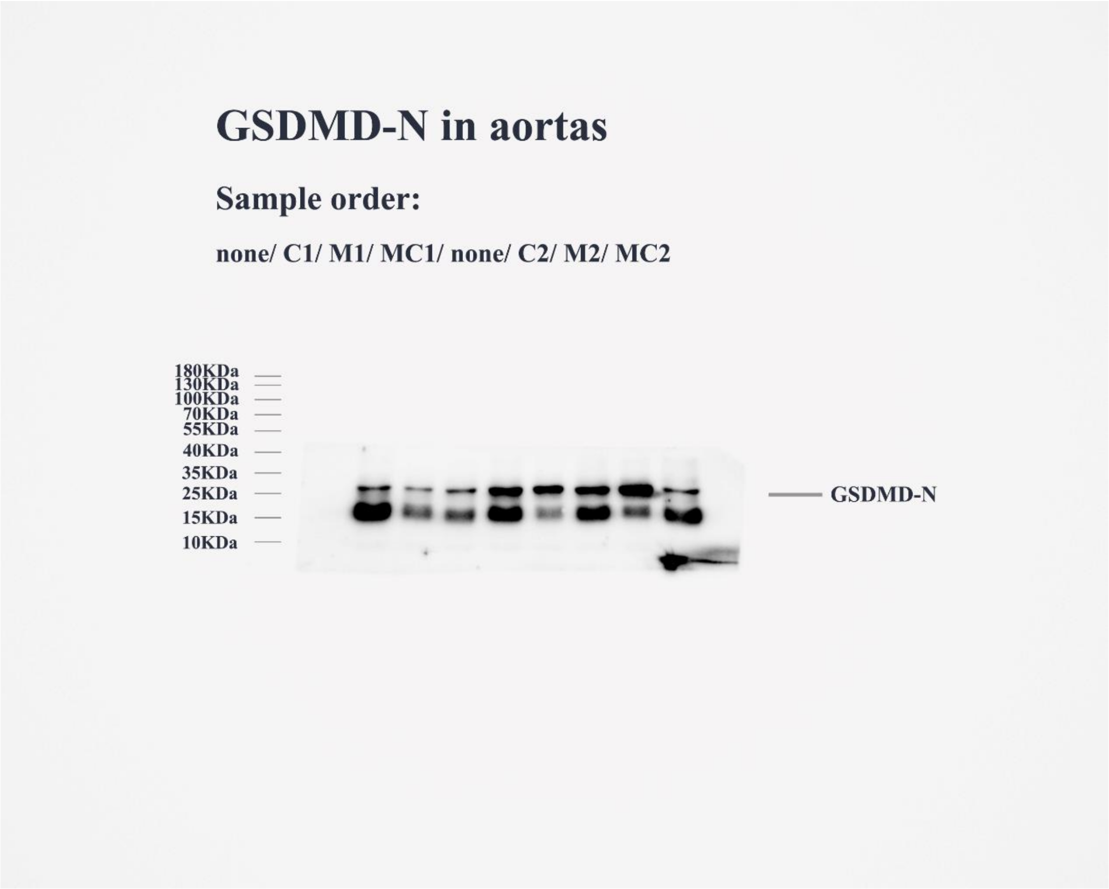

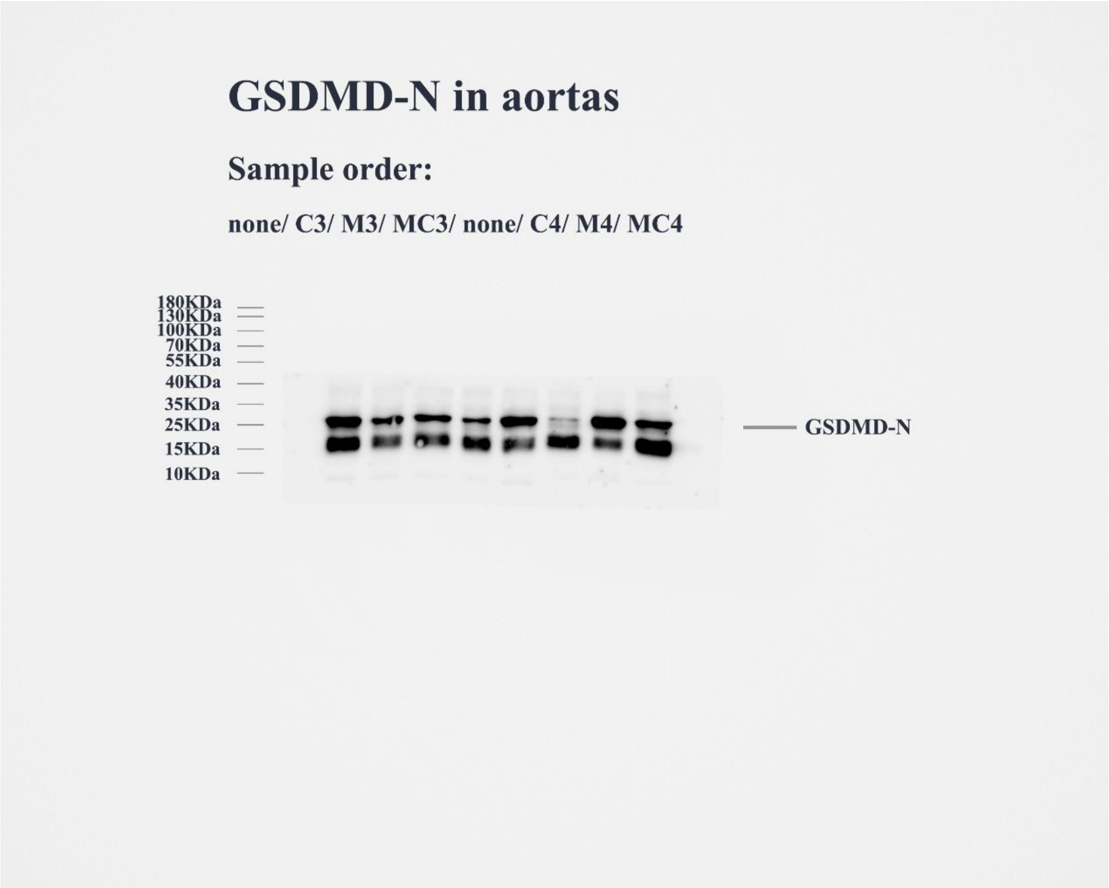

GSDMD-N in aortas

Sample order:

none/ C4/ M4/ MC4/ none/ C5/ M5/ MC5

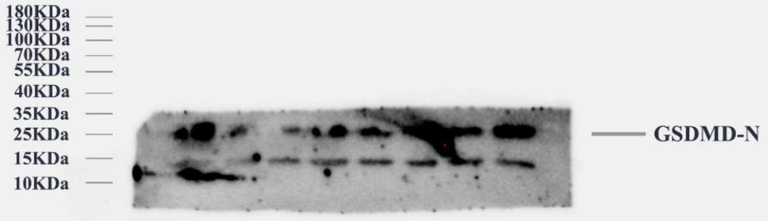

The protein bands of Full-GSDMD and GSDMD-N in macrophages

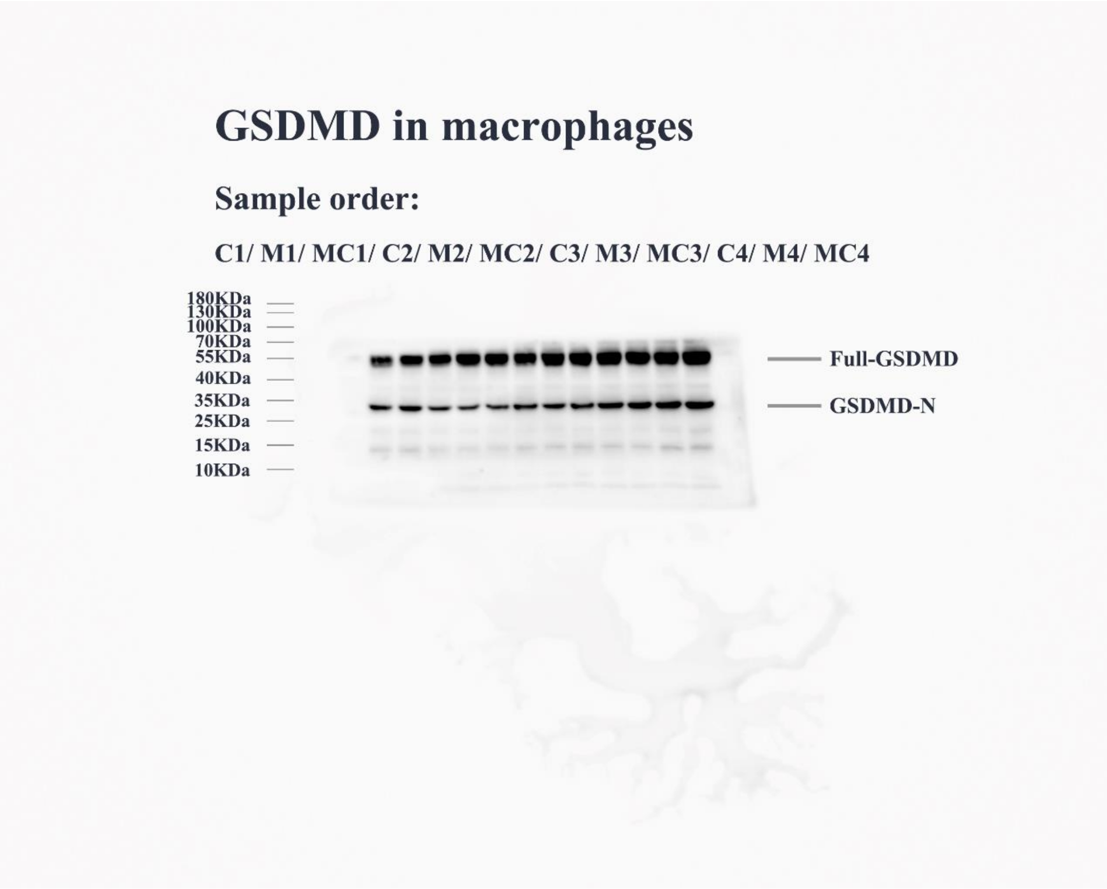

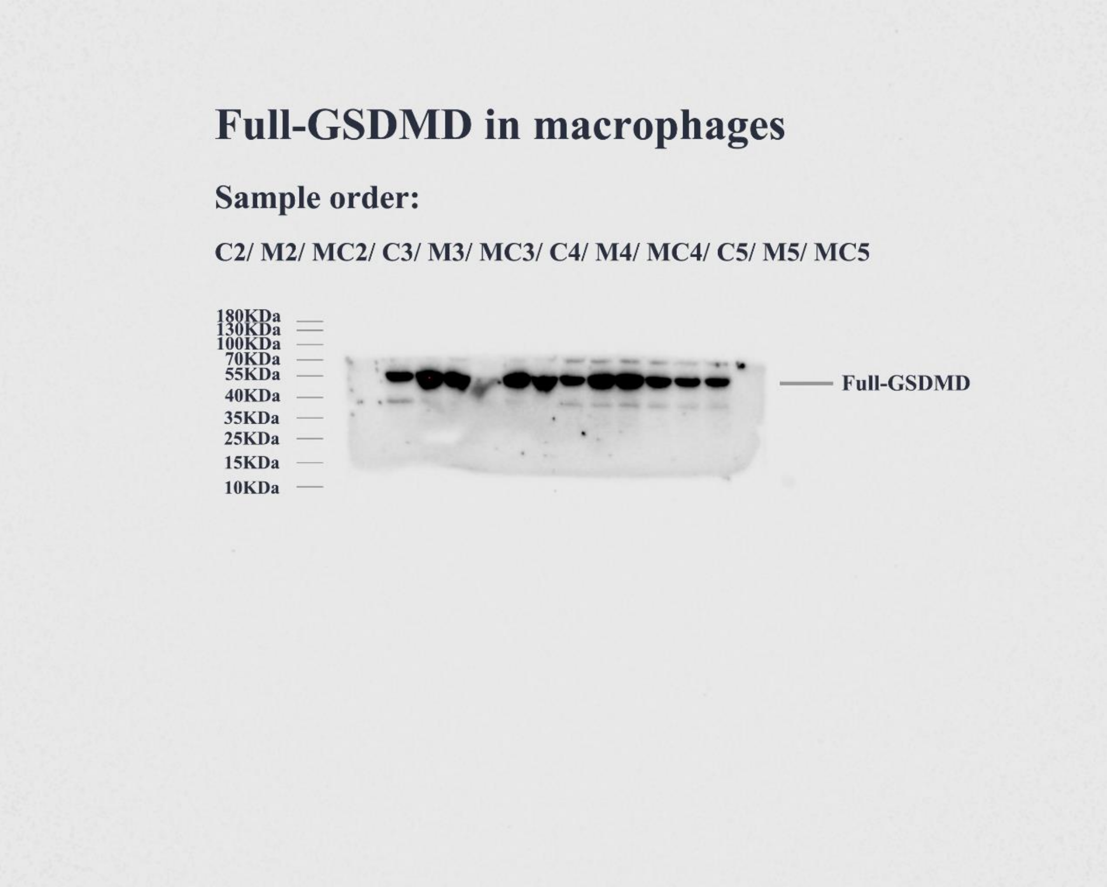

GSDMD-N in macrophages

Sample order:

C2/ M2/ MC2/ C3/ M3/ MC3/ C4/ M4/ MC4/ C5/ M5/ MC5

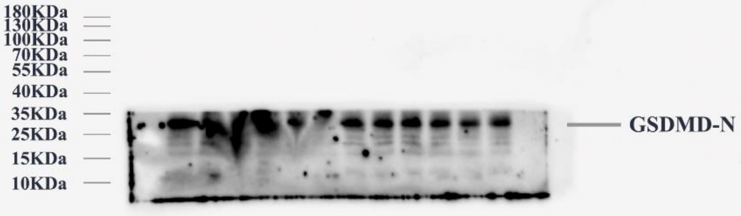

The protein bands of GAPDH in aortas

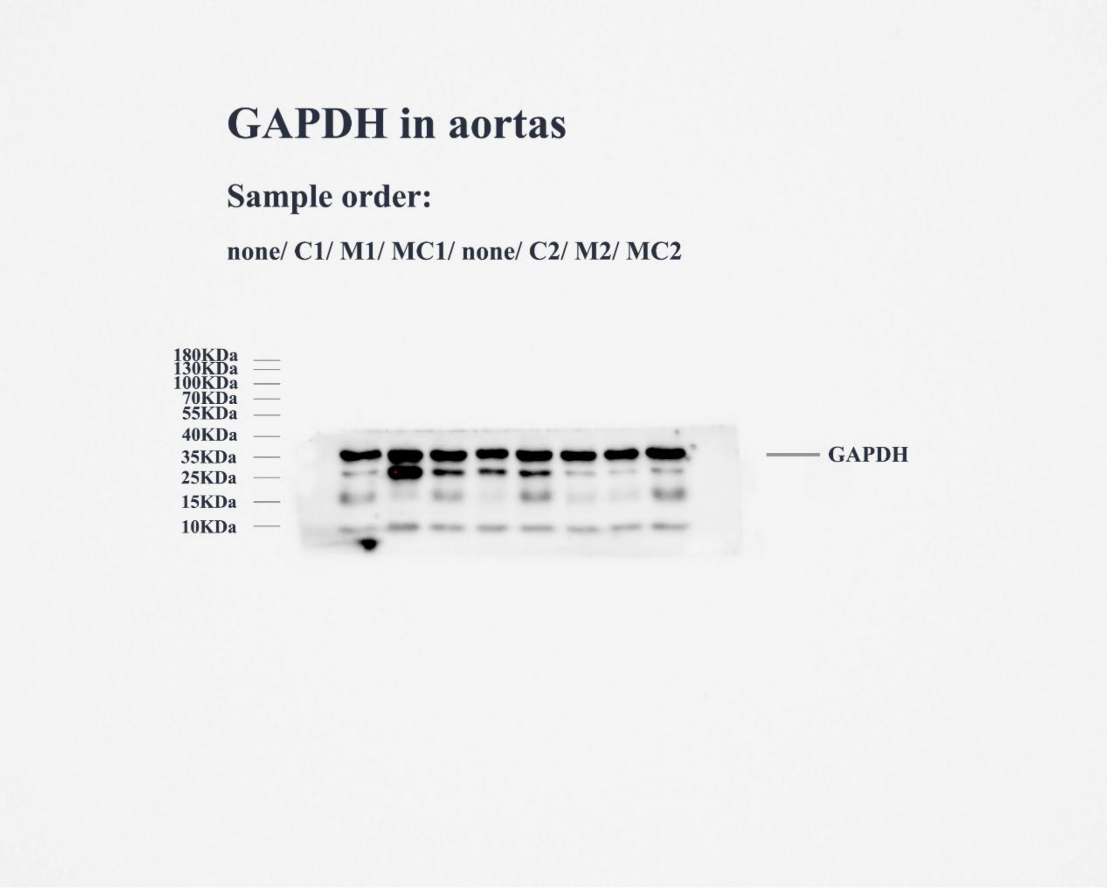

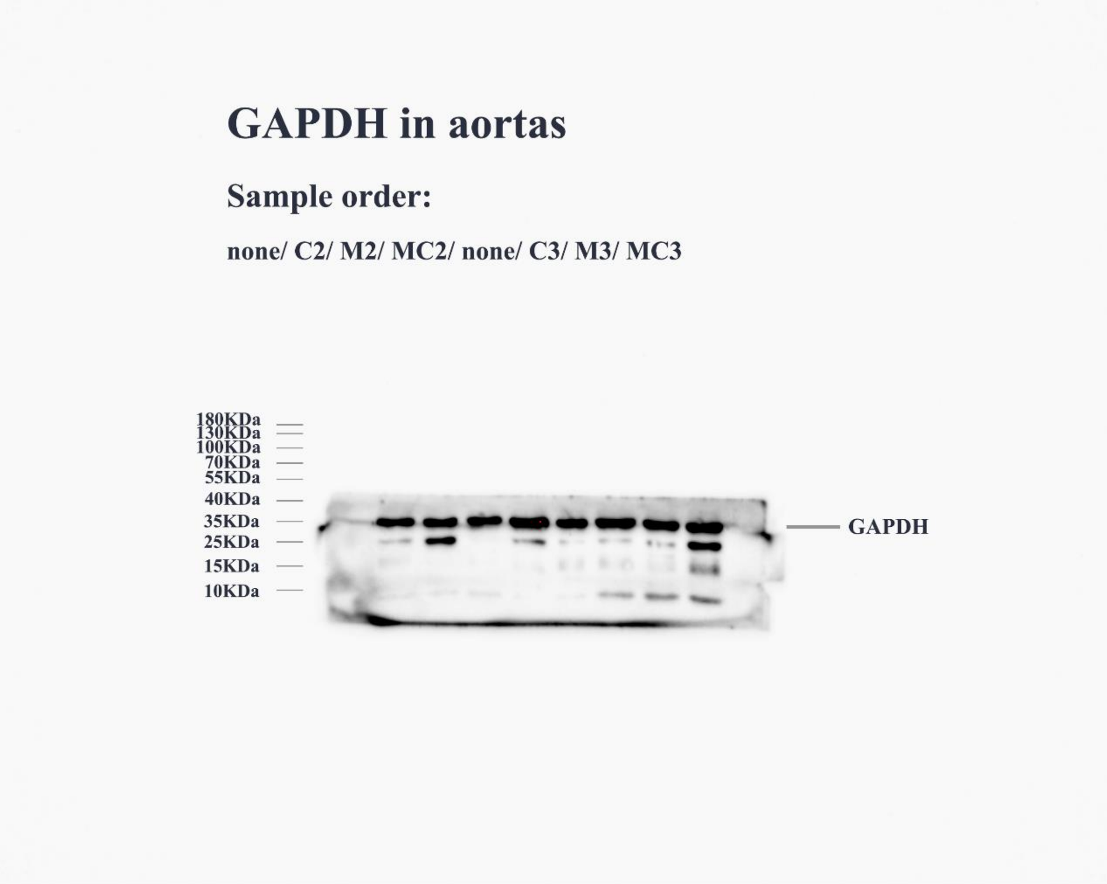

GAPDH in aortas

Sample order:

none/ C3/ M3/ MC3/ none/ C4/ M4/ MC4

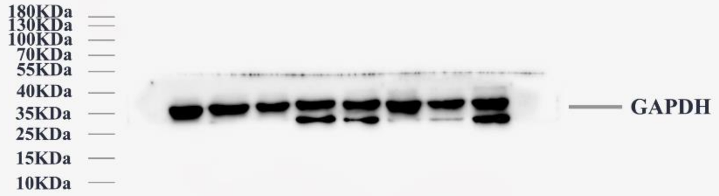

GAPDH in aortas

Sample order:

none/ C4/ M4/ MC4/ none/ C5/ M5/ MC5

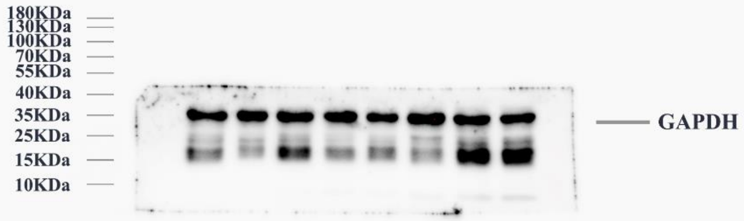

The protein bands of GAPDH in macrophages

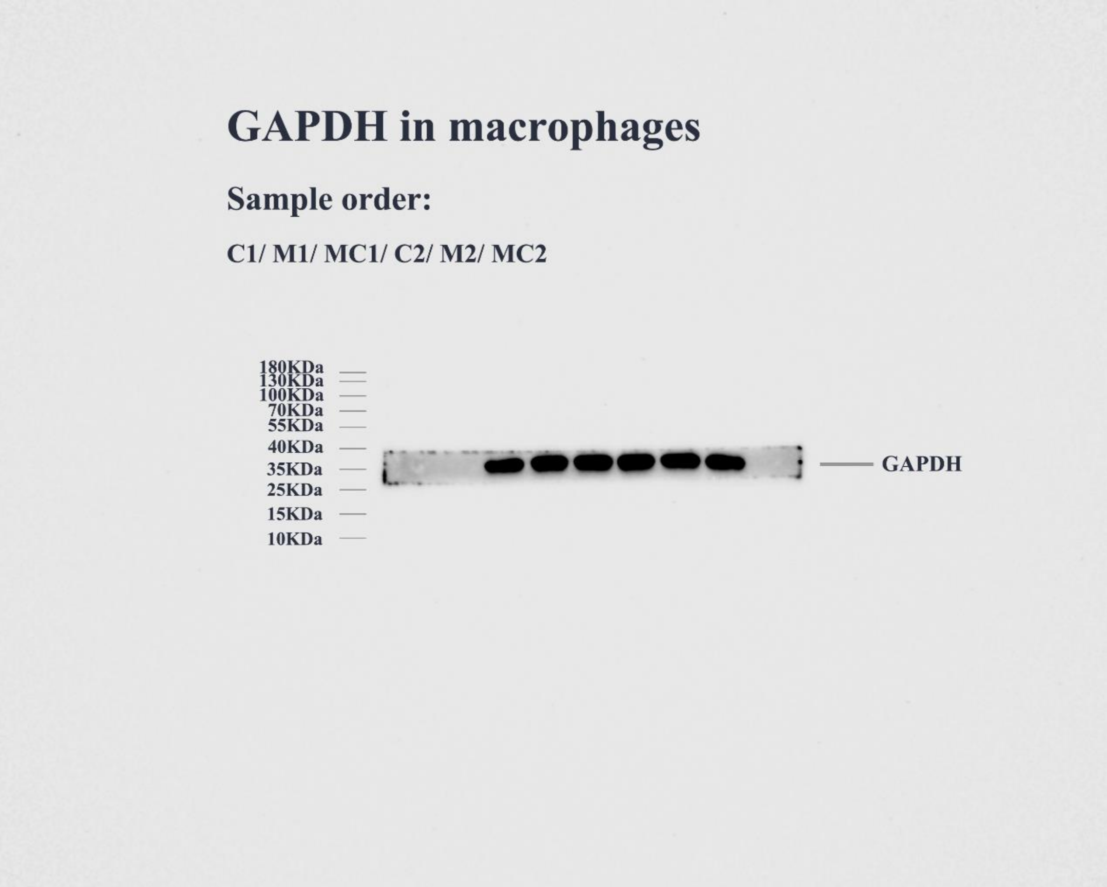

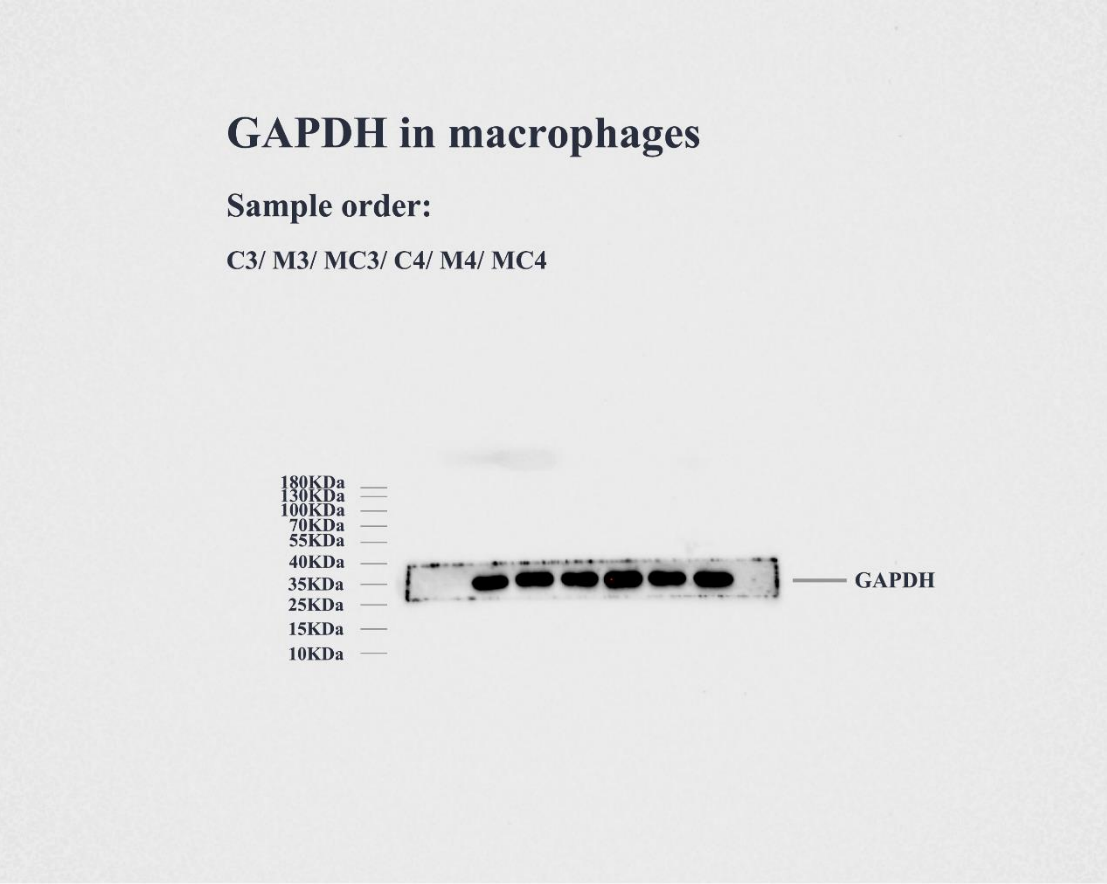

GAPDH in macrophages

Sample order:

C4/ M4/ MC4/ C5/ M5/ MC5

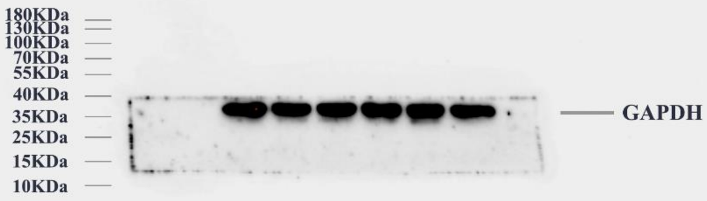

GAPDH in macrophages

Sample order:

C1/ M1/ MC1/ C2/ M2/ MC2/ C3/ M3/ MC3/ C4/ M4/ MC4

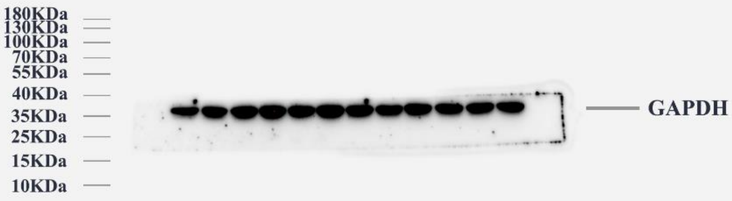

GAPDH in macrophages

Sample order:

C2/ M2/ MC2/ C3/ M3/ MC3/ C4/ M4/ MC4/ C5/ M5/ MC5

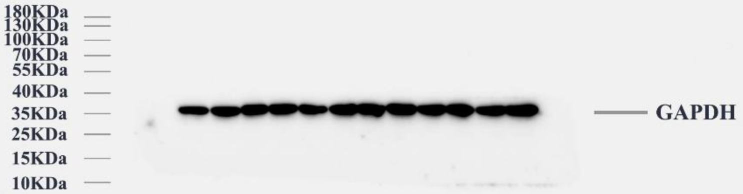

Supplement: Supplementary file 1 — Supplementary Information. [file 41598_2021_98437_MOESM1_ESM.pdf]
